# An IGF1-expressing endometrial stromal cell population is associated with human decidualization

**DOI:** 10.1186/s12915-022-01483-0

**Published:** 2022-12-08

**Authors:** Jia-Wei Shi, Zhen-Zhen Lai, Hui-Li Yang, Wen-Jie Zhou, Xiao-Ya Zhao, Feng Xie, Song-Ping Liu, Wei-Dong Chen, Tao Zhang, Jiang-Feng Ye, Xiang-Yu Zhou, Ming-Qing Li

**Affiliations:** 1 NHC Key Lab of Reproduction Regulation, Hospital of Obstetrics and Gynecology, Shanghai Institute for Biomedical and Pharmaceutical Technologies, Fudan University, Shanghai, 200080 China; 2 Shanghai Key Laboratory of Female Reproductive Endocrine Related Diseases, Hospital of Obstetrics and Gynecology, Fudan University, Shanghai, 200080 China; 3 Center of Reproductive Medicine of Ruijin Hospital, Shanghai Jiao Tong University School of Medicine, Shanghai, 200025 China; 4 Department of Gynecology, International Peace Maternity and Child Health Hospital, Shanghai Jiaotong University School of Medicine, Shanghai, 200030 China; 5 Center for Diagnosis and Treatment of Cervical and Uterine Diseases, Hospital of Obstetrics and Gynecology, Fudan University, Shanghai, 200011 China; 6 Department of Obstetrics and Gynecology, Jinshan Hospital of Fudan University, Shanghai, 201508 China; 7 NovelBio Bio-Pharm Technology Co., Ltd, Shanghai, 201112 China; 8 Assisted Reproductive Technology Unit, Department of Obstetrics and Gynecology, Faculty of Medicine, Chinese University of Hong Kong, Hong Kong, People’s Republic of China; 9 Institute for Molecular and Cell Biology, Agency for Science, Technology and Research, Singapore, 138632 Singapore; 10 State Key Laboratory of Genetic Engineering, Collaborative Innovation Center for Genetics and Development, School of Life Sciences, Fudan University, Shanghai, 200433 People’s Republic of China

**Keywords:** Decidualization, Decidual stromal cells, IGF1, IL1B, IGF1R, Recurrent implantation failure

## Abstract

**Background:**

Decidualization refers to the process of transformation of endometrial stromal fibroblast cells into specialized decidual stromal cells that provide a nutritive and immunoprivileged matrix essential for blastocyst implantation and placental development. Deficiencies in decidualization are associated with a variety of pregnancy disorders, including female infertility, recurrent implantation failure (RIF), and miscarriages. Despite the increasing number of genes reportedly associated with endometrial receptivity and decidualization, the cellular and molecular mechanisms triggering and underlying decidualization remain largely unknown. Here, we analyze single-cell transcriptional profiles of endometrial cells during the window of implantation and decidual cells of early pregnancy, to gains insights on the process of decidualization.

**Results:**

We observed a unique IGF1^+^ stromal cell that may initiate decidualization by single-cell RNA sequencing. We found the IL1B^+^ stromal cells promote gland degeneration and decidua hemostasis. We defined a subset of NK cells for accelerating decidualization and extravillous trophoblast (EVT) invasion by AREG-IGF1 and AREG-CSF1 regulatory axe. Further analysis indicates that EVT promote decidualization possibly by multiply pathways. Additionally, a systematic repository of cell–cell communication for decidualization was developed. An aberrant ratio conversion of IGF1^+^ stromal cells to IGF1R^+^ stromal cells is observed in unexplained RIF patients.

**Conclusions:**

Overall, a unique subpopulation of IGF1^+^ stromal cell is involved in initiating decidualization. Our observations provide deeper insights into the molecular and cellular characterizations of decidualization, and a platform for further development of evaluation of decidualization degree and treatment for decidualization disorder-related diseases.

**Supplementary Information:**

The online version contains supplementary material available at 10.1186/s12915-022-01483-0.

## Background

Reproduction is critical to the survival of a species, but there is a surprisingly high failure rate (approximately 70% during one menstrual cycle) of blastocyst implantation in humans [1]. Successful blastocyst implantation requires a synchronized and coordinated crosstalk between the seed (embryo) and the soil (endometrium) [2, 3]. Decidualization refers to the transformation of endometrial stromal fibroblast cells (ESCs) into specialized decidual stromal cells (DSCs) that provide a nutritive and immune privileged matrix essential for blastocyst implantation and placental development [1, 4, 5]. Deficiencies in decidualization are associated with a variety of pregnancy disorders, including female infertility, miscarriages, intrauterine growth restriction, and preeclampsia [1–7].

Unlike most mammals, decidualization of the human endometrium starts and occurs to some extent throughout the functional layer of the endometrium during the secretory phase of the menstrual cycle, even in the absence of embryo implantation, but become full blown in early pregnancy [1, 8]. During early pregnancy, this process is traditionally driven by the postovulatory rise in progesterone levels and increased local cyclic adenosine monophosphate (cAMP) production, along with the elevation of marker molecules prolactin (PRL) and insulin-like growth factor binding protein 1 (IGFBP-1). Generalized decidualization also includes secretion of the endometrial luminal and glandular epithelium, recruitment and redistribution of immune cells, remodeling of uterine spiral artery, and change of extracellular matrix components [8–14]. More importantly, embryonic trophoblast cells have also been reported to play an important role in accelerating decidualization in humans [15, 16]. Unfortunately, the cellular, molecular, and spatiotemporal regulatory mechanisms of decidualization in humans still remain largely unknown, especially mechanism of triggering decidualization. Despite significant advances in assisted reproductive technology, even with high-quality embryos, repeated implantation failure (RIF) and repeated pregnancy loss (RPL) due to defective decidualization cannot yet be effectively avoided [1, 17, 18].

During the last decades, more and more genes associated with endometrial receptivity / decidualization have been reported by microarray and RNA sequence technique in whole-tissue and cell transcriptomic analysis [19, 20]. Although it remains controversial [21, 22], these techniques have been translated into clinical practice to evaluate the endometrial receptivity / decidualization and determine the window of implantation (WOI) timing for in vitro fertilization-embryo transfer (IVF-ET) [19, 20, 23], and performed to screen for new genetic and chemical modulators of decidualization [24].

Recent studies have employed single-cell RNA sequencing (scRNA-seq) technology to investigate the cellular composition and intercellular communication of endometrium that occur across the human menstrual cycle [25, 26], or human decidua during early pregnancy [27, 28]. In vitro decidualization system, Lucas et al. evaluated the decidual pathway by scRNA-seq [29]. These studies above provide us with great enlightenment of endometrial receptivity and decidualization. However, the composition changes (cell types and molecular profiles) of endometrium before and after embryo implantation, and detailed understanding of molecular characterization, cellular communication, and spatiotemporal regulatory networks during decidualization are largely unknown. In this study, we profiled the endometrial cells present at the WOI timing and decidual cells of early pregnancy both from healthy donors, and provided a continuous and systematic single-cell transcriptomic atlas of decidualization in human before and after embryo implantation, and defined a unique insulin-like growth factor 1 (IGF1)^+^ stromal cell probably for triggering endometrial decidualization.

## Results

### An atlas of human endometrium from non-pregnant and pregnant women

**Fig. 1:**
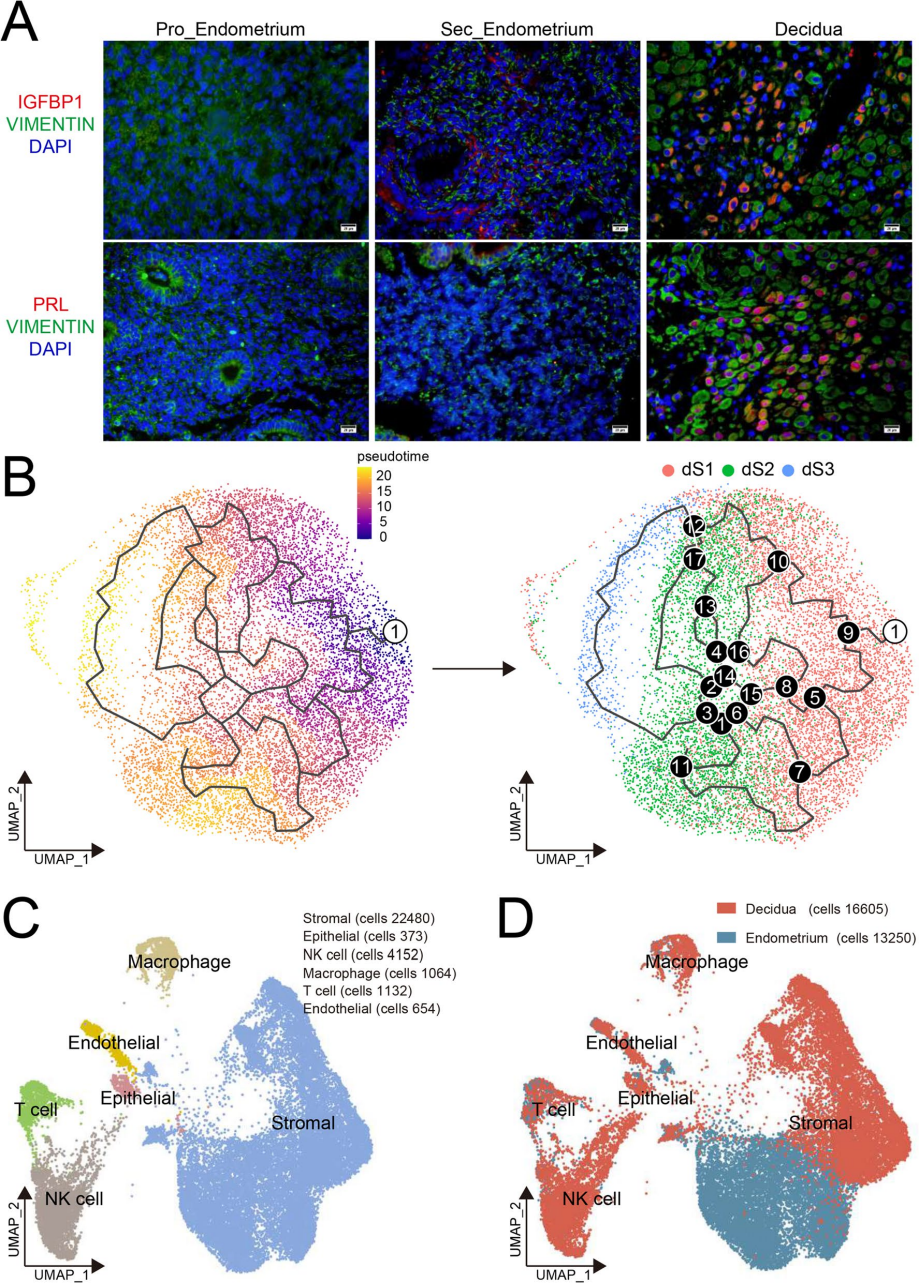
An atlas of human endometrium from non-pregnant and pregnant women. **A** Immunofluorescence staining of IGFBP1, PRL, and VIMENTIN in three different menstrual cycles of endometrial stroma. Scale bar, 20 μm. *n* = 6 each group. **B** Pseudotime analysis of decidual stromal cells in early pregnancy based on the previous research data (ArrayExpress, experiment codes: E-MTAB-6701; https://doi.org/10.1038/s41586-018-0698-6). **C** UMAP plots on single cells in endometrium from healthy controls at the WOI timing (Endometrium, *n* = 3) and normal decidua from early pregnancy (Decidua, *n* = 3), indicating major six cell types (Stromal, Epithelial, NK, Macrophage, T, Endothelial). **D** The subpopulation distribution of six main cell clusters of Endometrium and Decidua

To evaluate the heterogeneity and dynamic evolution characteristics of decidualized stromal cells, we analyzed the expression of marker molecules of decidualization in human endometrium during proliferative and secretory phases, and decidualized endometrium during early pregnancy. As shown, decidualization has begun at secretory endometrium during WOI time with a small number of IGFBP1^+^ and PRL^+^ cell population (Fig. 1A). Even in decidualized endometrium, some populations of stromal cell do not express IGFBP1 and PRL (Fig. 1A), suggesting that the heterogeneity of stromal cells is particularly significant in the process of decidualization. More interestingly, this hypothesis was verified in the pseudo-time analysis of decidual stromal cells in early pregnancy based on the previous report data (see “Materials and methods”) [28]. As shown, subset 1 of decidual stromal cells (dS1: IGF1^high^, IGFBP1^−^, PRL^−^) should be precursor cells of dS2 (IGF1^low^, IGFBP1^+^, PRL^−^) and dS3 (IGF1^−^, IGFBP1^high^, PRL^+^) (Fig. 1B).

To further determine the full repertoire of cell types and differentiation characteristics present in endometrium during decidualization, we isolated cells from endometrium from healthy controls at the WOI timing (endometrium, *n* = 3) and normal decidua from early pregnancy (decidua, *n* = 3) (see “Materials and methods”), and generated single-cell transcriptome libraries on the droplet-based 10X Genomics Chromium System (Additional file 1: Figure S1A). After computational quality control and integration of transcriptomes from both technologies, we obtained a total of 29, 855 cells (19 clusters) endometrial and decidual single-cell transcriptomes, and preformed graph-based clustering of uniform manifold approximation and projection (UMAP) (Fig. 1C, Additional file 1: Figure S1B) and used cluster-specific marker genes to annotate the clusters (Additional file 1: Figure S1C). Overall, all sequenced endometrial and decidual cells were assigned to six main classes of cells: fibroblast-like stromal cells (SC; expressing HOXA10, MME, COL1A1, and IGF1, 22,480 cells), epithelial cells (EPC; expressing KRT7, KRT8, KRT18, and EPCAM, 373 cells), NK cells (expressing PTPRC and NCAM1, 4152 cells), macrophages (expressing PTPRC and CD14, 1064 cells), T cells (expressing PTPRC and CD3D, 1132 cells), and endothelial cells (EC, expressing PECAM1 and ACKR1, 654 cells) (Fig. 1C, Additional file 1: Figure S1C).

Next, we displayed the characterization of cell contribution between endometrium and decidua from different donors (Fig. 1D, Additional file 1: Figure S1D and S1E). The subpopulation heterogeneity of six main cell clusters was obvious, especially SC (Fig. 1D). Accumulating evidence has indicated recruitment, enrichment, and redistribution of immune cells in decidua during early pregnancy [11–13]. As expected, more immune cell (IC) accumulation was observed in decidua compared to endometrium, especially NK cells and macrophages (Additional file 1: Figure S1D, S1E and Additional file 2: S2).

### IGF1^+^ stromal cells initiate endometrial decidualization

**Fig. 2:**
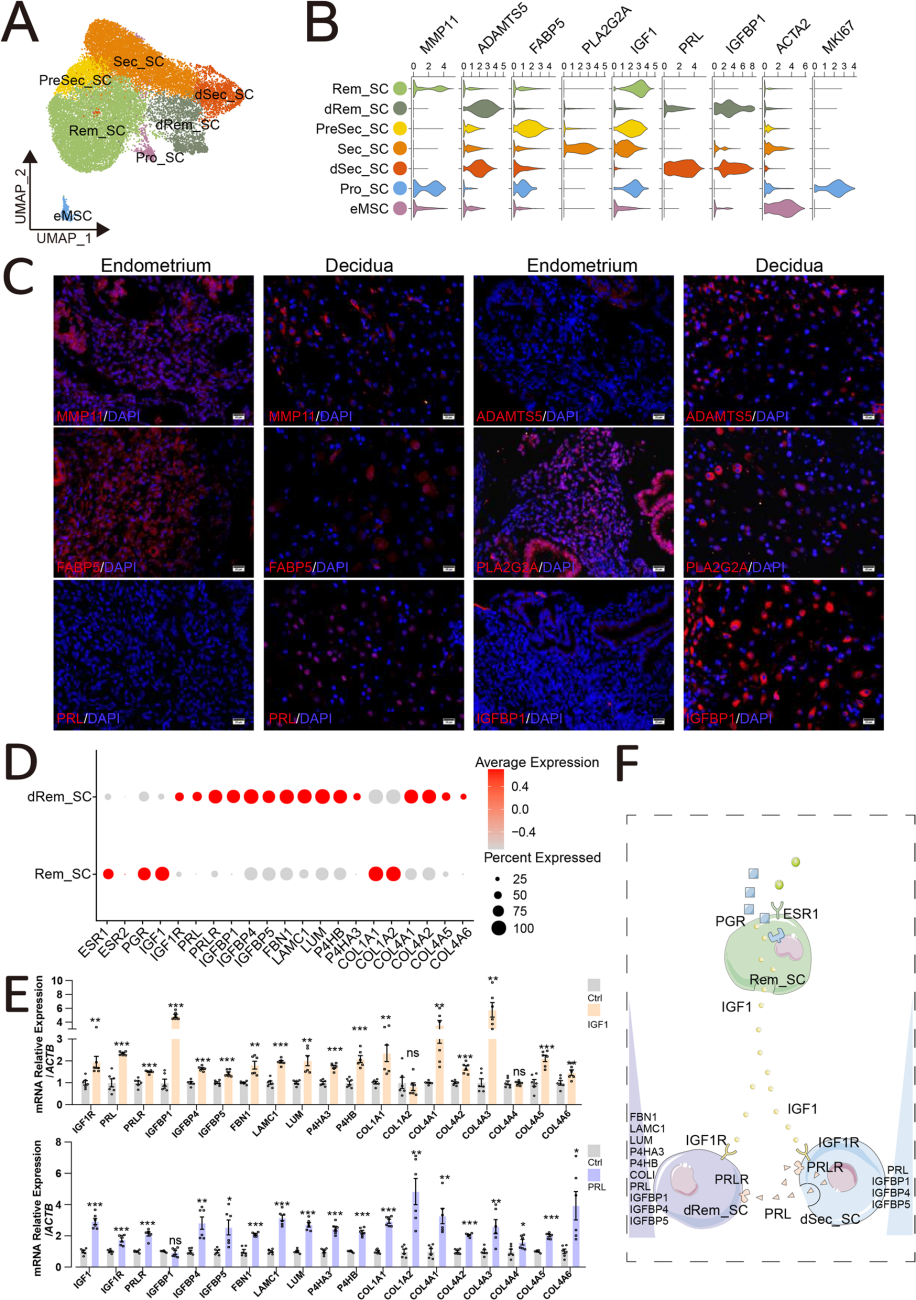
IGF1^+^ stromal cells initiate endometrial decidualization. **A** The cell cluster of stromal cells (22480 cells) was re-clustered into seven sub-clusters visualized by UMAP. **B** Violin plots of representative markers for seven main sub-clusters of stromal cells. **C** Immunofluorescence staining of MMP11, ADAMTS5, FABP5, PLA2G2A, PRL, and IGFBP1 in the endometrium (Endometrium, *n* = 3) and decidua (Decidua, *n* = 3). Scale bar, 20 μm. **D** Bubble diagram showing the average expression of selected genes for Rem_SC and dRem_SC. **E** Primary decidual stromal cells (DSCs) were treated with the rh-IGF1 (2 ng/mL), rh-PRL (0.1 ng/mL), or vehicle for 48 h. And then the mRNA expression levels of these genes in DSCs were measured by qRT-PCR (*n* = 6). Data were presented as mean ± SEM and analyzed by *t* test. (ns, no significance; *, *p* < 0.05; **, *p* < 0.01; ***, *p* < 0.001). **F** Diagram showed that IGF1^+^ stromal cells initiate endometrial decidualization under regulation of progesterone and estrogen

Since SC is the most abundant cell type in the endometrium and decidua, we initially explored endometrial and decidual SC and identified seven subset clusters of SC: IGF1^+^MMP11^+^DIO2^+^MKI67^−^PRL^−^IGFBP1^−^ Rem-SC (SC with high tissue remodeling property), IGF1^low^ADAMTS5^high^PRL^low^IGFBP1^+^ dRem-SC (decidualized SC with high tissue remodeling property), IGF1^+^FABP5^+^IGFBP3^+^PRL^−^IGFBP1^−^ PreSec-SC (SC with secretory ability), IGF1^low^PLA2G2A^+^IGFBP1^low^ Sec-SC (SC with high secretory ability), IGF1^−^PRL^high^IGFBP1^+^ADAMTS5^+^ dSec-SC, TOP2A^+^MKI67^+^ Pro-SC (SC with high proliferation), and ACTA2^+^RGS5^+^ endometrial mesenchymal stem cells (eMSCs) (Fig. 2A,B, Additional file 3: Figure S3A). In particular, a large number of genes involved in tissue remodeling (e.g., extracellular matrix (ECM) organization, cell adhesion, embryo implantation and placenta development), response to cAMP and cellular metabolic process, and cell cycle and proliferation were highly enriched in dRem-SC and Rem-SC, dSec-SC, and Sec-SC and PreSec-SC, and Pro-SC, respectively (Additional file 3: Figure S3B). Together with a small number of eMSCs, FABP5^+^PreSec-SC, MMP11^+^Rem-SC, and MKI67^+^Pro-SC constituted the main population of SC in endometrium. Differentially, the stroma of decidua was mainly based on dRem-SC, dSec-SC, and PLA2G2A^+^Sec-SC (Fig. 2C, Additional file 4: Figure S4A and S4B). This finding indicates a dramatical subpopulation heterogeneity of SC in endometrium and decidua. With the implantation of embryo and the maturation of endometrial decidualization, ECM remodeling, angiogenesis, autophagy levels, immune regulation, cell metabolism, and responses to oxidative stress of SC were increasingly active. In contrast, the proliferative potential of SC gradually decreased, as well as IGF1 (Additional file 4: Figure S4C and S4D).

Surprisingly, common decidualization-related genes, including leukemia inhibitory factor (LIF), Indian hedgehog (IHH), and dipeptidyl Peptidase 4 (DPP4) [30–32], were rarely expressed in SC of both endometrium and decidua (Additional file 5: Figure S5A). The classical marker genes (i.e., PRL and IGFBP1) of decidualization, and the enzymes (i.e., adenylate cyclase 1, ADCY1) for catalyzing the formation of cAMP (a strong inducer of decidualization) were highly expressed in decidualized SC, but were barely expressed in SC (PreSec-SC, Rem-SC and Pro-SC) of endometrium at the WOI (Fig. 2B, Additional file 5: S5A and S5B). More importantly, this process was accompanied by the advantage exchange between IGF1 and its receptor (IGF1R) levels. The data together suggest that the IGF1^+^ stromal cells located earlier in the endometrium should be involved in the initiation of decidualization.

To explore the initiating mechanism of decidualization, we first focused on two subpopulations of SC (Rem-SC and dRem-SC) with tissue remodeling (Additional file 6: Figure S6A), and constructed a new trajectory about Rem-SC and dRem-SC by a standard pseudo-time analysis (Additional file 6: Figure S6B and S6C). Notably, we observed a notable discontinuity between Rem-SC and dRem-SC. Endometrial Rem-SC was the starting point, which went through Rem-SC coexisted in endometrium and decidua, and the final endpoint was decidual dRem-SC (Additional file 6: Figure S6C). Further analysis showed that the expression of cell proliferation and differentiation (e.g., IGF1, and SFRP1, a modulator of Wnt signaling), apoptosis regulation (e.g., BCL2 Interacting Protein 3 Like, BNIP3L), and ECM disassembly (e.g., MMP16)-related genes were markedly reduced from the Rem-SC to dRem-SC in the trajectory (Fig. 2D, Additional file 7: Figure S7A and S7B). Importantly, gene expression about translation, positive regulation of cAMP catabolic process, cellular response to growth factor, and insulin receptor signaling pathway reached the peak in the last stage of Rem-SC and rapidly decreased in dRem-SC. In contrast, dRem-SC was characterized by high expression of ECM organization, embryo implantation, cell adhesion, angiogenesis, positive regulation of macrophage, and cytokine production-related genes (Additional file 7: Figure S7A and S7B).

Subsequently, the early expression of IGF1 in Rem-SC, PreSec-SC, and Pro-SC of endometrium at the WOI, like ESR1 and PGR, had attracted our attention (Fig. 2B–D). Interestingly, IGF1R was mainly expressed in dRem-SC with high levels of decidualized genes (e.g., PRL, PRLR, and IGFBP1), which was present in small amounts in endometrium (Fig. 2D). Further bioinformatics analysis displays IGF1 should be involved in the PGR and ESR1-triggered decidualization (Additional file 8: Figure S8A). As expected, medroxyprogesterone (MPA) plus estradiol (E2) upregulated IGF1 expression of human ESCs dramatically in vitro (Additional file 8: Figure S8B). More importantly, the expression of decidualization-related genes, especially PRL and IGFBP1, was quickly increased in human ESCs or primary DSCs stimulated by recombinant human IGF1 protein rather than MPA plus E2 (Fig. 2E, Additional file 8: Figure S8C). This process should be dependent on the strong effect on ADCY1 and ADCY3 for cAMP production, and high levels of PRL for further accelerating the decidualization (Fig. 2E, Additional file 8: Figure S8C and S8D). The data above indicate that IGF1^+^ SC possibly initiates endometrial decidualization under regulation of progesterone and estrogen (Fig. 2F).

### IL1B^+^ dSec-SC with active metabolism maintains decidual homeostasis

**Fig. 3:**
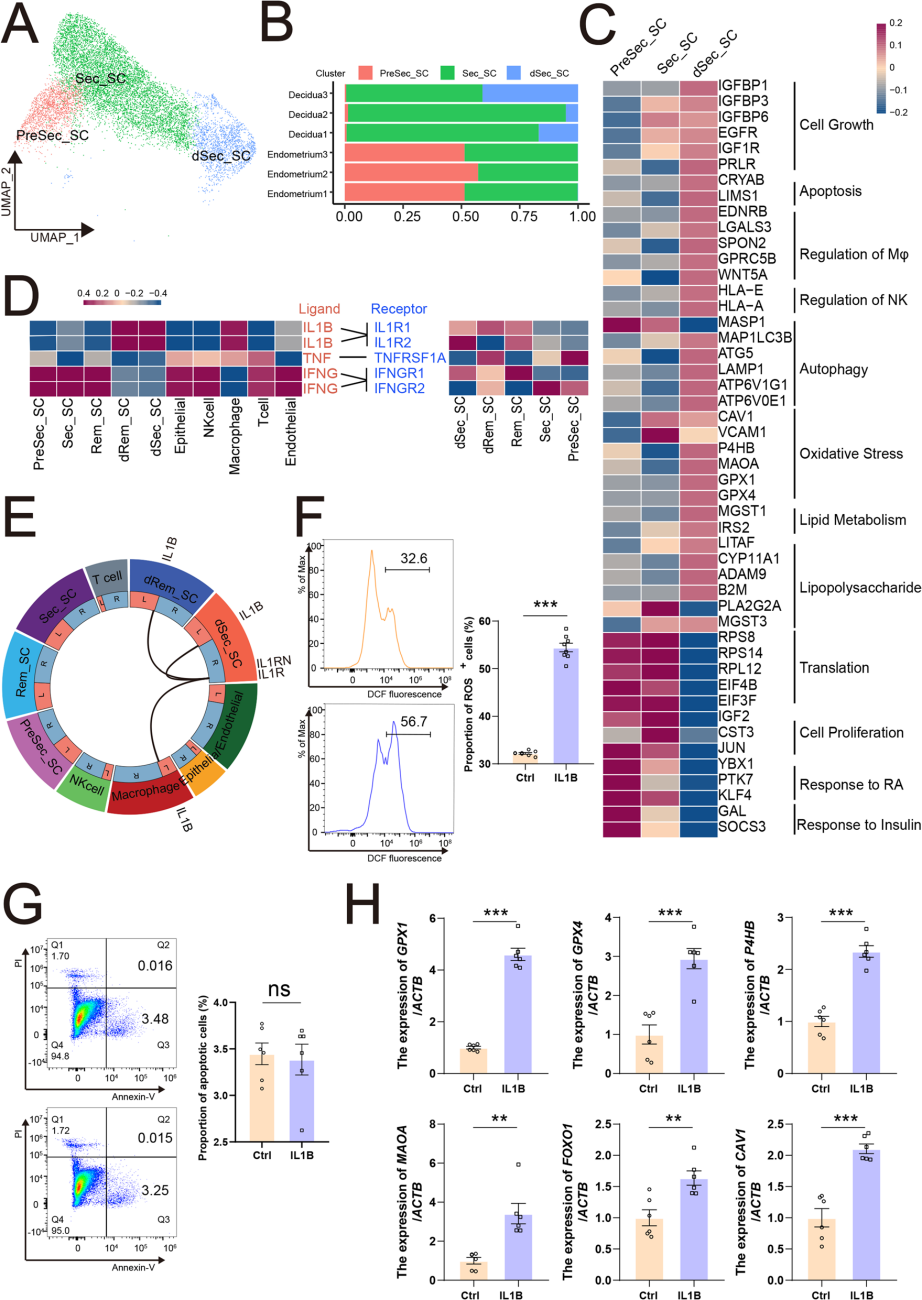
IL1B.^+^ dSec-SC with active metabolism maintains decidual homeostasis. **A** UMAP map of three sub-clusters (PreSec_SC, Sec_SC, dSec_SC) of stromal cells. **B** The proportion of PreSec_SC, Sec_SC, and dSec_SC from different samples. **C** Heat map showing relative expression (*z*-score) of selected genes for three sub-clusters of stromal cells. **D** Heat map showing selected significant ligand–receptor interactions (*P* value < 0.05, permutation test, see “Methods”) between endometrial cells (left) and five sub-clusters of stromal cells (right). Assays were carried out at the mRNA level, but are extrapolated to protein interactions. **E** Circos plot of interaction network between stromal cells and other endometrial cells. **F** ROS levels in DSCs were detected by a 2,7‑dichlorofluorescin diacetate assay (*n* = 6). **G** The apoptosis of DSCs in control group and rh-IL1B protein (50 ng/mL) group was detected by the flow cytometry assay (*n* = 6). **H** DSCs were treated with the rh-IL1B (50 ng/mL) or vehicle for 48 h. And then the mRNA expression levels of these genes in DSCs were measured by qRT-PCR (*n* = 6). Data were presented as mean ± SEM and analyzed by *t* test. (ns, no significance; *, *p* < 0.05; **, *p* < 0.01; ***, *p* < 0.001)

Decidual transformation is associated with the accumulation of glycogen and lipid droplets in the expanding cytoplasm, increased activities of endocytosis/exocytosis, and protein biosynthesis [1, 11, 33–35]. In other three subpopulations of SC, PreSec-SC and Sec-SC had high levels of protein translation-related genes (RPL12 and EIF3F), and dSec-SC, only found in the decidua, were rich in genes associated with cell growth, phagosome, and multiple metabolic pathways (e.g., oxidative phosphorylation, glutathione metabolism, glycolysis, gluconeogenesis, and lipid metabolism) (Fig. 3A–C, Additional file 9: S9A and S9B). Additionally, dSec-SC with a high level of PRL and IGFBP1 were involved in regulating macrophage chemotaxis and cytokine production, and NK cell-mediated cytotoxicity, and characterized by high levels of autophagy (e.g., MAP1LC3B, ATG5), cellular response to hypoxia (e.g., FOXO1, CAV1), oxidation–reduction process (e.g., P4HB, MAOA), response to reactive oxygen species (ROS) (e.g., GPX1, GPX4), responses to lipopolysaccharide (e.g., LITAF, CYP11A1, DADM9, and B2M), and glutathione derivative biosynthetic process (e.g., MGST1, MGST3)-related genes (Fig. 3C).

This process of blastocyst invasion of the maternal uterine endometrium has been reported to depend on an evolutionarily conserved inflammatory response including IL1B, INFG, TNF, and IL6 [36–38]. As shown, IL1B was mainly derived from dSec-SC in decidua, which also highly expressed IL1B and receptors (IL1R1 and IL1R2) (Fig. 3D and Additional file 9: S9C). The potential interaction of IL1B and receptors between these five clusters of SCs and other cells (EPC, NK cells, macrophages, T cells, and EC) in decidua were predicted (Fig. 3E). To confirm the role of IL1B in SC, primary decidual stromal cells were treated with recombinant IL1B protein in vitro. IL1B significantly upregulated the levels of ROS in DSC, along with the increases of various anti-oxidative stress genes (e.g., GPX1), but no influenced cell apoptosis (Fig. 3F–H). Collectively, these findings suggest that dSec-SC with activate metabolism, on the one hand, provides essential nutrients and immunotolerance environment for blastocyst implantation and placental development and, on the other hand, maintains decidual homeostasis by powerful phagocytosis, and strong protection against oxidative stress caused by various factors (e.g., inflammatory response, hypoxia, and lipid metabolism).

### IL1B^+^ dSec-SC triggers the apoptosis of epithelial cells during decidualization

**Fig. 4:**
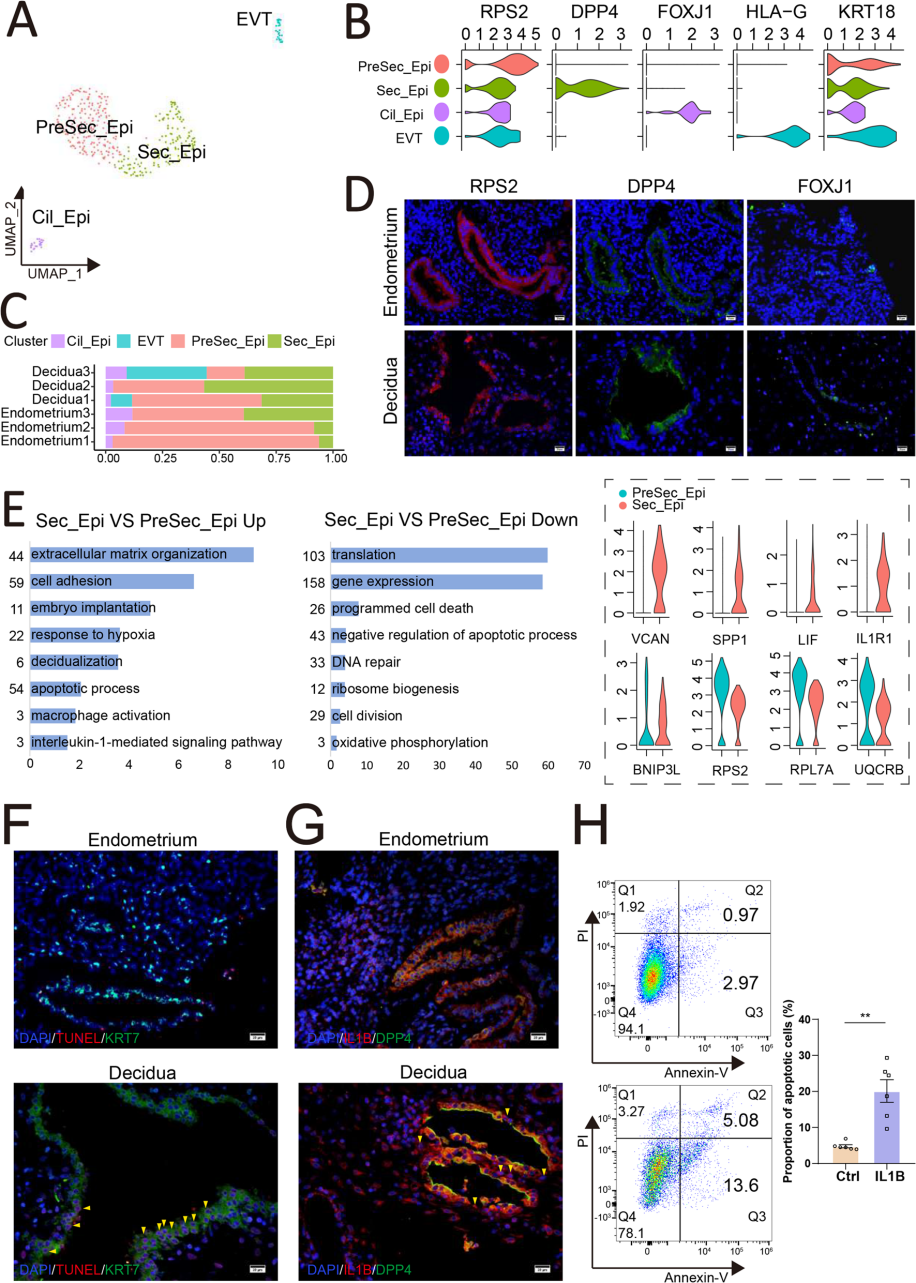
IL1B triggers the apoptosis of epithelial cells during decidualization. **A** UMAP map of four sub-clusters (PreSec_Epi, Sec_Epi, Cil_Epi, and EVT) of epithelial cells. **B** Violin plots of representative markers four sub-clusters of epithelial cells. **C** The proportion of four sub-clusters of epithelial cells from different samples. **D** Immunofluorescence staining of RPS2, DPP4, and FOXJ1 in the endometrium of Endometrium and Decidua group (*n* = 3 for each group). Scale bar, 20 μm. **E** Significant gene markers for each cluster were selected to perform GO analysis. GO terms with *P* < 0.05 are shown. Gene number of each GO term is listed on the left. *P* value is shown as − log 10 (*P* value). Violin plots of relative genes are shown on the right. **F** TUNEL assays images from representative experiments in the endometrium of Endometrium and Decidua (*n* = 3). The red fluorescence indicates apoptosis (arrows). Scale bar, 20 μm. **G** Immunofluorescence staining of IL1B and DPP4 in the endometrium of Endometrium and Decidua (*n* = 3). The red fluorescence indicates IL1B^+^DPP4.^−^ cells (arrows). Scale bar, 20 μm. **H** The apoptosis of human endometrial epithelial cells (hEECs) in THE control group and rh-IL1B protein group (50 ng/mL) was detected by the flow cytometry assay (*n* = 6).( **, *p* < 0.01)

As the site of blastocyst adhesion, the luminal epithelium is perceived as the crucial site for uterine receptivity [39]. Markers that distinguish the different endometrial and decidual EPC populations identify 3 clusters: KRT18^+^FOXJ1^+^RPS2^+^ Ciliated epithelial cell (Cil-Epi), KRT18^+^FOXJ1^−^RPS2^high^DPP4^−^ glandular epithelial cell (PreSec-Epi), and KRT18^+^FOXJ1^−^DPP4^+^RPS2^+^ glandular epithelial cell (Sec-Epi, with high decidualization and immune regulation abilities) (Fig. 4A, B, and Additional file 10: S10A-C). Of note, KRT18^+^HLA-G^+^ extravillous trophoblasts (EVT) are observed in two samples of decidua (Fig. 4B, C). PreSec-Epi predominate in endometrium at the WOI, but there is more Sec-Epi in decidua (Fig. 4C, D), which was characterized by high levels of decidualized genes (e.g., IGFBP1, LIF, DPP4, CXCL14) (Additional file 10: Figure S10B and S10C).

In humans and mice, endometrial decidualization is characterized by apoptosis in the glands [40–43]. According to functional enrichment analysis, the genes involved in ECM organization, cell adhesion, embryo implantation, regulation of cell migration, aging, apoptotic process, macrophage activation, angiogenesis, and autophagy were highly enriched in Sec-Epi compared to PreSec-Epi (Fig. 4E). The results of TUNEL assay confirmed that there was a higher level of apoptosis of glandular epithelial cell in decidua than that in endometrium (Fig. 4F). Interestingly, we observed DPP4^+^ Sec-Epi in the lumen of decidua is discontinuous and interspersed with IL1B^+^ dSec-SC and PLA2G2A^+^ Sec-SC using the immunofluorescence staining (Fig. 4G and Additional file 11: S11A). To investigate the potential role of IL1B produced by dSec-SC in epithelial cells (Additional file 11: Figure S11B), human endometrial epithelial cell (EEC) line was treated with or without IL1B for 24 h. As shown, IL1B induced the apoptosis of EECs markedly in vitro (Fig. 4H). These data indicate that IL1B^+^ dSec-SC is involved in the apoptosis of epithelial cells during decidualization.

### AREG^+^ NK cell accelerates decidualization by interacting with IGF1^+^ SC

Although the potential mechanisms are largely unknown, there is evidence for a large number of immune cell infiltration in the endometrium at the WOI, and enrichment and redistribution in decidua at the maternal–fetal interface, including NK, macrophage, T and dendritic cell (DC) [11–13, 44]. Here we observed a very rich population of immune cell, such as CD45^+^CD3^−^CD56^+^ NK cell, CD45^+^CD14^+^ macrophage, CD45^+^CD3^+^CD8^−^CD4^+^ T cell, and CD45^+^CD3^+^CD4^−^CD8^+^T cell in decidua compared with endometrium (Additional file 12: Figure S12 and Additional file 13: S13).

As an abundant leukocyte population in the non-pregnant endometrium and pregnant decidua, macrophages are considered to play a central role in the establishment and maintenance of normal pregnancy [45]. However, the change and characteristic of macrophages during decidualization remain unclear. Here, we identified seven main subsets from total macrophage/DC cluster (Additional file 14: Figure S14A and S14B). M1 cells highly expressed folate receptor beta (FOLR2), M2 cells highly expressed secreted phosphoprotein 1 (SPP1, also known as OPN) (Additional file 14: Figure S14B and S14C), M3 cells expressed CX3CR1 (an important chemokine receptor for macrophage migration and recruitment), pro-macrophages expressed MKI67, monocytes expressed S100 calcium-binding protein A9 (S100A9), cDC1 cells expressed c-type lectin domain containing 9A (CLEC9A) but not CD1C, and cDC2 cells expressed CD1C. Additionally, the closed crosstalk between four subpopulations (M1, M2, M3, and pro-macrophages) of macrophages and other cells was predicted in endometrium and decidua, including the regulation of focal adhesion, ECM receptor interaction, cytokine-cytokine interaction, and NK cell-mediated cytotoxicity (Additional file 15: Figure S15A). Further analysis showed that M1 cluster displays a M2-like phenotype (high levels of MRC1 and CD209) and high levels of IGF1 and PDGFB, suggesting that M1 cluster should be involved in tissue remodeling, decidualization, and immune tolerance (Additional file 15: Figure S15B). The chemokine genes (e.g., CCL2, CCL3, CCL4), SPP1, VEGFA, and VEGFB were enriched in M2 cluster, indicating that M2 cluster participates in the recruitment of immune cells and angiogenesis. M3 cluster had high levels of CD86, IFNGR1, HLA-DQA2, HLA-DPB1, and PLD4, contributing to the immune response and antigen presentation. Compared to endometrium, there are more macrophages in decidua, especially M1 cluster (Additional file 15: Figure S15C). FOLR2 has been reported to regulate folate uptake and absorption in tissue-resident M2-like macrophages, which should be dependent on the activin A (encoded by INHBA) [46]. Activins are important autocrine and paracrine regulators of endometrial decidualization and the priming of endometrium for implantation [42]. The expression of INHBA, INHBB, and INHA subunits are increased in decidualized stromal cells at the onset of decidualization [47]. More importantly, INHBA and INHA were expressed in IGF1^+^ Rem-SC, dRem-SC, and dSec-SC, respectively (Additional file 15: Figure S15D). These data above suggest that enrichment of FOLR2^+^IGF1^+^M1 macrophage cluster with a M2-like phenotype possibly induced by SC-derived activin A results in maternal–fetal immune tolerance, tissue remodeling, and decidualization during early pregnancy.

**Fig. 5:**
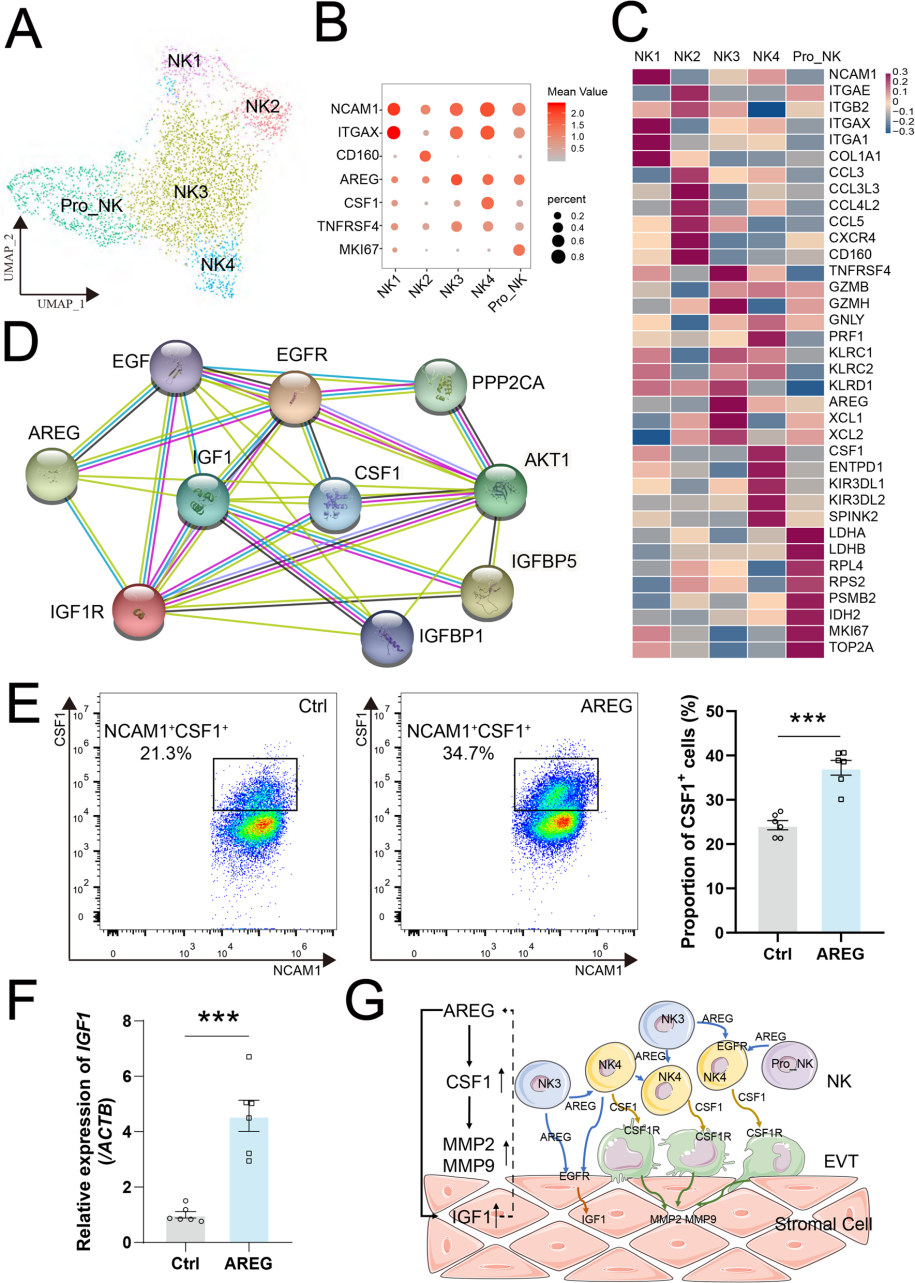
AREG^+^ NK cell accelerates decidualization by interacting with IGF1^+^ stromal cells. **A** UMAP map of five sub-clusters of NK cells. **B** Bubble diagram showing the average expression of marker genes for five sub-clusters of NK cells. **C** Heat map showing relative expression (*z*-score) of selected genes for five sub-clusters of NK cells. **D** The PPI network of AREG, IGF1, CSF1, and other related genes. **E** Primary decidual NK cells were treated with rh-AREG (100 ng/mL) or vehicle for 48 h. The proportion of CSF1^+^ NK cells were analyzed by flow cytometry (*n* = 6). Data were presented as mean ± SEM and analyzed by *t* test. (ns, no significance, ***, *p* < 0.001). **F** hESCs were treated with the rh-AREG (100 ng/mL) or vehicle for 48 h. And then the mRNA expression levels of *IGF1* in hESCs were measured by qRT-PCR (*n* = 6). Data were presented as mean ± SEM and analyzed by *t* test. (***, *p* < 0.001). **G** Schematic cell interactions in endometrium between AREG^+^ NK cells and IGF1^+^ stromal cells during decidualization

NK cells are the most distinguishable lymphocytes during the first trimester of pregnancy, constituting 50 ~ 70% of all leukocytes in human decidua [27, 28]. Here, we identified 5 clusters of NK cells in endometrium and decidua (Fig. 5A, and Additional file 16: Figure S16), including ITGA1^+^NCAM1^high^ITGAE^−^TNFRSF4^−^ NK1, NCAM1^+^CD160^+^CXCR4^+^ NK2, ITGA1^+^NCAM1^high^TNFRSF4^+^AREG^+^ NK3, ITGA1^+^NCAM1^high^TNFRSF4^+^AREG^+^CSF1^+^ NK4 cells and MKI67^+^TOP2A^+^ pro-NK cells (Fig. 5B, C). Among these, pro-NK cells with high proliferation potential are metabolically active (e.g., pyruvate metabolism, carbon metabolism, glycolysis / gluconeogenesis, and oxidative phosphorylation) (Additional file 17: Figure S17). NK2 cells highly expressed various chemokines (e.g., CCL5, CCL3L3, CCL4L2, and CCL3), CXCR4 and CD160, but had a low cytotoxicity, which were mainly located in endometrium at the WOI (Fig. 5C, Additional file 18: Figure S18A and S18B). Therefore, NK2 cells should contribute to immune recruitment and infiltration. NK1 cells with high levels of ITGAX, NCAM1, and COL1A1 are involved in ECM receptor interaction with other cells, such as SC and EVT (Fig. 5C, Additional file 18: Figure S18C and S18D). NK3 cells with high levels of GZMB and GZMH, KLRC1, and AREG, and NK4 cells with high levels of KIR genes (KIR3DL1 and KIR3DL2), granule protein-coded genes (GNLY and GZMB), AREG and CSF1, were mainly enriched in decidua (Fig. 5C and Additional file 18: S18A). Among these, NK4 and pro-NK, NK3, and NK2 are consistent with the characteristics of dNK1 and dNKp, dNK2, and dNK3 reported by Vento-Tormo and colleagues using scRNA-seq [28], respectively. AREG, also known as amphiregulin, has been reported to regulate the production of GM-CSF, co-operate with IGF1 for regulation of cell growth, and directly stimulate trophoblast invasion [48–50]. CSF1 contributes to trophoblast invasion (Additional file 18: Figure S18E and S18F), the recruitment, and M2 differentiation of macrophage [51, 52]. Additionally, AREG is predicted to regulate the IGF1 and CSF1 by the protein–protein interaction (PPI) network analysis (Fig. 5D). The data above and the results in vitro (Fig. 5E,F) suggest that AREG^+^ NK3 cells should promote the differentiation of CSF1^+^ NK4 cells and IGF1^+^ SC, and further contributing to the decidualization and embryo implantation. Importantly, cAMP-dependent regulation of ovulatory response genes (e.g., AREG) has been reported to be amplified by IGF1 [53], suggesting that there is a close dialog between AREG^+^ NK cells and IGF1^+^ SC during decidualization (Fig. 5G).

### EVT promotes decidualization by multiply pathways

**Fig. 6:**
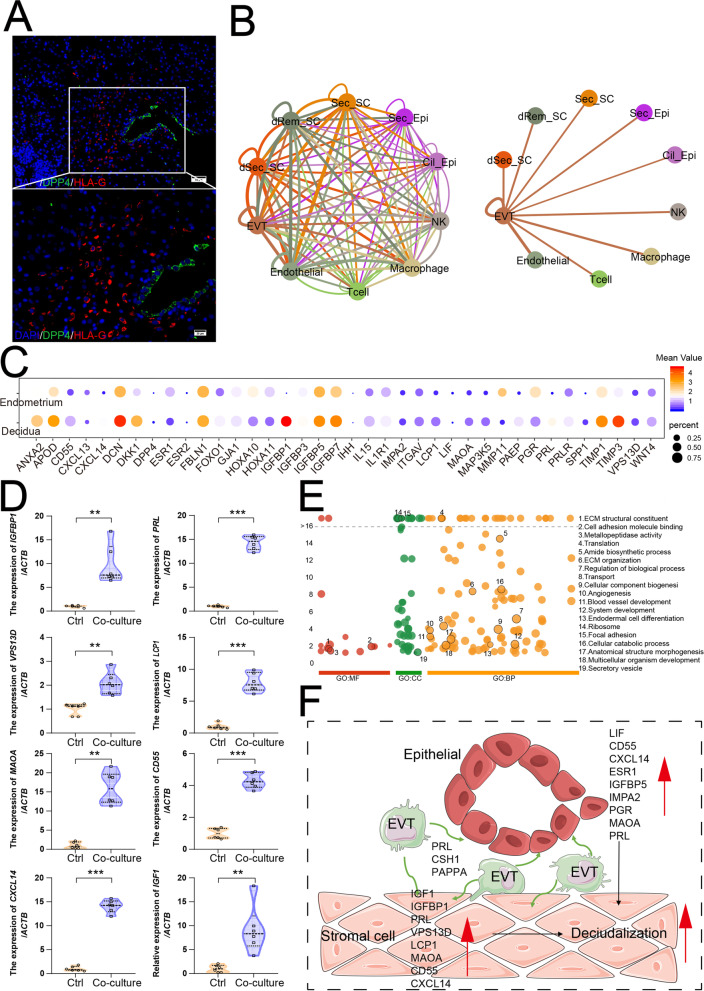
EVT promotes decidualization. **A** Immunofluorescence staining of HLA-G and DPP4 in the decidua (*n* = 3). Scale bar, 20 μm (upper), 50 μm (below). **B** Intercellular communication analysis among all cell types in endometrium. Line color indicates ligands broadcast by the cell population of the same color. Lines connect to cell populations where cognate receptors are expressed. Line thickness is proportional to the number of ligands where cognate receptors are present in the recipient cell population. Loops indicate autocrine circuits. Map quantifies potential communication but does not account for anatomic position or boundaries of cell populations. Detailed view of ligands broadcast by extravillous trophoblasts (EVT) and other cell populations expressing cognate receptors primed to receive a signal (right). **C** Bubble diagram showing the average expression of decidualization-related genes of stromal cells in the Endometrium and Decidua group. **D** hESCs (5 × 10^5^ cells) were co-cultured with or without HTR8/SVneo (5 × 10^5^ cells) for 24 h. And then the mRNA expression levels of these genes in hESCs were measured by qRT-PCR (*n* = 6). Data were presented as mean ± SEM and analyzed by *t* test. (**, *p* < 0.01; ***, *p* < 0.001). **E** Manhattan plot indicating the Gene Ontology (GO) enrichment, and the most important GO terms of top 100 genes of EVT. Manhattan plot was drawn by online tool—g:Profile (https://biit.cs.ut.ee/gprofiler/). **F** Pattern diagram of EVT promoting decidualization

The embryonic EVT invade the decidua and uterine myometrium, infiltrating the uterine vessels and glands to direct nutrients to the developing fetus. Therefore, HLA-G^+^ EVT direct contact with maternal-derived decidual cells, including SC, EPC, IC, and EC (Fig. 6A, B, and Additional file 19: Figure S19A). With embryo implantation, the expression of some classic decidualization-related genes in SC (e.g., PRL, IGFBP1, CXCL14, MAP3K5) and EPC (e.g., LIF, DPP4, IGFBP1, CXCL14) in decidua were upregulated rapidly (Fig. 6C and Additional file 19: Figure S19B). Further analysis confirmed co-culture with human trophoblast HTR8/SVneo cell line led to the elevation of these decidualization-related genes in human ESC and EEC cells in vitro (Fig. 6D and Additional file 19: S19C), indicating that EVT promote the decidualization. The top 100 genes (e.g., CSH1, FN1, NOTUM, SERPINE2, QSOX1, ISM2, FLT1, HSPG2, FSTL3, HLA-G, PAPPA2, PAPPA, COL4A1, CDKN1C, HTRA4, FSTL1, COL4A2, CTSL, HPGD, PTPRF) in EVT were mainly associated with ECM remodeling, cell adhesion, angiogenesis and blood vessel development, contributing to embryo implantation, embryonic and placental development, and immune regulation (Fig. 6E and Additional file 2: Figure S20). As a member of the somatotropin/prolactin family of hormones, chorionic somatomammotropin hormone 1 (CSH1) promotes fetal growth and metabolism by activating PRLR. More importantly, CSH1 and PRL are enriched in EVT, and predicted to be associated with the IGF1, CSF1, and AREG (Additional file 21: Figure S21A and S21B). Additionally, pregnancy-associated plasma protein A (PAPPA) and PAPPA2 encode secreted metalloproteinases which cleave IGFBPs, and are thought to be local regulators of IGF bioavailability [54]. Therefore, the stimulatory effect of EVT on decidualization should be dependent on the regulation of CSH1, PRL, and PAPPAs (Fig. 6F).

### The aberrant ratio of IGF1^+^SC to IGF1R^+^SC is observed in repeated implantation failure patients

**Fig. 7:**
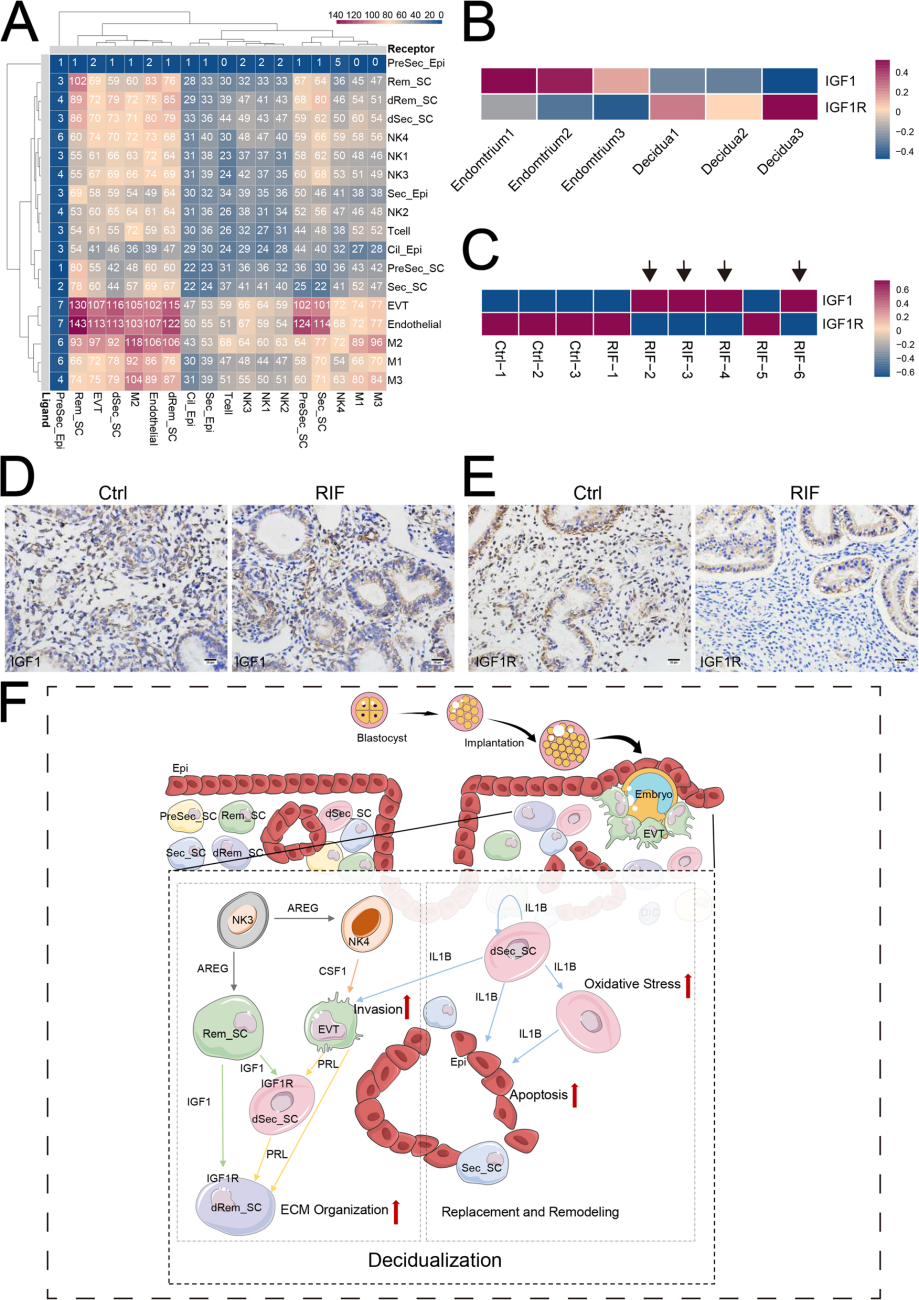
The aberrant ratio of IGF1^+^SC to IGF1R^+^SC is observed in repeated implantation failure patients. **A** Cell phone heatmap showed the interaction of cells. The number indicates the number of interactions. **B** Heat map showing relative expression (*z*-score) of IGF1 and IGF1R in the total Rem_SC (Rem_SC and dRem_SC) from Endometrium and Decidua. **C** Heat map showing relative expression (*z*-score) of IGF1 and IGF1R in the stroma cells from Ctrl and RIF. **D, E** The expression of IGF1 and IGF1R in the endometrial stroma from controls and RIF patients at the WOI time were analyzed by immunohistochemistry (*n* = 3 for each group). Scale bar, 20 μm. **F** General diagram of decidualization process. During the time of WOI, the heterogeneity of stromal cells determines different division of labor. More specifically, IGF1^+^ stromal cells (Rem) start endometrial decidualization (mainly IGF1R^+^ stromal cells, i.e., dRem and dSec) by crosstalking with EVT and immune cells, including AREG^+^ NK cells; IL1B^+^ stromal cells (dSec) participate in the establishment and maintenance of decidua hemostasis

To systematically study the interactions of endometrial and decidual cells, we developed a repository of ligand-receptor interacting pairs, representing a complex regulatory network by intercellular communication analysis (Fig. 7A and Additional file 22: Figure S22). Of note, there was the most intense crosstalk between SC (Rem-SC and dRem-SC) and EVT. EVT with powerful cell adhesion and epithelial cells should stimulate decidualization and embryo implantation by PRL/PRLR, pleiotrophin (PTN)/PLXNB2, LIF/LIFR, and or INHBB/ACVR, respectively (Additional file 22: Figure S22). In addition to decidualization development, NK cells and macrophage contribute to stromal cell homeostasis by FASLG/FAS, GAS6/AXL, GAS6/MERTK, PROS1/AXL, AREG/EGFR, TGFB/EGFR, and PDGFs/PDGFRs (Additional file 22: Figure S22). In turn, SC facilitates embryo adhesion, implantation and development, lymphocyte recruitment, immune tolerance, and angiogenesis by FN/integrins, CCL8/CCR1, CXCL12/CXCR4, IL15/IL15R, TGFB1/TGFBR, and VEGFs/VEGFRs, especially dSec_SC and dRem_SC (Additional file 23: Figure S23).

As noted above, IGF1^+^ SC was considered to start decidualization by interacting with IGF1R^+^ SC. More importantly, the conversion of IGF1^+^SC to IGF1R^+^SC in stromal cells was hampered in some cases of unexplained RIF patients (Fig. 7B, C), which was also confirmed by immunohistochemical staining (Fig. 7D). These data together suggest the subset imbalance between IGF1^+^SC and IGF1R^+^SC is involved in the pathogenesis of RIF possibly by decidualization deficiency.

## Discussion

The infertility rate ranges between 9 and 18% of the general population worldwide [55]. Indeed, the normal monthly fecundity rate is below 30% in women of childbearing age due to embryo loss before implantation [1]. It is well established in humans that decidualization is characterized by a series of morphological, genetic, metabolic, biochemical, and immune changes occurring within the endometrial stroma, and our knowledge of this relevant biological process has been evolving [1, 56]. Growing evidence supports the concept that an improper decidualization response even before the blastocyst arrives can be a determinant of pregnancy outcomes, not only in early (e.g., implantation failure, miscarriage) but also in advanced gestation [1–6]. Therefore, exploring detailed cellular communication and spatiotemporal regulatory mechanisms during decidualization are particularly critical for the identification of decidualization degree and treatment of decidualization deficiency-related diseases. In this study, we have used scRNA-seq and observed obvious heterogeneity of stromal cells in human endometrium and decidua. More importantly, we found a unique subpopulation of IGF1^+^ stromal cell probably participates in triggering decidualization of IGF1R^+^ stromal cells; IL1B^+^ stromal cells with high oxidative stress that intrude into epithelial cells can induce epithelial cell apoptosis and promote the invasion implantation of embryonic trophoblasts; and enriched AREG^+^ NK cells accelerate decidualization by upregulating IGF1 of stromal cells, and trophoblast invasion by inducing CSF1^+^ NK cells. After trophoblast implantation, it further accelerates decidualization by multiple pathways, such as PRL, CSH1, and PAPPA. As depicted in Fig. 7E, this series of events together constitute decidualization of human endometrium.

Here, we defined five major stromal cell population (dRem-SC and Rem-SC, dSec-SC, and Sec-SC and PreSec-SC, Pro-SC, and eMSCs) in endometrium and decidua. Rem-SC, PreSec-SC, and Pro-SC are mainly located in endometrium; however, decidual stroma is mainly composed of dRem-SC, dSec-SC, and Sec-SC. Of note, IGF1 was mainly expressed in three subsets of stromal cells in endometrium, especially Rem-SC. In contrast, IGF1R is predominantly expressed in decidualized stromal cells, especially dRem-SC. IGF1 plays fundamental roles during development, maturation, and aging in human. More importantly, we found that IGF1 under positive regulation by estrogen and progesterone can initiate decidualization of stromal cell quickly in vitro, even stronger and earlier than MPA plus estrogen. The upregulation of ADCY1 and ADCY3 is bound to contribute to this process. Additionally, PRL induced by IGF1 further promotes the decidualization, resulting to the continuous maturation of decidualization in a cascade manner.

We also observed dSec-SC is characterized by multiple activated metabolic pathways, including glutathione metabolism, glycolysis, gluconeogenesis, and lipid metabolism. Further analysis reinforces our hypothesis suggesting that dSec-SC is conducive to the decidual homeostasis for adapting to the stormy and complex stress microenvironment during blastocyst implantation, and formation of immune and metabolic microenvironment for supporting blastocyst implantation and placental development. More than that, this subset of IL1B^+^ dSec-SC broke into the territory of DPP4^+^ Sec-Epi mainly located in decidua and replaces and remodels glands by triggering the apoptosis of epithelial cells. This observation deepens our understanding of the mechanism of epithelial cell degeneration in the process of endometrial decidualization. In view of the important role of IL1B in decidualization [57], the role of theIL1B^+^ dSec-SC in the development of decidualization cannot be ignored yet.

Decidualized stromal cells have reported to accelerate the residence and enrichment of NK cells in decidua, and depletion or absence of NK cells resulted in adverse outcomes, including reduced number of implanted embryos, and increased embryo loss [13], further emphasizing the importance of NK cells in the establishment and maintenance of normal pregnancy [58]. Here, we defined five subsets of NK cells in isolated endometrial from non-pregnant women and decidual cells from women in early pregnancy, including NK1, NK2, NK3, NK4, and Pro-NK cells. Of note, the dominant NK cell population in decidua is highly expressed AREG, especially NK3. Further analysis showed ARGE upregulated the expression of IGF1 in stromal cell and the percentage of CSF-1^+^ NK cells, contributing to decidualization development, trophoblast invasion, and macrophage regulation. Interestingly, we observed a FOLR2^+^IGF1^+^M1 macrophage cluster with a M2-like phenotype was enriched in decidua, possibly contributing to accelerating decidualization. Additionally, endometrial angiogenesis is very important for decidualization [59, 60]. Here, we found SC and immune cells (e.g., macrophage and NK cells) should be involved in angiogenesis by several cytokines, which needs to be further researched.

As the only embryo derived cells in direct contact with maternal decidual cells, EVT have been reported to play an important role in the progress of decidualization [15, 16]. However, the exact mechanisms are largely unknown. Here we observed that enriched genes in EVT were involved in the regulation of ECM remodeling, cell adhesion, vascularization, and blood vessel development. Even more concerning, hormones and proteins (e.g., CSH1, PRL and PAPPA), produced by EVT, contribute to decidualization development, and this regulation should be dependent on the interaction of EVT and IGF1^+^ SC. Cellular communication analysis also indicates that stromal cells facilitate embryo adhesion, implantation and development, lymphocyte recruitment, immune tolerance, and angiogenesis by several ligands/receptors. While assisting decidualization, EVT and decidual immune cells (NK, macrophage and T cells) can also promote embryo implantation and maternal–fetal immune tolerance through producing TGFB [61].

## Conclusions

Therefore, benign and effective interactions between mother and fetus in decidualization are undoubtedly essential for receiving embryo implantation. Our previous reports showed the expression of IGF1 in stromal cells in secretory endometrium was higher than that in proliferative endometrium [62]. During the time of WOI, the heterogeneity of stromal cells determines different division of labor. More specifically, IGF1^+^ stromal cells (Rem) start endometrial decidualization (mainly IGF1R^+^ stromal cells, i.e., dRem and dSec) by cross talking with EVT and immune cells, including AREG^+^ NK cells; IL1B^+^ stromal cells (dSec) participate in the establishment and maintenance of decidua hemostasis. This precise division and cooperation of stromal cells jointly initiate and promote decidualization and create a unique endocrine, immune and metabolic microenvironment conducive to embryo implantation and development. More importantly, the advantage conversion of IGF1^+^ stromal cells to IGF1R^+^ stromal cells are beneficial to evaluation of endometrial receptivity and the opening of WOI. Our observation suggests that the aberrant ratio conversion of IGF1^+^ stromal cells to IGF1R^+^ stromal cells is closely associated with unexplained RIF possibly by decidualization deficiency, which echoed the decreased ratio of embryo implantation in uterine IGF1R knockout female mice [63]. Therefore, the comprehensive and detailed analysis of the subsets of endometrial stromal cells will help us to explore new diagnostic and therapeutic strategies for unexplained RIF and decidualization disorder-related diseases.

## Materials and methods

### Patients and sample collection

The protocol for this study was approved by the Human Research Ethics Committees of Obstetrics and Gynecology Hospital of Fudan University, and written informed consent was obtained from all participants. For scRNA-seq, the endometrium of the Endometrium group (*n* = 3, age range, 29–35 years) was defined as previous fertility history. The time of endometrial biopsy was 5 days after ovulation (ultrasonic observation, equate to LH + 7, the WOI time) in a natural cycle. Decidual tissues from normal pregnant patient group (*n* = 3) were collected from normal women in the first trimester of pregnancy for selective termination (age, 25–35 years old; gestational age, 7–9 weeks). Repeated implantation failure (RIF) patient group (*n* = 6, age range, 32–35 years) defined as unsuccessful implantation following transfer for at least 6 morphologically good-quality embryos totally in three of more embryo transfer cycles, was collected.

For immunofluorescence, immunohistochemistry, flow cytometry analysis, and in vitro trials, the endometrium from normal proliferative or secretory phase (*n* = 15, age range, 25–33 years), RIF (*n* = 6, age range, 32–35 years) or decidua from the first trimester of normal pregnancy (*n* = 21, age range, 25–34 years) was obtained. All donors had a regular menstrual cycle (6–7 days every 28–30 days). Women with the following conditions were excluded from tissue collection: genetic abnormalities, recent contraception (intrauterine device usage in past 3 months; hormonal contraceptives in past 3 months), endocrine metabolic abnormalities (i.e., polycystic ovary syndrome, diabetes, insulin resistance, hypothyroidism), severe adenomyosis or endometriosis, severe hydrosalpinx, moderate to severe intrauterine adhesions, uterine malformations, recurrent miscarriage, thrombosis and autoimmune diseases, and BMI > 30.

### Single-cell dissociation

The endometrial and decidual tissues were washed with ice-cold PBS to wash away the remaining blood. And then, the tissues were sectioned into 1-mm^3^ pieces on ice and digested with 1 mg/ml collagenase type IV (Sigma-Aldrich, USA) for 20 min at 37 °C with constant agitation. After digestion, samples were sieved through a 70-μm cell strainer (Falcon, USA), and centrifuged at 400* g* for 8 min. And then and the supernatant was discarded. To remove the remaining erythrocytes, 15 mL red blood cell lysis buffer (Beijing Solarbio Science & Technology Co., Ltd., China) was added to the pellet for 15 min on ice. After washing with PBS containing 0.04% BSA, the cell pellets were resuspended in PBS containing 0.04% BSA and re-filtered through a 35-μm cell strainer (Falcon, USA). Dissociated single cells were then stained with AO/PI for viability assessment using Countstar Fluorescence Cell Analyzer. The single-cell suspension was further enriched with a MACS dead cell removal kit (Miltenyi Biotec).

### Single-cell sequencing

The scRNA-Seq libraries were generated through the 10X Genomics Chromium Controller Instrument and Chromium Single Cell 3’ V3.1 Reagent Kits (10X Genomics, Pleasanton, CA, USA). Briefly, cells were concentrated to 1000 cells/µL and approximately 8000 cells were loaded into each channel to generate single-cell Gel Bead-In-Emulsions (GEMs), which results into expected mRNA barcoding of 6000 single cells for each sample. After the RT step, GEMs were broken and barcoded cDNA was purified and amplified. The amplified barcoded cDNA was fragmented, A-tailed, ligated with adaptors, and index PCR amplified. The final libraries were quantified using the Qubit High Sensitivity DNA assay (Thermo Fisher Scientific, USA), and the size distribution of the libraries was determined using a High Sensitivity DNA chip on a Bioanalyzer 2200 (Agilent). All libraries were sequenced by Illumina sequencer (Illumina, San Diego, CA, USA) on a 150-bp paired-end run.

### Single-cell RNA statistical analysis

scRNA-seq data analysis was performed by NovelBio Bio-Pharm Technology Co., Ltd. with NovelBrain Cloud Analysis Platform. We applied fastp [64] with default parameter filtering of the adaptor sequence and removed the low-quality reads to achieve the clean data. Then the feature-barcode matrices were obtained through aligning reads to the human genome (GRCh38 Ensemble: version 91) using CellRanger v3.1.0. We applied the down sample analysis among samples sequenced according to the mapped barcoded reads per cell of each sample and finally achieved the aggregated matrix. Cells contained over 200 expressed genes and mitochondria UMI rate below 20% passed the cell quality filtering and mitochondria genes were removed in the expression table.

Seurat package (version: 3.1.4, https://satijalab.org/seurat/) was used for cell normalization and regression based on the expression table according to the UMI counts of each sample and percent of mitochondria rate to obtain the scaled data. PCA was constructed based on the scaled data with top 2000 high variable genes and top 10 principals were used for tSNE construction and UMAP construction. Utilizing graph-based cluster method, we acquired the unsupervised cell cluster result based the PCA top 10 principal and we calculated the marker genes by FindAllMarkers function with Wilcoxon rank sum test algorithm under following criteria: (1) lnFC > 0.25; (2) *p* value < 0.05; (3) min.pct > 0.1. In order to identify the cell type detailed, the clusters of same cell type were selected for re-tSNE analysis, graph-based clustering, and marker analysis. Since samples were processed and sequenced in batches, we used MNN (mutual nearest-neighbor) to remove potential batch effect. Subsequently, top 10 principals were used for tSNE construction and UMAP construction.

Utilizing graph-based cluster method (resolution = 0.8), we acquired the unsupervised cell cluster result based the PCA top 10 principal and we calculated the marker genes by FindAllMarkers function with Wilcoxon rank sum test algorithm under following criteria: (1) lnFC > 0.25; (2) *p* value < 0.05; (3) min.pct > 0.1. In order to identify the cell type detailed, the clusters of same cell type were selected for re-tSNE analysis, graph-based clustering, and marker analysis.

### Pseudo-time analysis

We applied the Single-Cell Trajectories analysis utilizing Monocle2 (http://cole-trapnell-lab.github.io/monocle-release) by DDR-Tree and default parameter. Before Monocle analysis, we select marker genes of the Seurat clustering result and raw expression counts of the cell passed filtering. Based on the pseudo-time analysis, branch expression analysis modeling (BEAM Analysis) was applied for branch fate-determined gene analysis.

In addition, Monocle 3 were used to infer the cell differentiation trajectories, a package for computing single-cell trajectory analysis [65], in order to analyze decidual stromal cells in early pregnancy based on the previous research data (Vento-Tormo, R., Efremova, M., Botting, R.A., Turco, M.Y., Vento-Tormo, M., Meyer, K.B., Park, J.E., Stephenson, E., Polański, K., Goncalves, A., Gardner, L., Holmqvist, S., Henriksson, J., Zou, A., Sharkey, A.M., Millar, B., Innes, B., Wood, L., Wilbrey-Clark, A., Payne, R.P., Ivarsson, M.A., Lisgo, S., Filby, A., Rowitch, D.H., Bulmer, J.N., Wright, G.J., Stubbington, M.J.T., Haniffa, M., Moffett, A., Teichmann, S.A.. Single-cell reconstruction of the early maternal–fetal interface in humans. The whole-genome sequencing data are deposited at ArrayExpress, E-MTAB-7304 (for the whole-genome sequencing data https://doi.org/10.1038/s41586-018-0698-6)).

### Cell communication analysis

To enable a systematic analysis of cell–cell communication molecules, we applied cell communication analysis based on the CellPhoneDB [23], a public repository of ligands, receptors, and their interactions. Membrane, secreted, and peripheral proteins of the cluster were annotated. Significant mean and cell communication significance (*p* value < 0.05) were calculated based on the interaction and the normalized cell matrix achieved by Seurat normalization.

### SCENIC analysis

To assess transcription factor regulation strength, we applied the single-cell regulatory network inference and clustering (pySCENIC, v0.9.5) workflow [66], using the 20,000 motifs database for RcisTarget and GRNboost.

### QuSAGE analysis (gene enrichment analysis)

To characterize the relative activation of a given gene set such as pathway activation, we performed QuSAGE [67] (2.16.1) analysis.

### Differential gene expression analysis

To identify differentially expressed genes among samples, the function FindMarkers with Wilcoxon rank sum test algorithm were used under following criteria: (1) lnFC > 0.25; (2) *p* value < 0.05; (3) min.pct > 0.1.

### Gene ontology (GO) functional enrichment

Functional enrichment analysis was performed using GO enrichment analysis (http://www.geneontology.org), and each enriched ontology hierarchy (false discovery rate (FDR) < 0.05) was reported with two terms in the hierarchy: (1) the term with the highest significance value and (2) the term with the highest specificity.

### Immunofluorescence

Endometrium and decidua tissue sections were baked at 60 °C for 2 h and deparaffined with dimethylbenzenend rehydrated with ethanol series. Antigen retrieval was performed by boiling tissue sections in Tris–EDTA buffer (pH 9.0) (Beijing Solarbio Science & Technology Co., Ltd., China) or Sodium Citrate Antigen Retrieval Solution (Beijing Solarbio Science & Technology Co., Ltd., China) for 15 min, followed by immediate cooling in cold water for 30 min. Tissue followed by washing twice in PBS for 5 min. Nonspecific binding was blocked with 5% BSA in PBS for 1 h at room temperature. Tissue sections were then incubated with primary antibodies overnight at 4 °C and secondary antibodies for 2 h at room temperature. Primary antibodies and dilution ratios were as follows: IGFBP1 (1:100; no. ab228741, abcam, USA), PRL (1:100; no. ab183967, Abcam, USA), Vimentin (1:100; no. ab8978, Abcam, USA), MMP11 (1:100; no. ab119284, Abcam, USA), ADAMTS5 (1:100; no. ab246975, Abcam, USA), FABP5 (1:50; no. ab255276, Abcam, USA), PLA2G2A (1:100; no. PA5-102403, Invitrogen, USA), RPS2 (1:100; no. ab155961, abcam, USA), DPP4 (1:100; no. ab114033, Abcam, USA), FOXJ1 (1:400, no. 14–9965-82, Invitrogen, USA), HLA-G (1:100; no. ab283260, Abcam, USA), and CK7 (1:100, no. ab185048, Abcam, USA). Secondary antibodies used and dilution ratios were as follows: Goat Anti-Mouse (1:1000, no. ab150113, Abcam, USA) and Goat Anti-Rabbit (1:1000, no. ab150080, Abcam, USA). All sections were counterstained with DAPI (Beijing Solarbio Science & Technology Co., Ltd., China) and mounted buffered glycerol. Images were visualized using fluorescent signals from different lasers and captured using an opticaland epifluorescence microscope (Olympus BX53 Microscope, Olympus Corporation, Japan).

### Immunohistochemistry

The paraffin sections of human endometrium were baked at 60 °C for 2 h, deparaffined with dimethylbenzenend rehydrated with ethanol series. Antigen retrieval was performed by boiling tissue sections in Tris–EDTA buffer (pH 9.0) (Beijing Solarbio Science & Technology Co., Ltd., China) for 15 min. Next the endogenous peroxidase was removed with 3% hydrogen peroxide and incubated with 5% BSA at room temperature for 1 h. And then, the samples were incubated with rabbit anti-IGF1 (1:100, no. DF6096, Affinity Biosciences, USA) or rabbit anti-IGF1 receptor antibody (1:500, no. 263903, Abcam, USA) or rabbit IgG isotypes at 4 °C overnight. After washing with PBS for three times, the sections were incubated with HRP-labeled secondary antibody at room temperature for 30 min, reacted with 3,3-diaminobiphenylamine (DAB), and finally counterstained with hematoxylin.

### Terminal deoxynucleotidyltransferase‑mediated nick end labelling assay (TUNEL)

Cell apoptosis was evaluated using a TUNEL assay kit (Servicebio, Wuhan, China), according to the manufacturer’s instructions. Briefly, paraffin-embedded tissue sections were fixed using 4% paraformaldehyde, and specimens were carried on slides and counterstained with 4′,6-diamidino-2-phenylindole (DAPI) for nuclear localization, without permeabilization. KRT7 was used to label epithelial cells. And then the cells were visualized by fluorescence microscopy and apoptotic cells were marked in both red (fragmented DNA) and blue (nuclear DNA).

### Cell culture experiments

The decidual tissues were digested and isolated as a previous procedure. After centrifugation, the supernatant of single cells was discarded, and the cells were resuspended in DMEM/F-12 containing 10% FBS (Gibco, Germany), plated on culture flasks and incubated in a humidified incubator with 5% CO_2_ at 37 °C. The primary decidual stromal cells (DSCs) were allowed to adhere for 30 min. The culture medium was replaced every 2 days.

Primary DSCs were treated with the vehicle, rh-PRL (0.1 ng/mL, Abcam, USA), rh-IGF1 (2 ng/mL, Abcam, USA), or rh-IL1B (50 ng/mL, R&D Systems, USA) for 48 h. The human endometrial stromal cell line (hESC) and human endometrium epithelial cell line (EEC) were also co-cultured with the HTR‑8/SVneo cells for 24 h. And then these cells were collected, and the expression of related genes was detected by qRT-PCR.

hESCs were treated with the vehicle, estrogen (10 nM, MedChem Express, USA) and MPA (1 μM, MedChem Express, USA), or rh-IGF1 for 48 h. Then the cells were collected to test the expression of PRL, IGFBP1, and IGF1 by qRT-PCR.

In addition, EECs were treated with rh-IL1B (50 ng/mL, R&D Systems, USA) for 48 h to detect the cell apoptosis.

### Isolation and purification of endometrial NK cells

The decidual tissues were digested and isolated as mentioned above. We collected single cells to isolate endometrial NK cells by MASC (human NK cell isolation kit, 130–092-601, Miltenyi Biotec, Germany) for in vitro experiments. These NK cells were treated with rh-AREG (100 ng/mL, MedChem Express, USA) for 48 h, and then NK cells were collected and further analyzed by flow cytometry assays.

### Quantitative real-time polymerase chain reaction (qRT-PCR)

The total RNA of hESCs, hEECs, and DSCs was extracted by TRIzol regent (Trizol, TaKaRa, Japan). Subsequently, the NanoDrop spectrophotometer (NanoDrop Technologies; Thermo Fisher Scientific, MA, USA) was used to quantify the concentration and purity of RNA. The PrimeScript RT Reagent Kit (TaKaRa, Japan) was utilized to reversely transcribe total RNA to cDNA. Next, qRT-qPCR was performed with SYBR Green PCR Master Mix (Yeasen Biotechnology Co., Ltd., Shanghai, China). The qRT-PCR primers are listed in Additional file 24: Table S1. The target mRNA expressions were normalized to ACTB expression. All reactions were processed on the Applied Biosystems 7500 Real-Time PCR System (Thermo Fisher Scientific). The test results were analyzed using the 2^−ΔΔCt^ method.

### Matrigel migration assay

Matrigel (BD Biosciences, USA) was diluted at a ratio of 1:8, and 35 µL was added to the Transwell upper chamber (8 µm, Corning, USA). The Transwell chambers were placed in a 24‑well plate and incubated overnight at 4 °C. Briefly, 200 μL (HTR‑8/SVneo, 1 × 10^5^ cells/well) DMEM/F‑12 suspension without 10% FBS was added to the upper chamber, and 600 μL DMEM/F‑12 containing 10% FBS was added to the lower chamber. According to different experimental requirements, cells pretreated with rh-CSF1(2 ng/mL, R&D Systems, USA) or vehicle were added in the upper chamber. The cells were cultured for 48 h at 37 °C in a 5% CO_2_ incubator. The 24‑well plate was removed, and the upper chamber medium and nonpenetrating cells were gently wiped off with a cotton swab, washed three times with phosphate buffered saline (PBS), fixed with 4% paraformaldehyde for 30 min, and stained with crystal violet for 20 min. Thereafter, random photographs were acquired under an inverted microscope (× 200), and 5 visual fields were counted in each chamber. The number of invaded cells was counted using ImageJ software (National Institute of Mental Health, USA).

### Reactive oxygen species detection

Reactive oxygen species (ROS) were detected in different groups (control, rh-IL1B) using a 2,7‑dichlorofluorescin diacetate (DCFH‑DA) assay (Beyotime Institute of Biotechnology, Haimen, China). After 48 h of treatment with rh-IL1B (50 ng/mL, 201-LB-025, R&D Systems, USA), DCFH‑DA (10 µmol/L) was added to each well. After incubation for 20 min at 37 °C, the cells were rinsed with PBS and analyzed by flow cytometry. ROS levels were analyzed using FlowJo software (version 10.07 (FlowJo LLC, USA), and the results were calculated relative to the control group.

### Flow cytometry assays

Human antibodies for flow cytometry assays (all antibodies were purchased Biolegend, CA, USA, Invitrogen, USA, BD Pharmingen, USA or R&D Systems, USA) were used for measurement of cell markers, listed in Additional file 25: Table S2. Isotype IgG antibody (5 μl separately) was used as the control. Human Trustain FcX (Biolegend) was used to block Fc receptors prior to flow cytometry. Subsequently, cells were washed twice and resuspended in PBS for flow cytometry analysis. Samples were analyzed using a CytoFLEX flow cytometer (Beckman Coulter, Inc., USA), and data were analyzed using FlowJo.

### Annexin V/PI apoptosis assay

Apoptotic cell death was detected via Annexin V-APC/ propidium iodide (PI) staining using the apoptosis kit (APC Annexin V Apoptosis Detection Kit with PI, Biolegend, USA). DSCs and hEECs (5 × 10^5^ cells) from the various cultures were trypsinized using 0.25% Trypsin (1 × , Phenol Red; no EDTA; Gibco) for 3 min at 37 °C with 5% CO_2_ and collected, washed, and resuspended in 100 μL binding buffer included in the apoptosis kit, followed by incubation with 5 μL Annexin V-APC and 10 μL PI at room temperature for 15 min in the dark. Then, 400 μL binding buffer was added and the cell samples were analyzed with a Beckman CytoFLEX S flow cytometer (Beckman Coulter, Inc., USA) using FlowJo software. Annexin V^+^ PI^−^ cells were in the early stage of apoptosis and Annexin V^+^ PI^+^ cells were late apoptotic cells.

### Integration analysis of the protein–protein interaction (PPI) network

The STRING database (available online: http://string-db.org) was performed for protein–protein interaction network prediction.

### Statistics

The results were representative of multiple experiments and were presented as mean ± SEM. The variables were analyzed by Student’s *t* test between two groups or a one-way ANOVA using Tukey’s post hoc test in multiple groups (STATA, version 15, StataCorp, USA). The differences were considered as statistically significant at *P* < 0.05.

## Supplementary Information

**Additional file 1.**

**Additional file 2.**

**Additional file 3.**

**Additional file 4.**

**Additional file 5.**

**Additional file 6.**

**Additional file 7.**

**Additional file 8.**

**Additional file 9.**

**Additional file 10.**

**Additional file 11.**

**Additional file 12.**

**Additional file 13.**

**Additional file 14.**

**Additional file 15.**

**Additional file 16.**

**Additional file 17.**

**Additional file 18.**

**Additional file 19.**

**Additional file 20.**

**Additional file 21.**

**Additional file 22.**

**Additional file 23.**

**Additional file 24.**

**Additional file 25.**

**Additional file 26.**

## Data Availability

All data generated or analyzed during this study are included in this published article, its supplementary information files and publicly available repositories. Raw data for single-cell RNA-seq are available on the NCBI’s Gene Expression Omnibus (GEO) database under the accession number GSE183837 (endometrium) and GSE194219 (decidua). The data of Fig. 1B based on the previous research data (Vento-Tormo, R., Efremova, M., Botting, R.A., Turco, M.Y., Vento-Tormo, M., Meyer, K.B., Park, J.E., Stephenson, E., Polański, K., Goncalves, A., Gardner, L., Holmqvist, S., Henriksson, J., Zou, A., Sharkey, A.M., Millar, B., Innes, B., Wood, L., Wilbrey-Clark, A., Payne, R.P., Ivarsson, M.A., Lisgo, S., Filby, A., Rowitch, D.H., Bulmer, J.N., Wright, G.J., Stubbington, M.J.T., Haniffa, M., Moffett, A., Teichmann, S.A.. Single-cell reconstruction of the early maternal–fetal interface in humans. The whole-genome sequencing data are deposited at ArrayExpress, E-MTAB-7304 (for the whole-genome sequencing data https://doi.org/10.1038/s41586-018-0698-6)). All other relevant data are summarized in supplemental data.

## Abbreviations

RIF: Recurrent implantation failure
EVT: Extravillous trophoblasts
ESC: Endometrial stromal fibroblast cells
DSC: Decidual stromal cell
Camp: Cyclic adenosine monophosphate
PRL: Prolactin
IGFBP-1: Insulin-like growth factor binding protein 1
PRL: Pregnancy loss
WOI: Window of implantation
IVF-ET: In vitro fertilization-embryo transfer
IGF1: Insulin-like growth factor 1
scRNA-seq: Single-cell RNA sequencing
IC: Immune cell
ECM: Extracellular matrix
eMSC: Endometrial mesenchymal stem cells
SC: Stromal cell
EPC: Epithelial cells
EC: Endothelial cell
LIF: Leukemia inhibitory factor
IHH: Indian hedgehog
DPP4: Dipeptidyl Peptidase 4
ADCY1: Adenylate cyclase 1
MPA: Medroxyprogesterone
E2: Estradiol
EEC: Endometrial epithelial cell
DC: Dendritic cell
PPI: Protein-protein interaction
