# Supplementary material for: An IGF1-expressing endometrial stromal cell population is associated with human decidualization
Source: BMC Biol. 2022 Dec 8;20:276. doi: 10.1186/s12915-022-01483-0 (PMC9733393; doi:10.1186/s12915-022-01483-0)
Supplement: Supplementary file 1 — Additional file 1. [file 12915_2022_1483_MOESM1_ESM.docx]

**Supplement figure legends**

**Figure S1: The atlas of human endometrium before and after embryo implantation**

1. Flowchart overview of the scRNA-seq and methodology.
2. UMAP plots on single cells in endometrium from healthy controls at the WOI timing (Endometrium, n=3) and normal decidua from early pregnancy (Decidua, n=3), indicating 18 clusters.
3. Violin plots of representative marker genes for 18 clusters and 6 cell subsets. X-axis is log scale normalized read count.
4. The proportion of SC, IC, EPC and EC and total cell number from different samples.
5. The proportion of cells from Endometrium and Decidua in major six clusters.

SC: stromal cells, IC: immune cells, EPC: epithelial cells, EC: endothelial cells.

**Figure S2: Cell distribution of endometrium before and after embryo implantation**

1. Cell distribution from different samples on UMAP plots.
2. The proportion of four major type cells from Endometrium and Decidua.

SC: stromal cells, IC: immune cells, EPC: epithelial cells, EC: endothelial cells.

**Figure S3 Subtypes of endometrial stromal cells**

1. Heat map showed the expression of differentially marker genes (z-score) for each cluster of stromal cells.
2. Significant gene markers for each cluster were selected to perform GO analysis. GO terms with P < 0.05 are shown. Gene number of each GO term is listed on the left. P value is shown as –log 10 (P value).

**Figure S4 The distribution and proportion of endometrial stromal cells from Endometrium and Decidua**

1. UMAP map of stromal cells of Endometrium and Decidua.
2. The proportion of seven sub-clusters (PreSec_SC, Sec_SC, dSec_SC, Rem_SC, dRem_SC, Pro_SC and eMSC) of stromal cells from Endometrium and Decidua groups.
3. Volcano plot showing differentially expression genes of stromal cells between Endometrium and Decidua groups. Red color indicates genes that are upregulated and blue color indicates downregulated genes in Endometrium compared with Decidua. The GO term of related genes is listed on the right.
4. Rose diagrams indicating the number of upregulated (red) and downregulated (green) genes in stromal cells from Endometrium and Decidua.

**Figure S5 Expression of decidualization related genes in endometrial stromal cells**

1. Bubble diagram showing the average expression of decidualization related genes of five sub-clusters of SC.
2. Expression of decidualization related genes in Violin plots in five sub-clusters of stromal cells

**Figure S6 The distribution of Rem_SC and dRem_SC according to pseudotime analysis**

1. UMAP map showed the distribution of Rem_SC and dRem_SC.
2. Monocle pseudotime trajectory showing the progression of Rem_SC and dRem_SC.
3. Monocle pseudotime map showing the distribution of Rem_SC and dRem_SC from different samples.

**Figure S7 Expression patterns of representative genes in two subtypes of stromal cells along the reprogramming trajectory**

A,B. The expression of the genes in a branch-dependent manner. Each row indicated the standardized kinetic curves of a gene. From the left to the right of the heatmap, the kinetic curve progresses from the Rem_SC along the trajectory to the dRem_SC (left). The genes were categorized into 3 clusters based on their characteristic expression dynamics, and the enriched GO terms for each gene cluster were identified (right). Expression patterns of representative genes along the reprogramming trajectory (below).

**Figure S8 IGF1 is involved in the regulation of decidualization**

1. The PPI network of IGF1, decidualization receptivity-related genes and ECM-related genes.
2. hESCs were treated with the E2 (10nM) +MPA (1μM) or vehicle for 48h. And then the mRNA expression levels of *IGF1* in hESCs were measured by qRT-PCR (n=6).
3. hESCs were treated with the E2 (10nM) +MPA (1μM), rh-IGF1 (2ng/mL), or vehicle for 48h. And then the mRNA expression levels of *IGFBP1* and *PRL* in hESCs were measured by qRT-PCR (n=6).
4. hESCs were treated with the rh-IGF1 (2ng/mL), rh-PRL (0.1ng/mL) or vehicle for 48h. And then the mRNA expression levels of *ADCY1* and *ADCY3* in hESCs were measured by qRT-PCR (n=6). (ns, no significance; **, p<0.01; ***, p<0.001;)

**Figure S9 The function of three sub-clusters of stromal cells**

1. Heat map showed the enrichment of pathway (z-score) for three sub-clusters of stromal cells.
2. Heat map showed the enrichment of metabolism pathway (z-score) for three sub-clusters of stromal cells.
3. Dot plots showing the expression of the IL1B, IL1R1 and IL1R2 on the UMAP map in stromal cells.

**Figure S10** **Subtypes of endometrial epithelial cells**

1. Heat map showed the expression of differentially marker genes (z-score) for each cluster of epithelial cells.
2. Bubble diagram showing the average expression of decidualization related genes of three sub-clusters of epithelial cells.
3. Significant gene markers for three sub-clusters in epithelial cells were selected to perform GO analysis. GO terms with P < 0.05 are shown. Gene number of each GO term is listed on the left. P value is shown as –log 10 (P value).

**Figure S11 IL1B is predicted to regulate the apoptosis of epithelial cells**

1. Immunofluorescence staining of PLA2G2A and DPP4 in the decidua (n=3). The red fluorescence indicates PLA2G2A^+^ stromal cells (arrows). Scale bar, 50μm (left), 20μm (right).
2. The PPI network of IL1B and apoptosis-related genes.

**Figure S12 Subtypes of endometrial immune cells**

1. The cell cluster of IC was re-clustered into three sub-clusters visualized by UMAP.
2. Expression of representative marker genes in Violin plots.
3. Cell distribution of IC from different samples on UMAP plots.

SC: stromal cells, IC: immune cells, EPC: epithelial cells, EC: endothelial cells.

**Figure S13 Immune cells in the endometrium**

Gating strategy for a panel of four antibodies to analyze the different subsets of immune cells in endometrium and decidua by flow cytometry.

**Figure S14 Subtypes of macrophage**/**DC**

1. The cell cluster of macrophage/DC was re-clustered into seven sub-clusters visualized by UMAP.
2. Expression of representative marker genes of macrophage/DC in Bubble diagram.
3. Gating strategy for a panel to analyze the M1, M2 and M3 of macrophage in Endometrium and Decidua group by flow cytometry.

**Figure S15 Characteristics of macrophage subtypes**

1. Potential interactions between three macrophage (M1, M2 and M3) and six other cells (Endothelial, Epithelial, EVT, NK cell, Stromal cell, and T cell) based on receptor-ligand pairs. And ligand genes and receptor genes involved each cluster were selected to perform pathway analysis (right). Pathway with P < 0.05 are shown. Gene number of each pathway is listed on the left. P value is shown as –log 10 (P value).
2. Heat map showed the expression of biological function-related genes (z-score) for each cluster of macrophages.
3. The proportion of seven sub-clusters of macrophages from Endometrium and Decidua.
4. Dot plots showing the expression of the INHBA and INHA on the UMAP map in stromal cells.

**Figure S16 Subtypes of NK cells**

A,B. Gating strategy for a panel to analyze the NK1, NK2, NK3 and NK4 of NK cell in Endometrium and Decidua by flow cytometry.

**Figure S17 The potential function of NK cells in the endometrium.**

1. Heat map showed the enrichment of other pathway (z-score) for five sub-clusters of NK cell.
2. Heat map showed the enrichment of metabolism pathway (z-score) for five sub-clusters of NK cell.

**Figure S18 The potential interactions between NK cells and other endometrium cells**

A,B. The proportion of five sub-clusters of NK cell from Endometrium and Decidua

C. Circos plot of interaction network between four sub-clusters (NK1, NK2, NK3, NK4) and other cells (stromal cell, EVT, endothelial cell, epithelial cell) (left). The ribbons connect each ligand to the assigned receptors. The color of a ribbon is consistent with the color at the receptor side to ligand side. And ligand genes and receptor genes involved each cluster were selected to perform pathway analysis (right). Pathway with P < 0.05 are shown. Gene number of each pathway is listed on the left. P value is shown as –log 10 (P value).

D. Circos plot of interaction network between four sub-clusters (NK1, NK2, NK3, NK4) and other immune cells (macrophage and T cell) (left). The ribbons connect each ligand to the assigned receptors. The color of a ribbon is consistent with the color at the receptor side to ligand side. And ligand genes and receptor genes involved each cluster were selected to perform pathway analysis (right). Pathway with P < 0.05 are shown. Gene number of each pathway is listed on the left. P value is shown as –log 10 (P value).

E. The invasion of HTR‑8/SVneo cells in the control group and rh-CSF1 (2ng/mL) group was detected by Transwell assay (n=3).

F. The effects of rh-CSF1(2ng/mL) on *MMP2* and *MMP9* expression in HTR‑8/SVneo cells were analyzed by RT‑PCR (n=6). Data were presented as mean ± SEM and analyzed by t test. (**, p<0.01; ***, p<0.001)

**Figure S19 Cell communication networks of EVT and endometrial cells**

1. Circos plot of interaction network between EVT and endometrial cells (stromal cell, NK cell, macrophage, T cell, endothelial cell, epithelial cell) (left). The ribbons connect each ligand to the assigned receptors. The color of a ribbon is consistent with the color at the receptor side to ligand side. And ligand genes and receptor genes involved each cluster were selected to perform pathway analysis (right). Pathway with P < 0.05 are shown. Gene number of each pathway is listed on the left. P value is shown as –log 10 (P value).
2. Bubble diagram showing average expression of decidualization related genes of epithelial cells in the Endometrium and Decidua.
3. hEECs were co-cultured with the HTR‑8/SVneo or vehicle for 24h. And then the mRNA expression levels of these genes in hEECs were measured by qRT-PCR (n=6). Data were presented as mean ± SEM and analyzed by t test. (ns, no significance; *, p<0.05; **, p<0.01; ***, p<0.001)

**Figure S20 Cell communication networks of EVT and other cells in the endometrium**

Overview of selected ligand–receptor interactions between EVT and other cells; P values indicated by circle size, scale on right. The means of the average expression level of interacting molecule 1 in cluster 1 and interacting molecule 2 in cluster 2 were indicated by color.

**Figure S21 EVT regulates decidualization**

1. violin plots showed the expression of PRL, CSH1 for each cluster of epithelial cells and EVTs.
2. The PPI network of AREG, CSF1, IGF1 and related genes.

**Figure S22 Cell communication networks in the endometrium**

Overview of selected ligand–receptor interactions in the endometrium; P values indicated by circle size, scale above. The means of the average expression level of interacting molecule 1 in cluster 1 and interacting molecule 2 in cluster 2 were indicated by color.

**Figure S23 Cell communication networks of stromal cells and other cells in the endometrium**

Overview of selected ligand–receptor interactions between five sub-clusters of SC and other cells; P values indicated by circle size, scale above. The means of the average expression level of interacting molecule 1 in cluster 1 and interacting molecule 2 in cluster 2 were indicated by color.
