# Supplementary figures and images for: An IGF1-expressing endometrial stromal cell population is associated with human decidualization
Source: BMC Biol. 2022 Dec 8;20:276. doi: 10.1186/s12915-022-01483-0 (PMC9733393; doi:10.1186/s12915-022-01483-0)

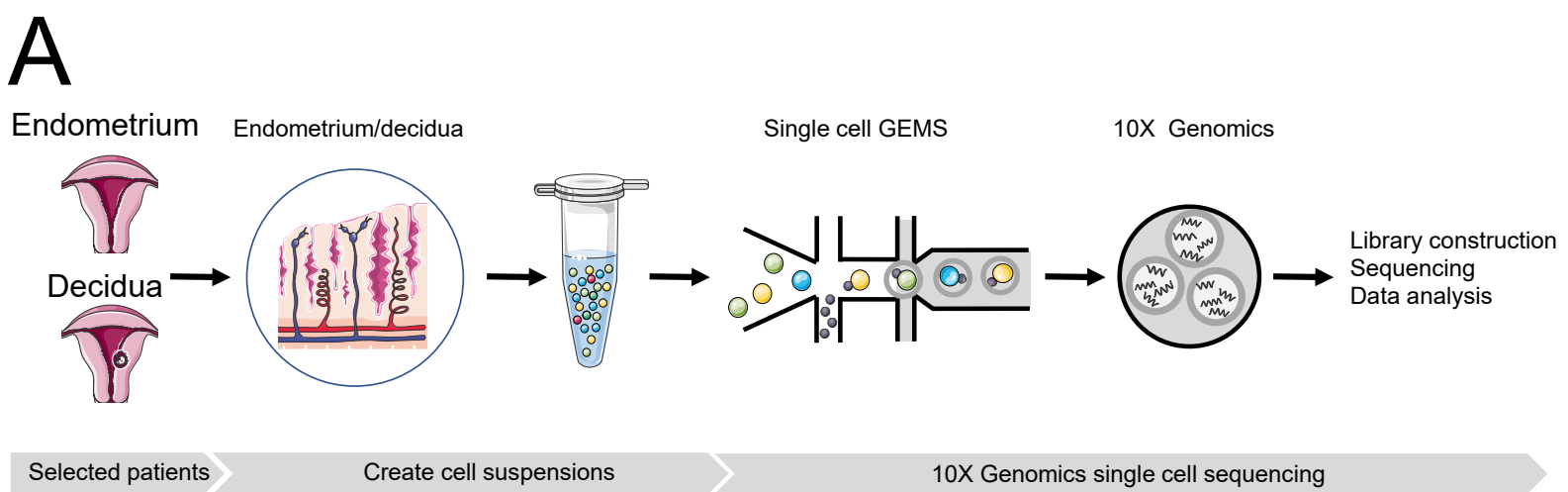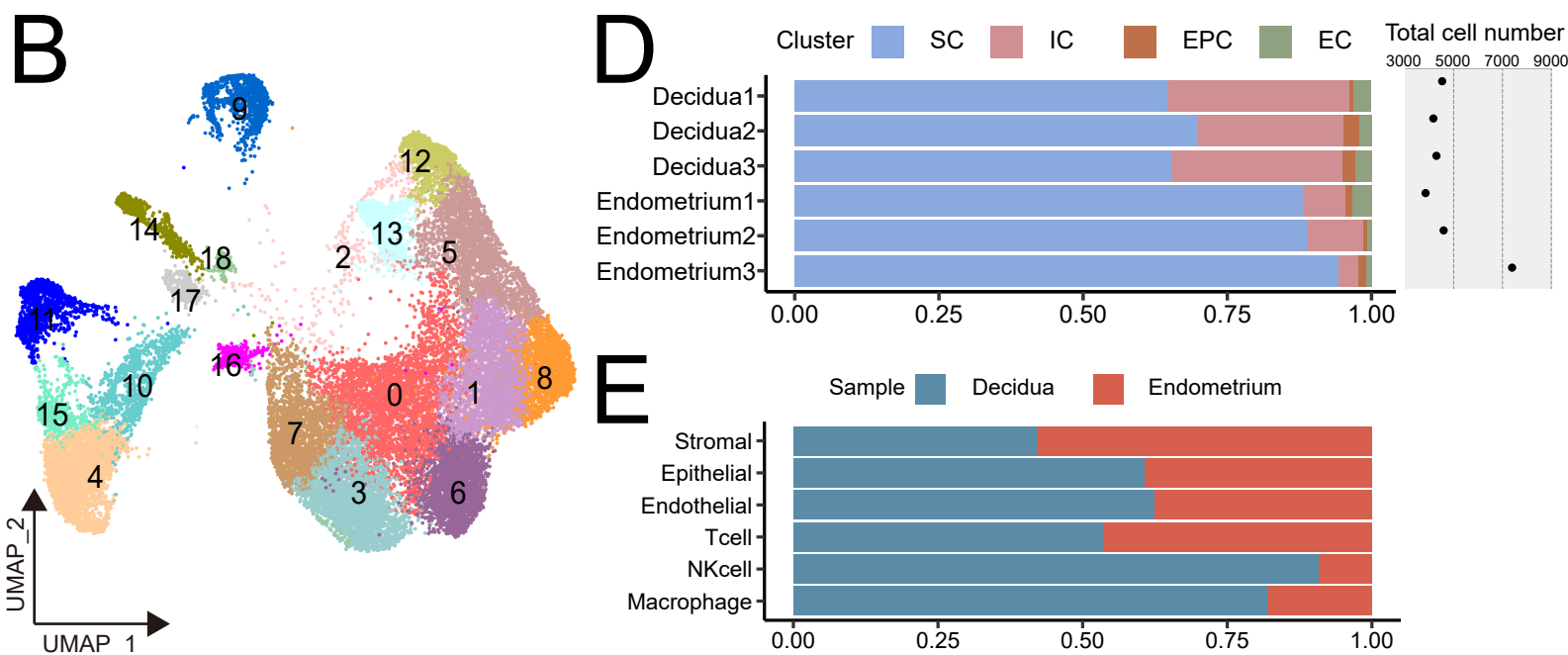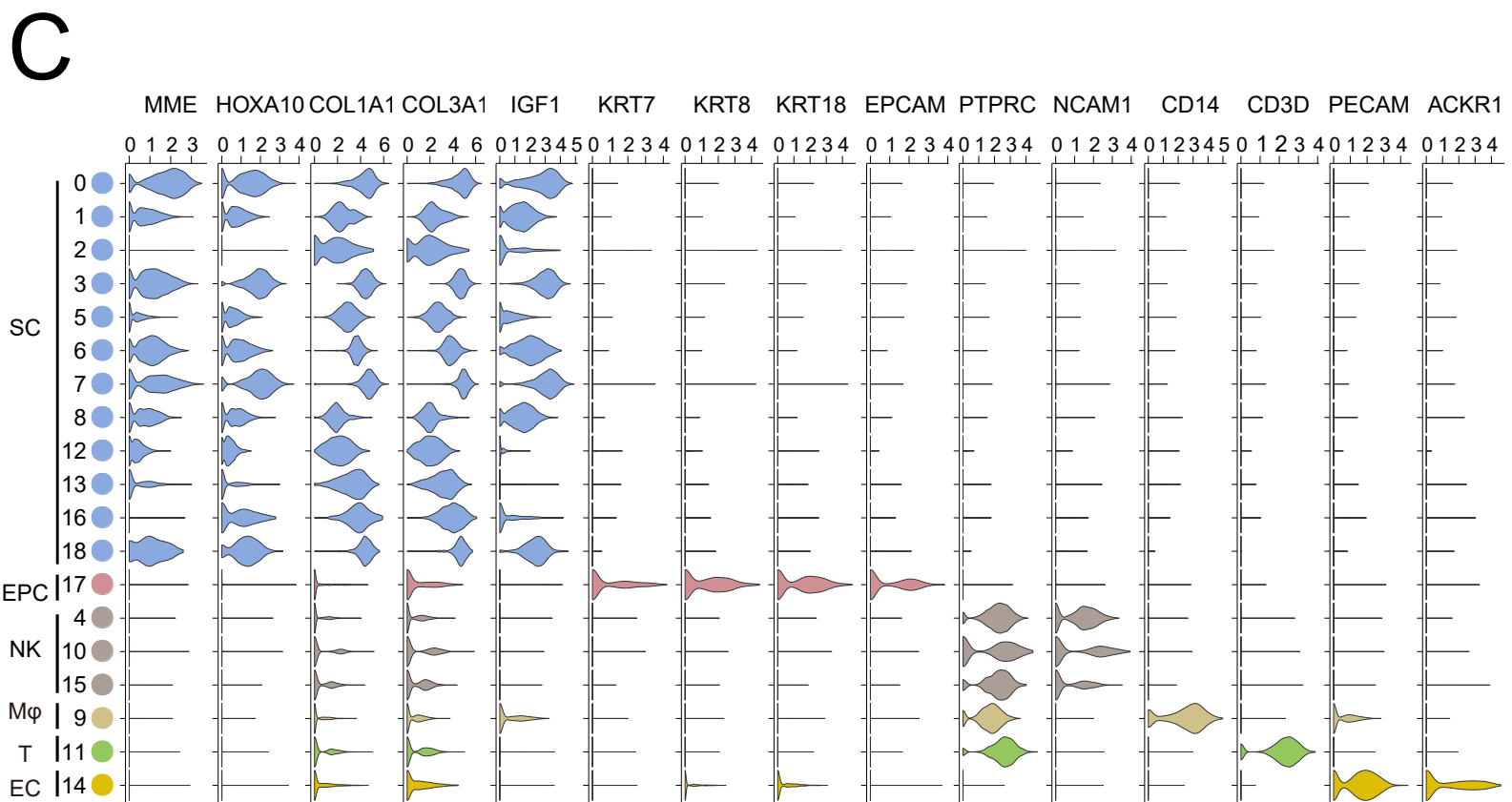

Supplement: Supplementary file 2 — Additional file 2. [file 12915_2022_1483_MOESM2_ESM.pdf]

**A**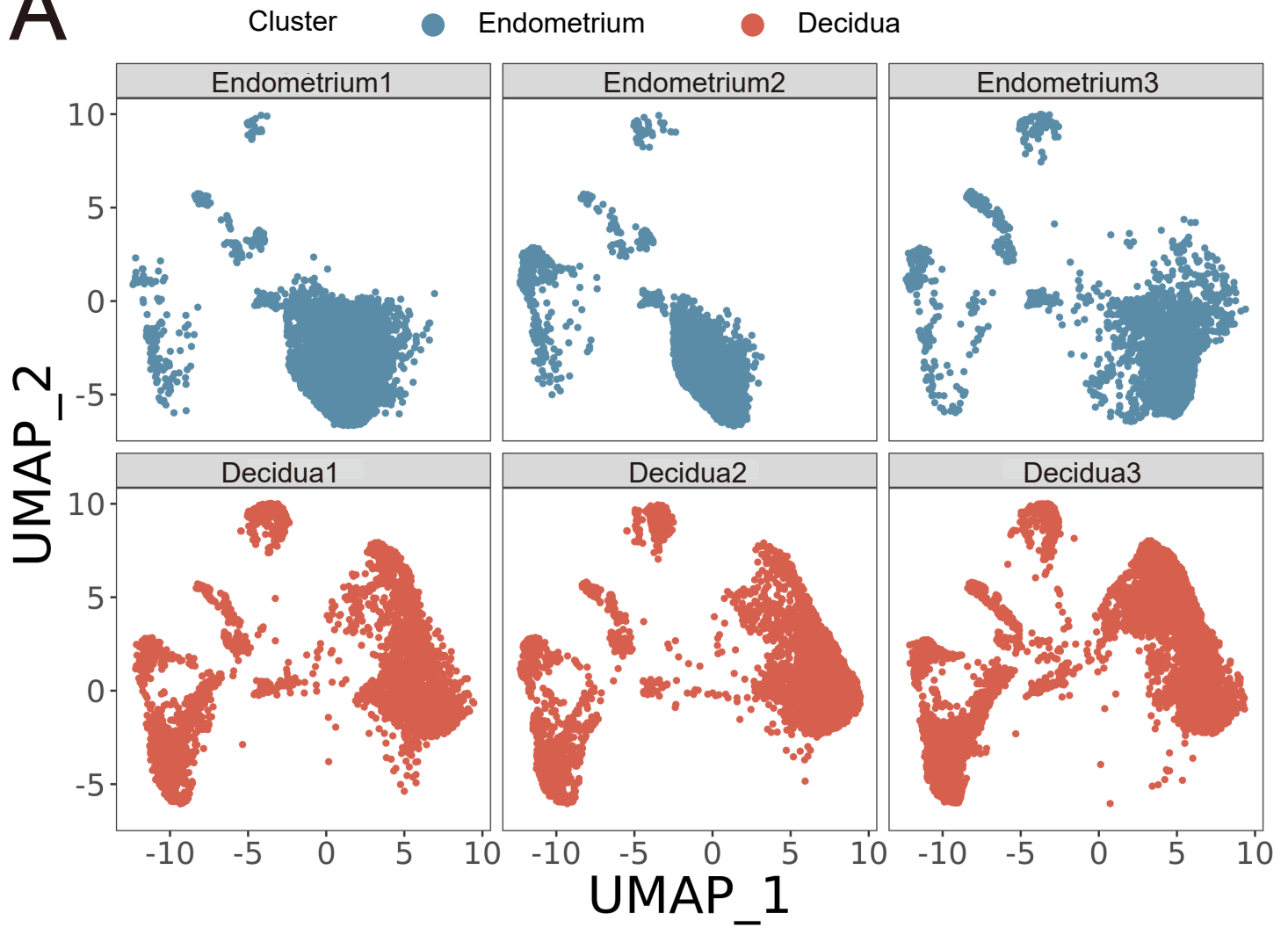**B**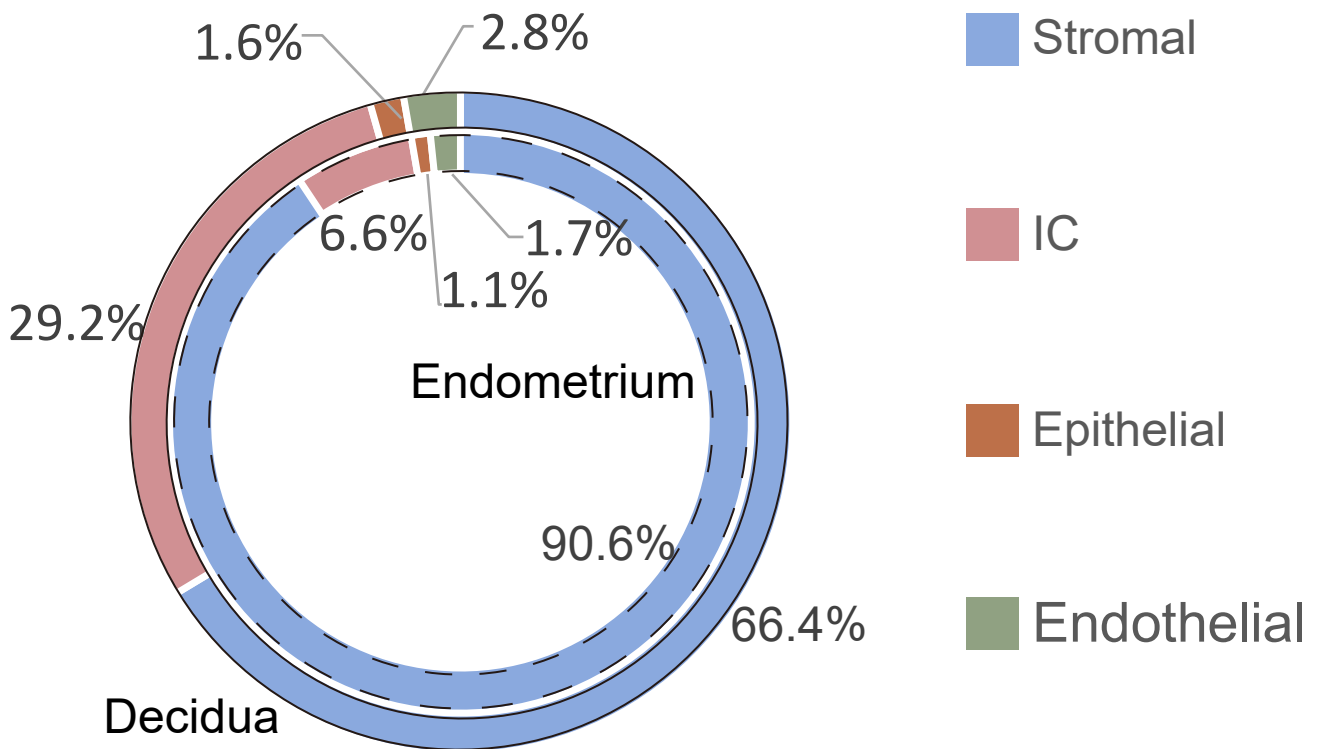

Supplement: Supplementary file 3 — Additional file 3. [file 12915_2022_1483_MOESM3_ESM.pdf]

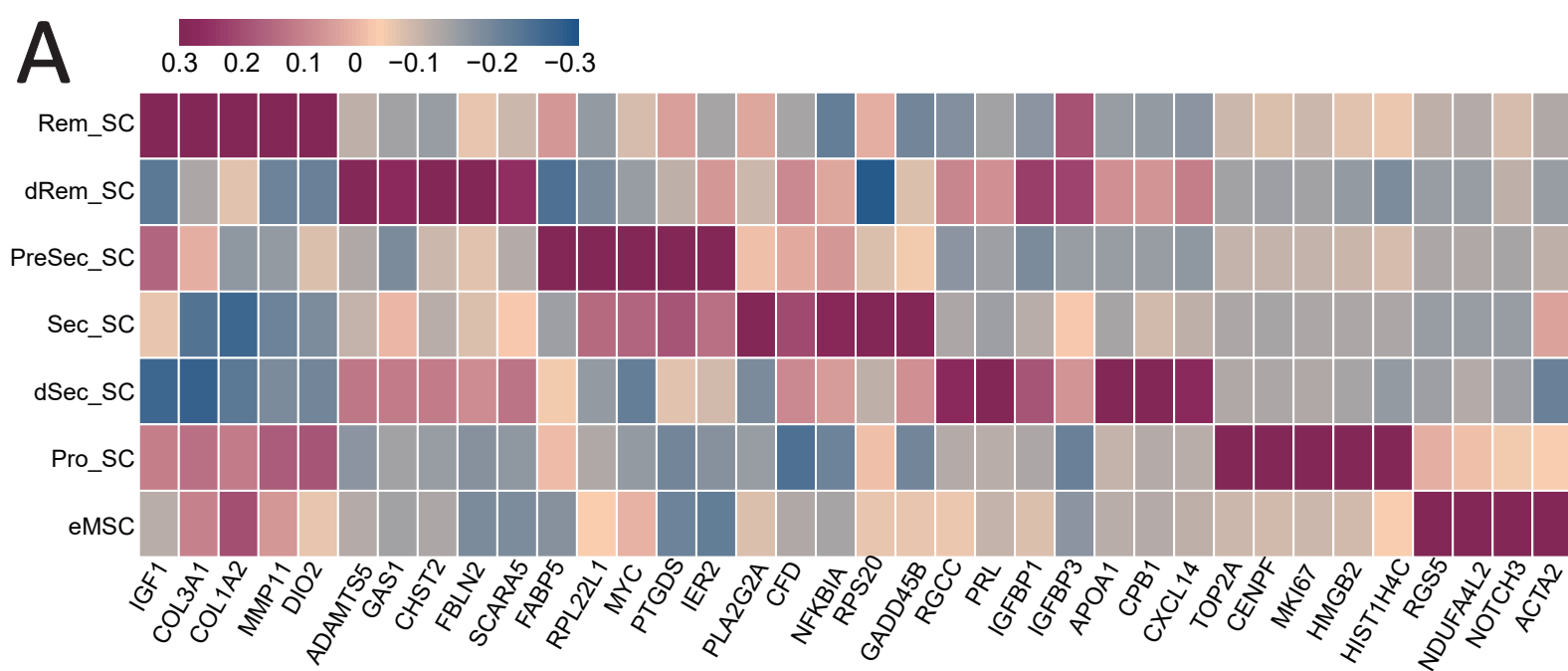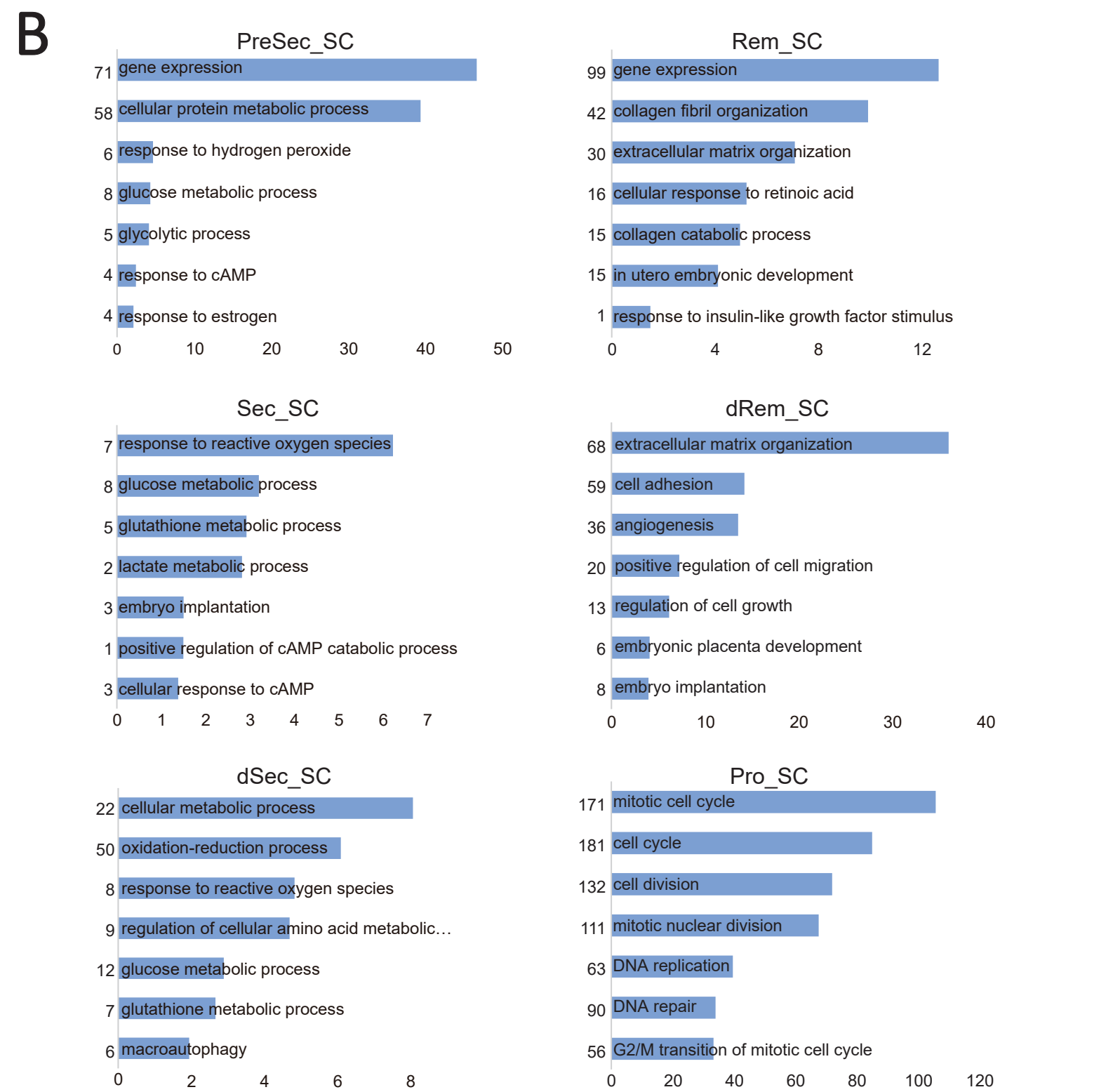

Supplement: Supplementary file 4 — Additional file 4. [file 12915_2022_1483_MOESM4_ESM.pdf]

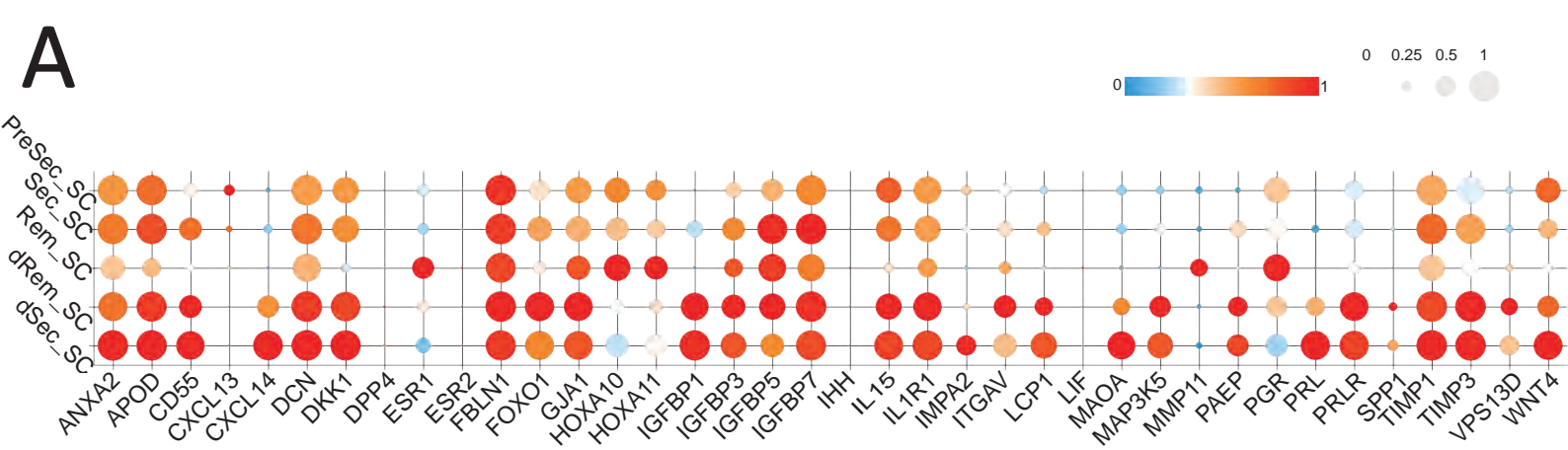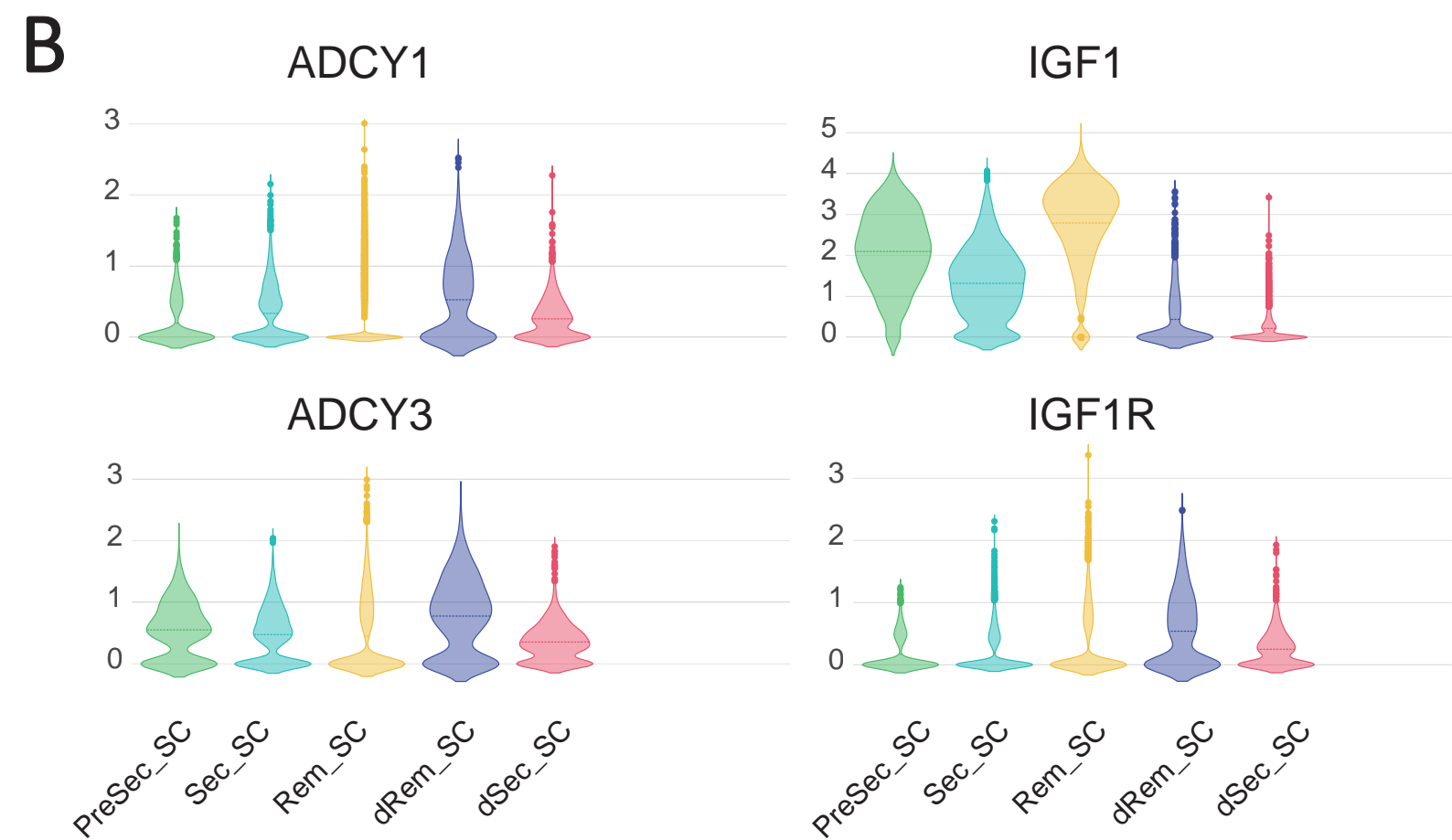

Supplement: Supplementary file 6 — Additional file 6. [file 12915_2022_1483_MOESM6_ESM.pdf]

**A**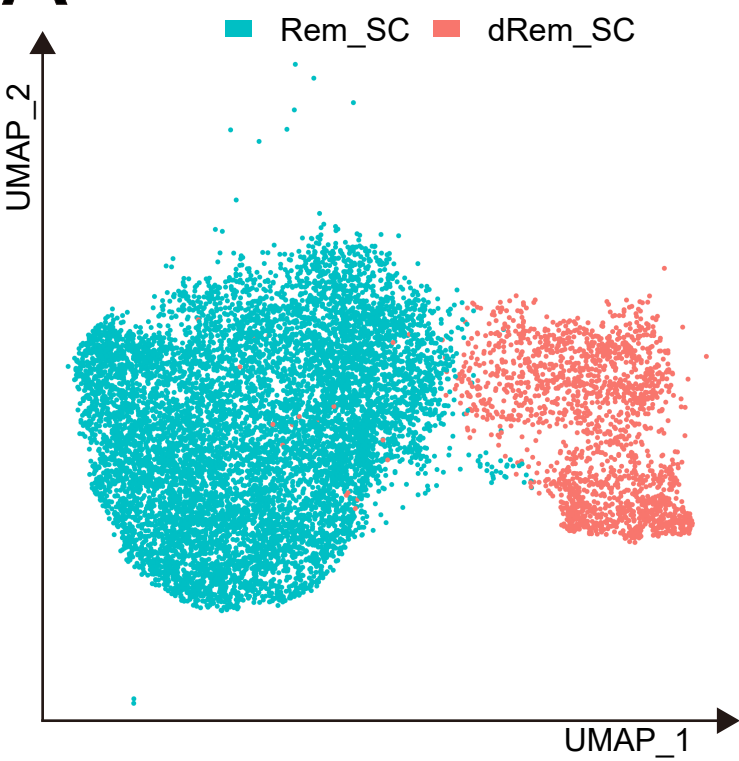**B**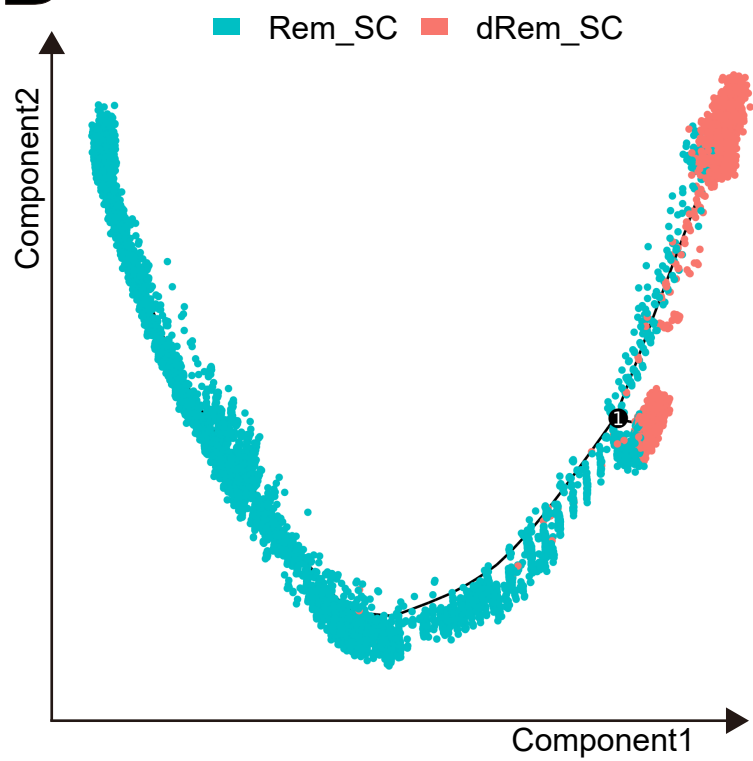**C**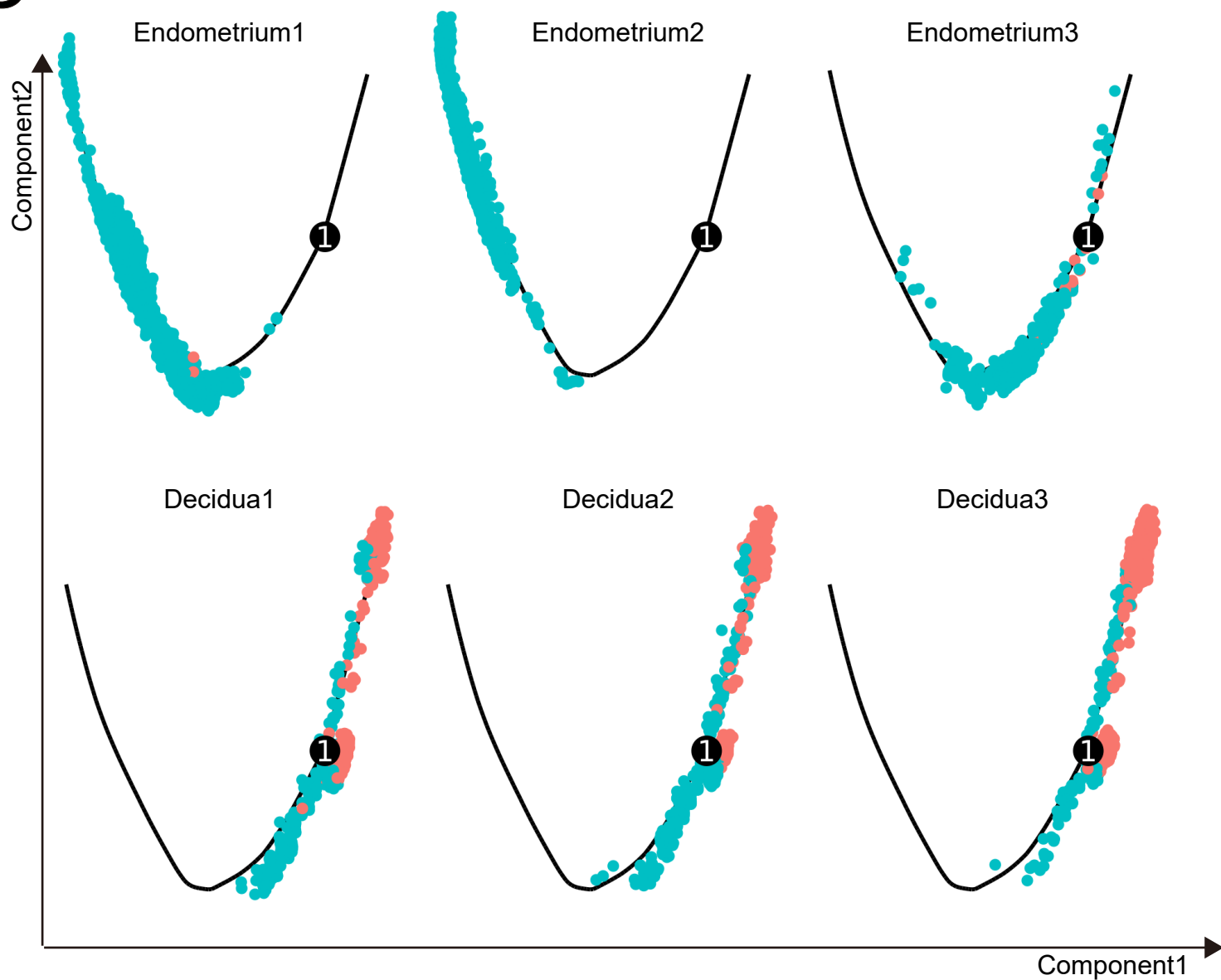

Supplement: Supplementary file 7 — Additional file 7. [file 12915_2022_1483_MOESM7_ESM.pdf]

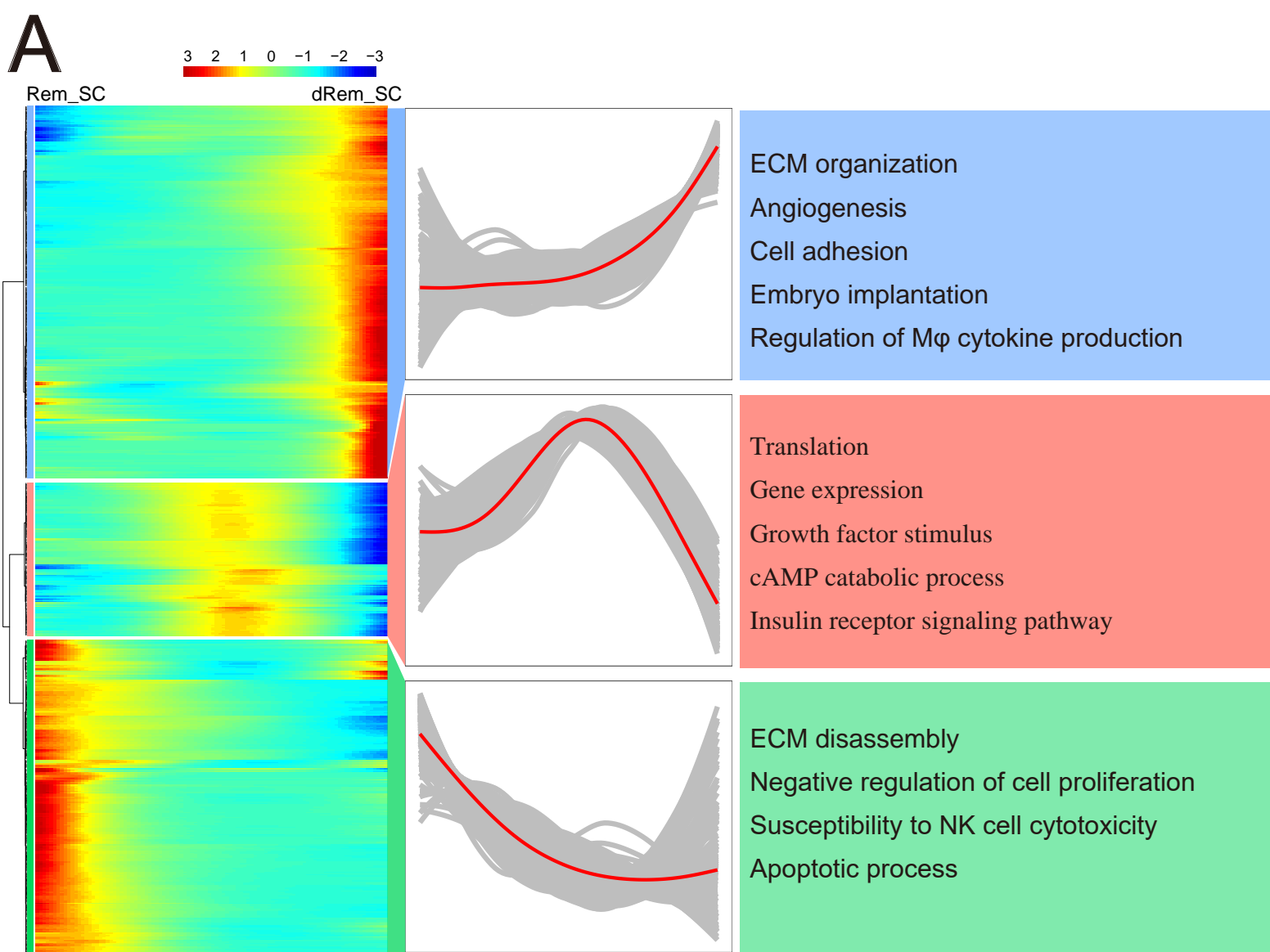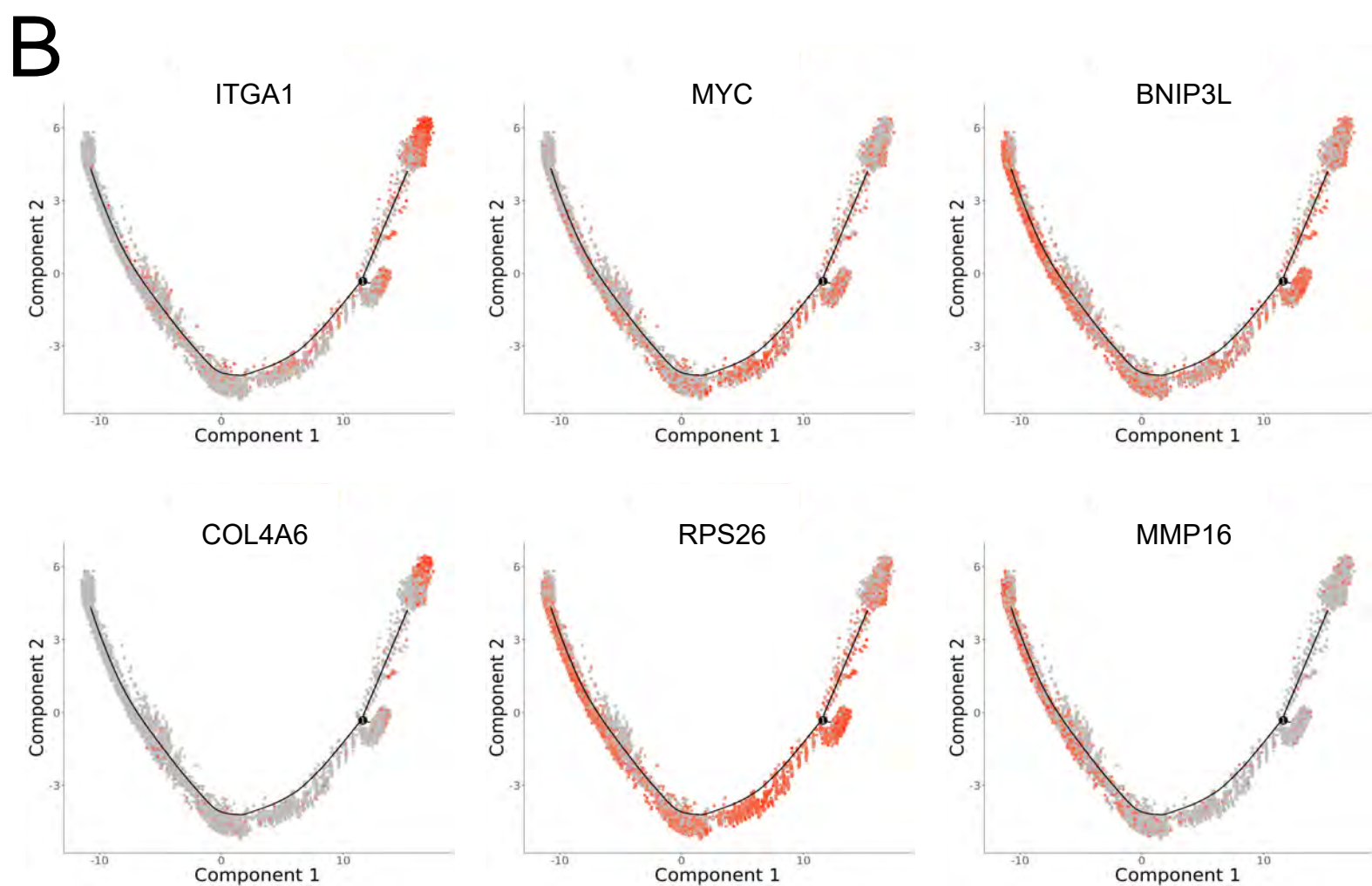

Supplement: Supplementary file 8 — Additional file 8. [file 12915_2022_1483_MOESM8_ESM.pdf]

# A

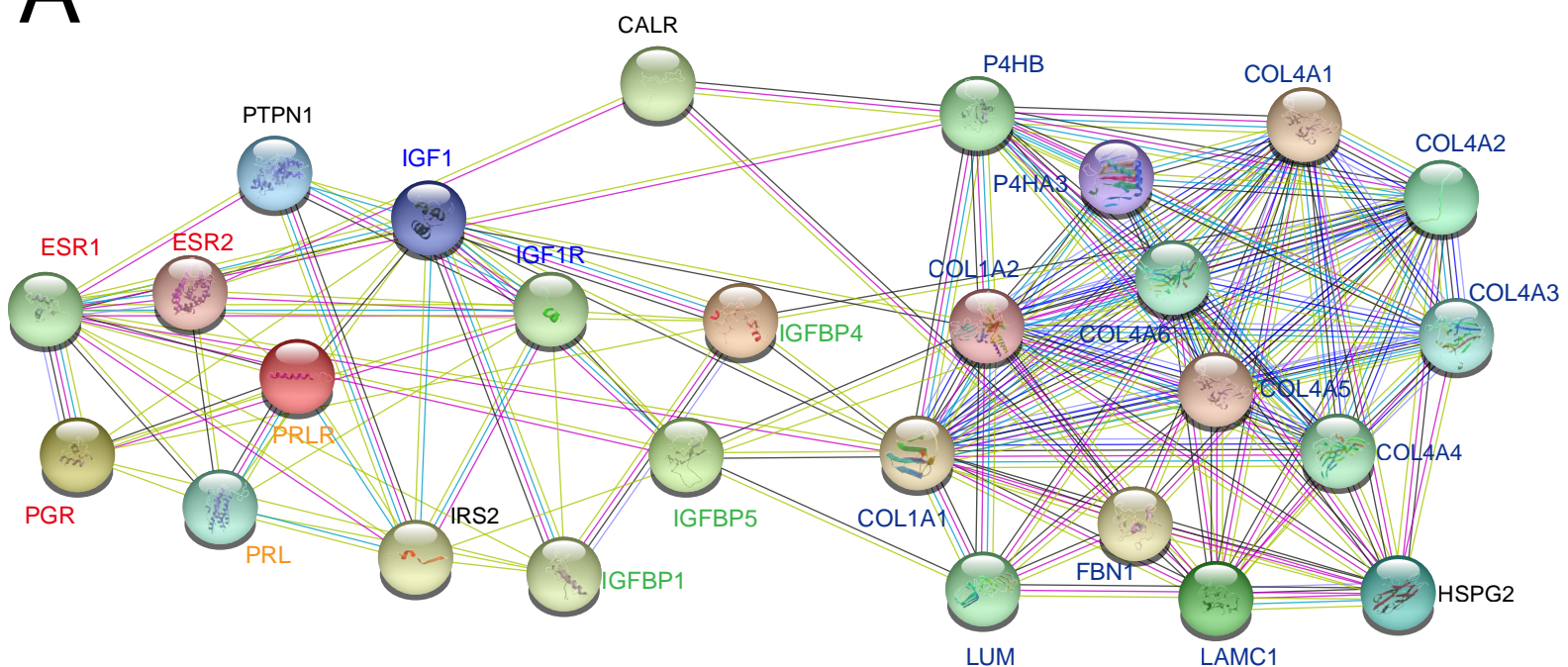

# B

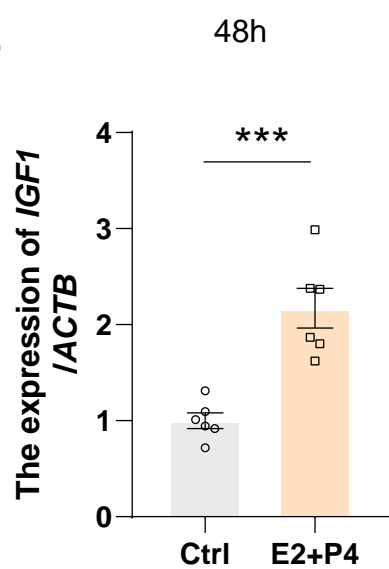

# C

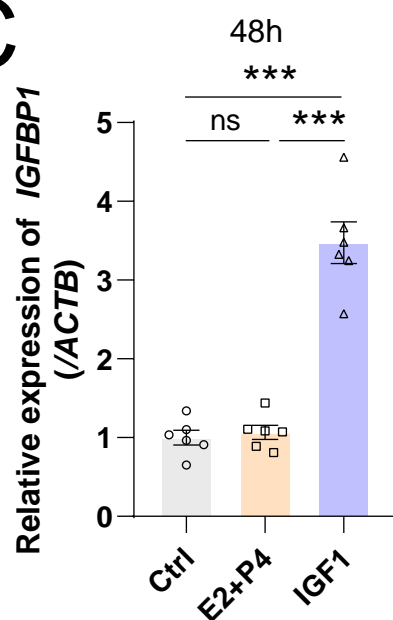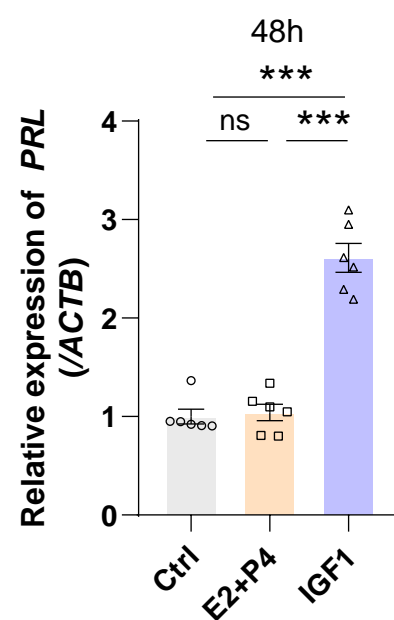

# D

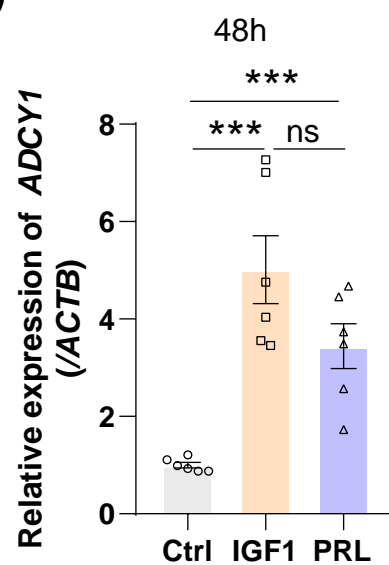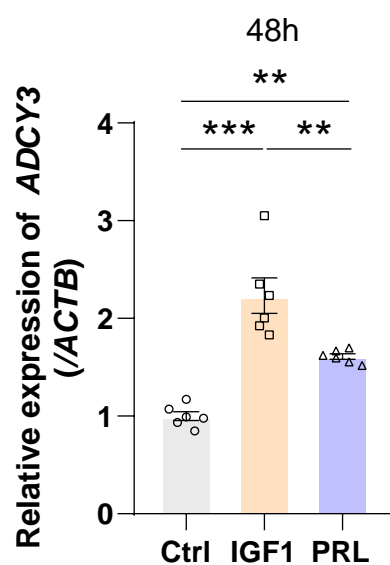

Supplement: Supplementary file 9 — Additional file 9. [file 12915_2022_1483_MOESM9_ESM.pdf]

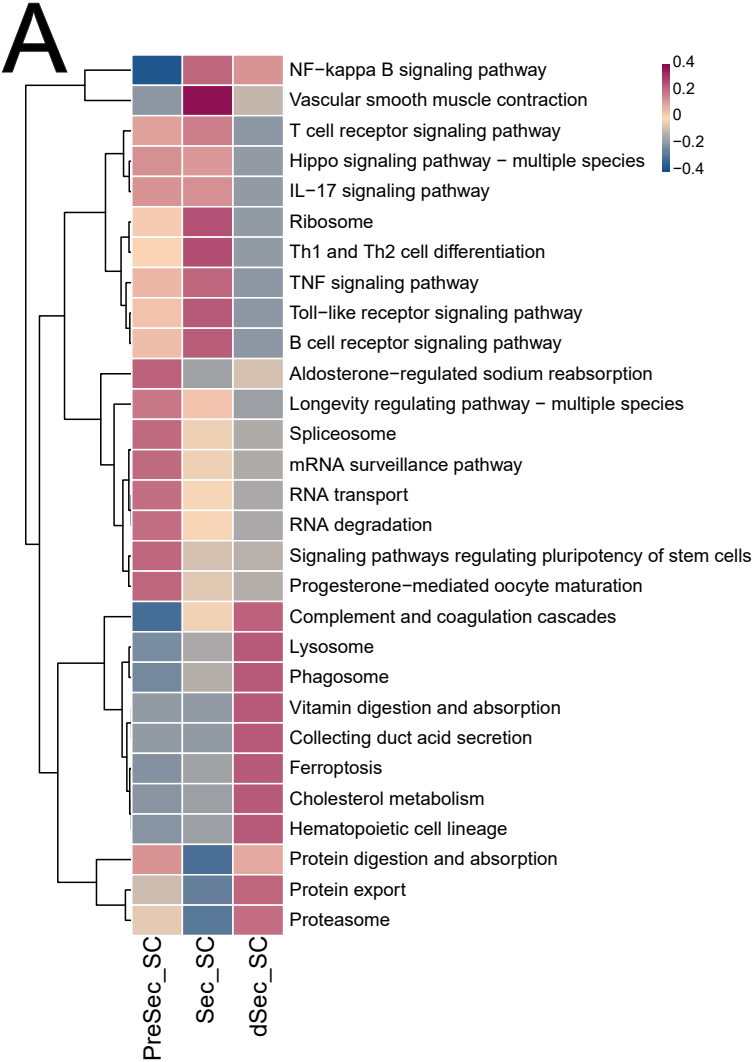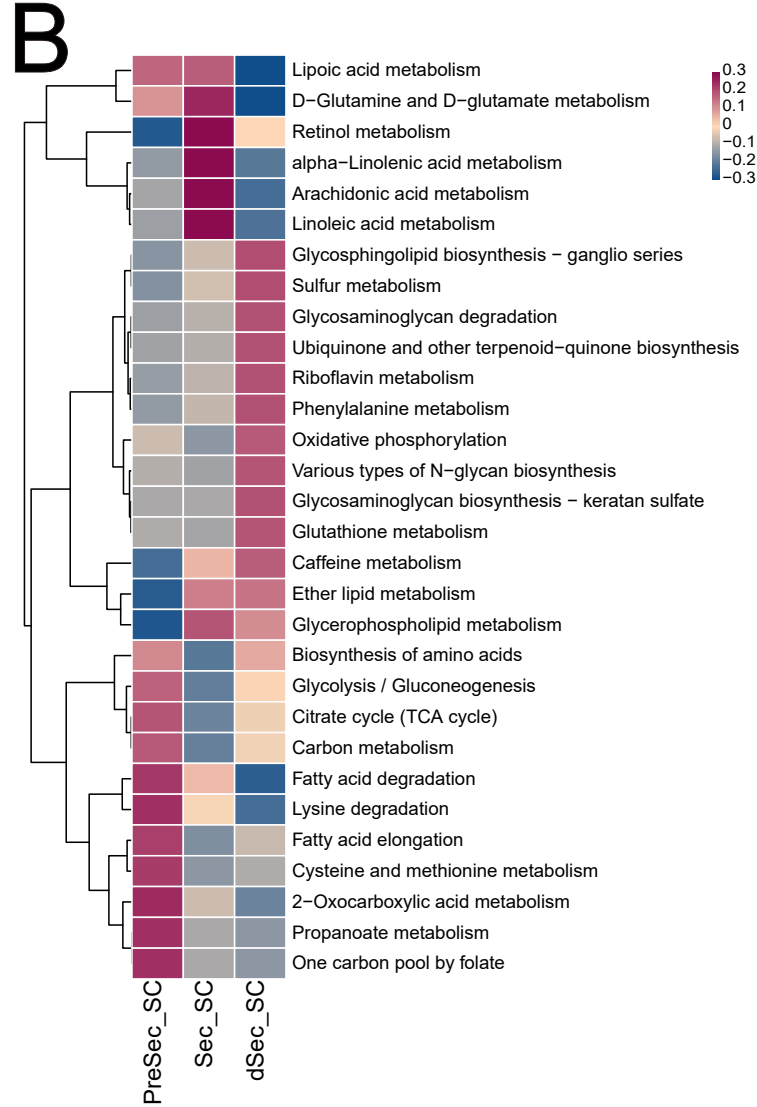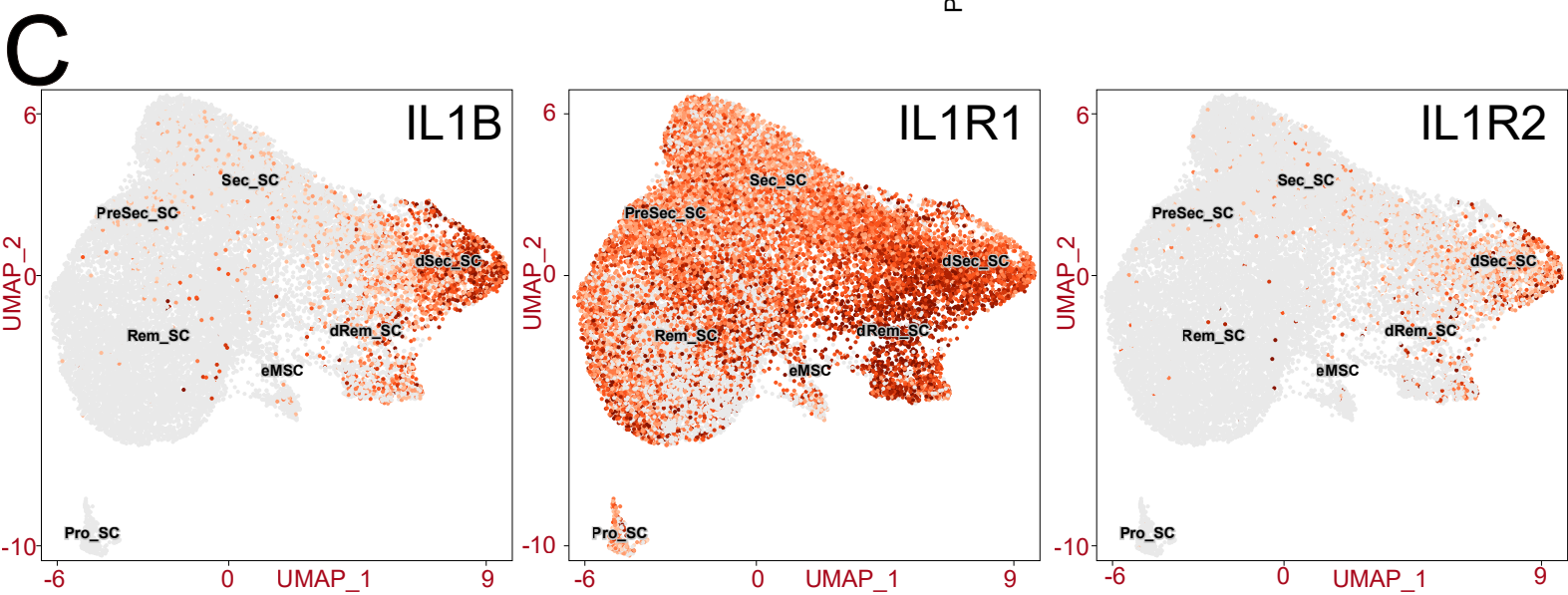

Supplement: Supplementary file 10 — Additional file 10. [file 12915_2022_1483_MOESM10_ESM.pdf]

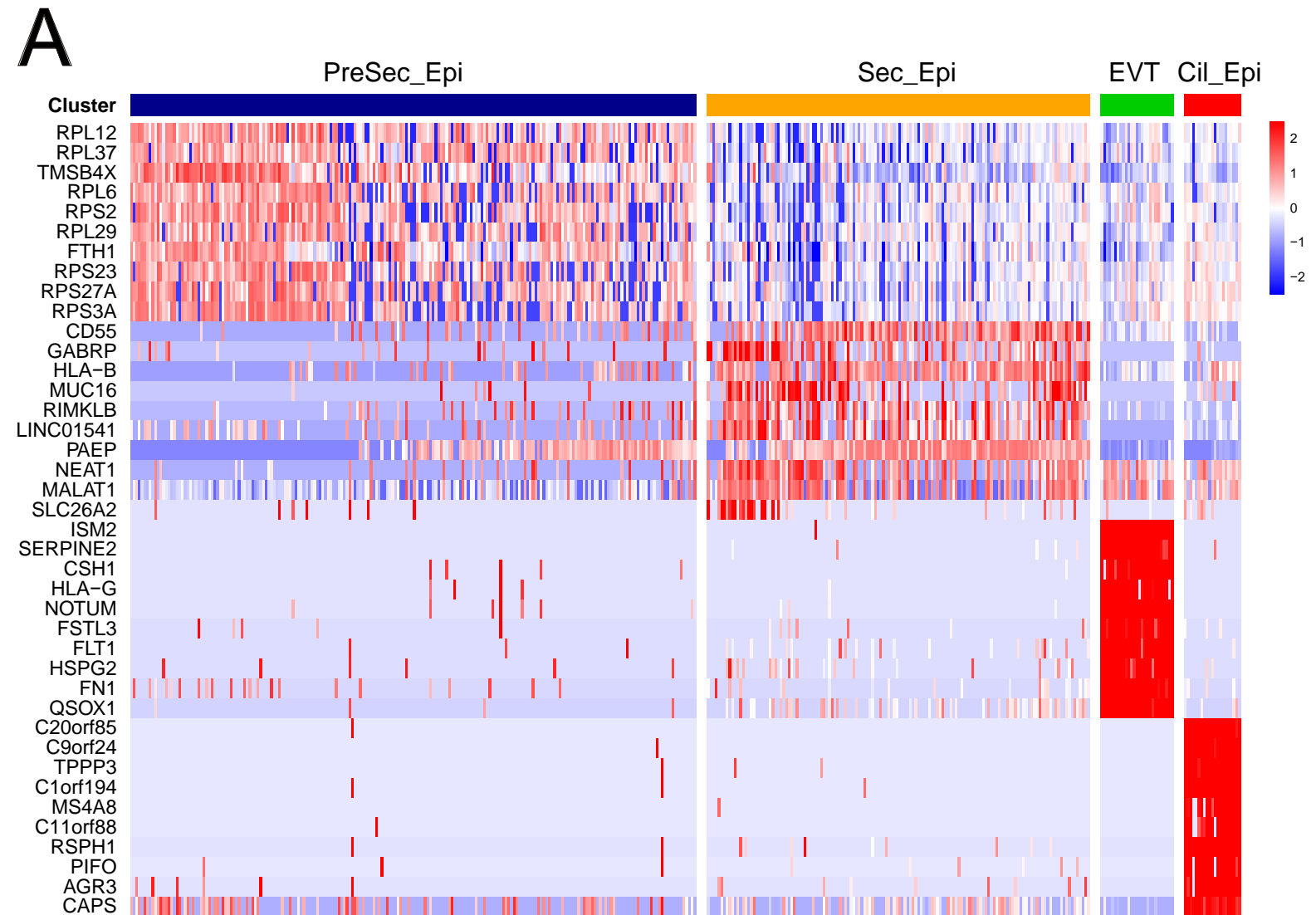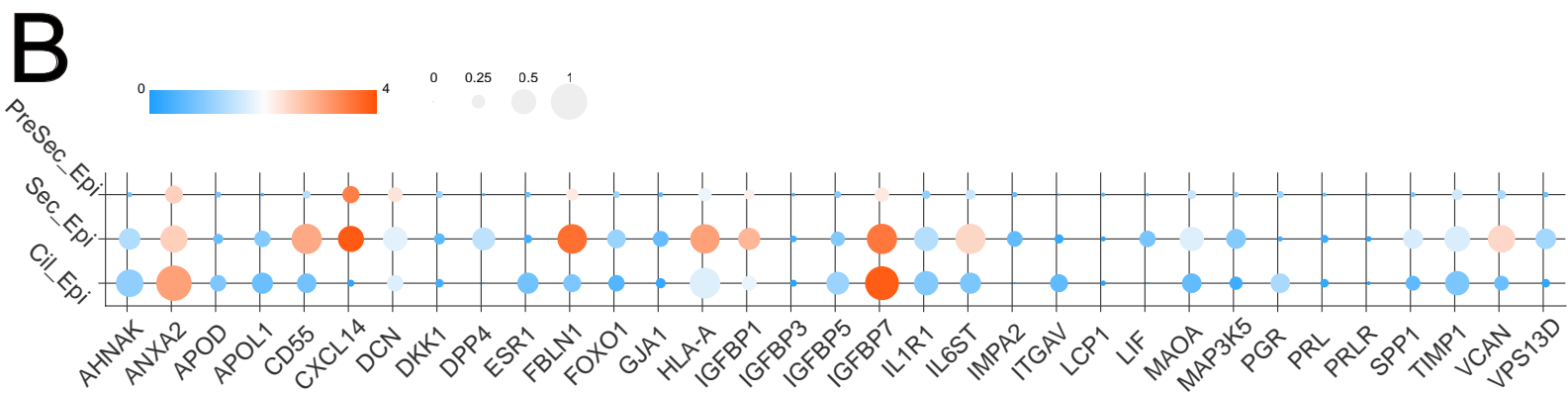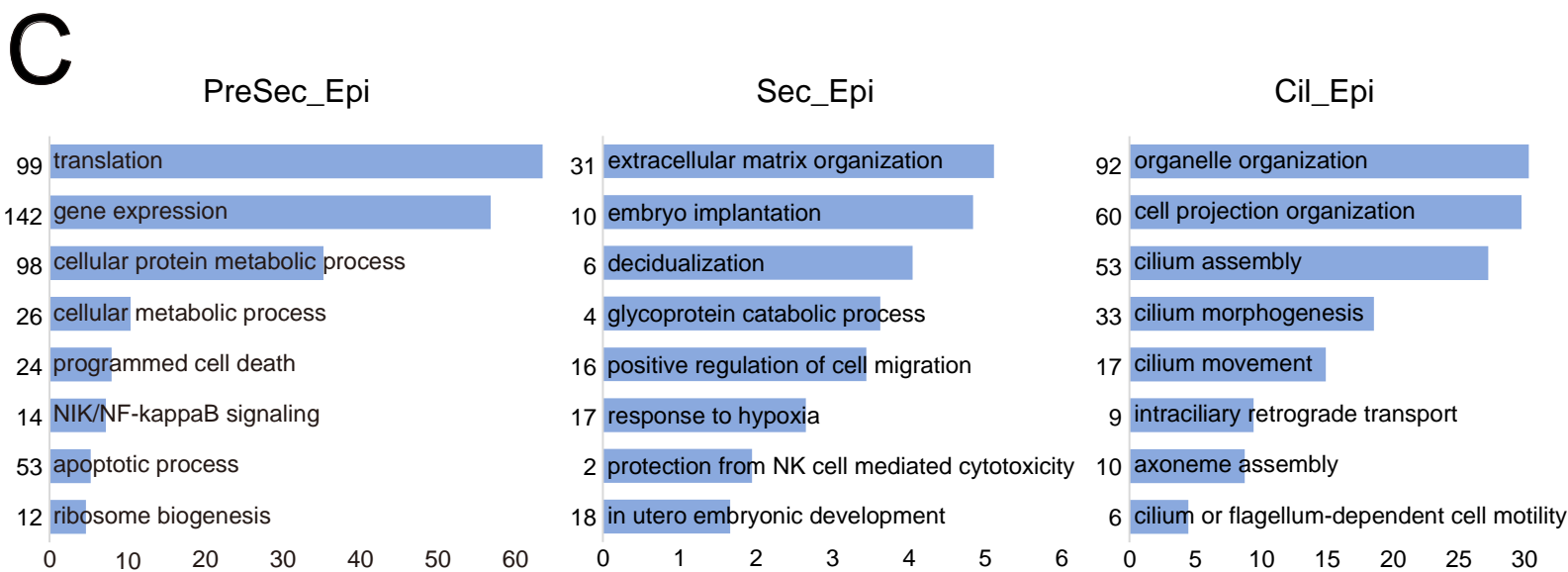

Supplement: Supplementary file 11 — Additional file 11. [file 12915_2022_1483_MOESM11_ESM.pdf]

A

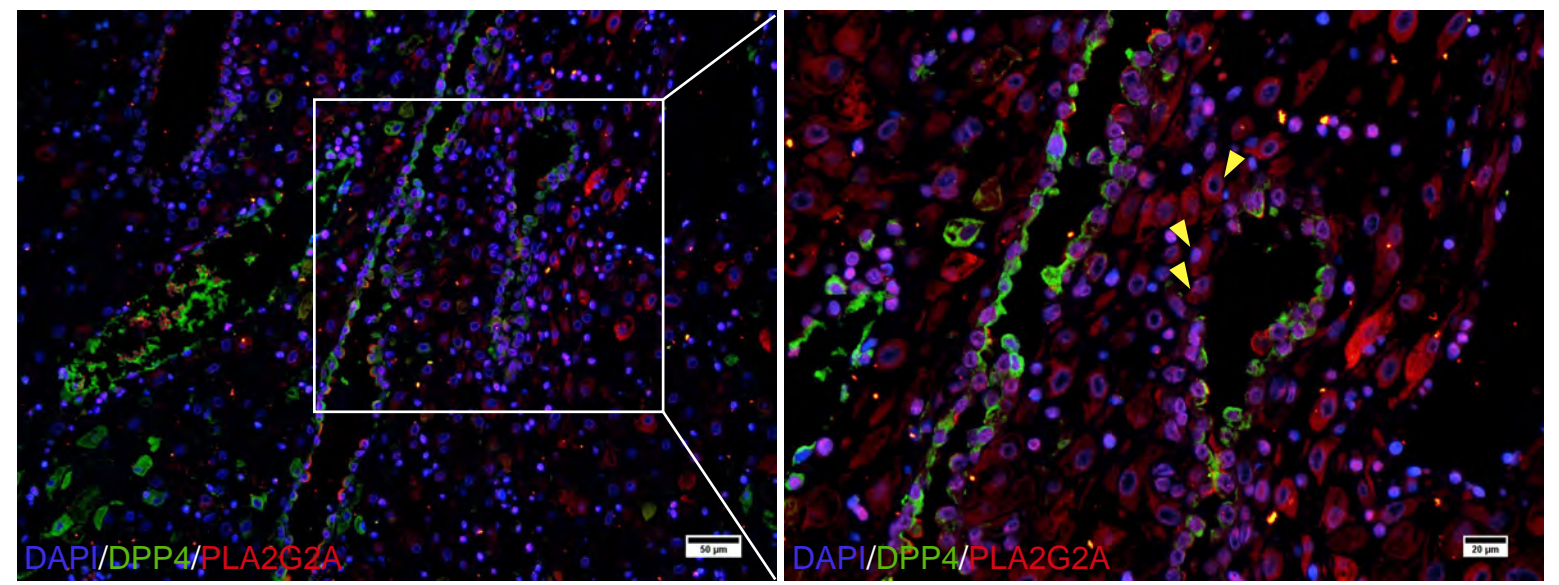

B

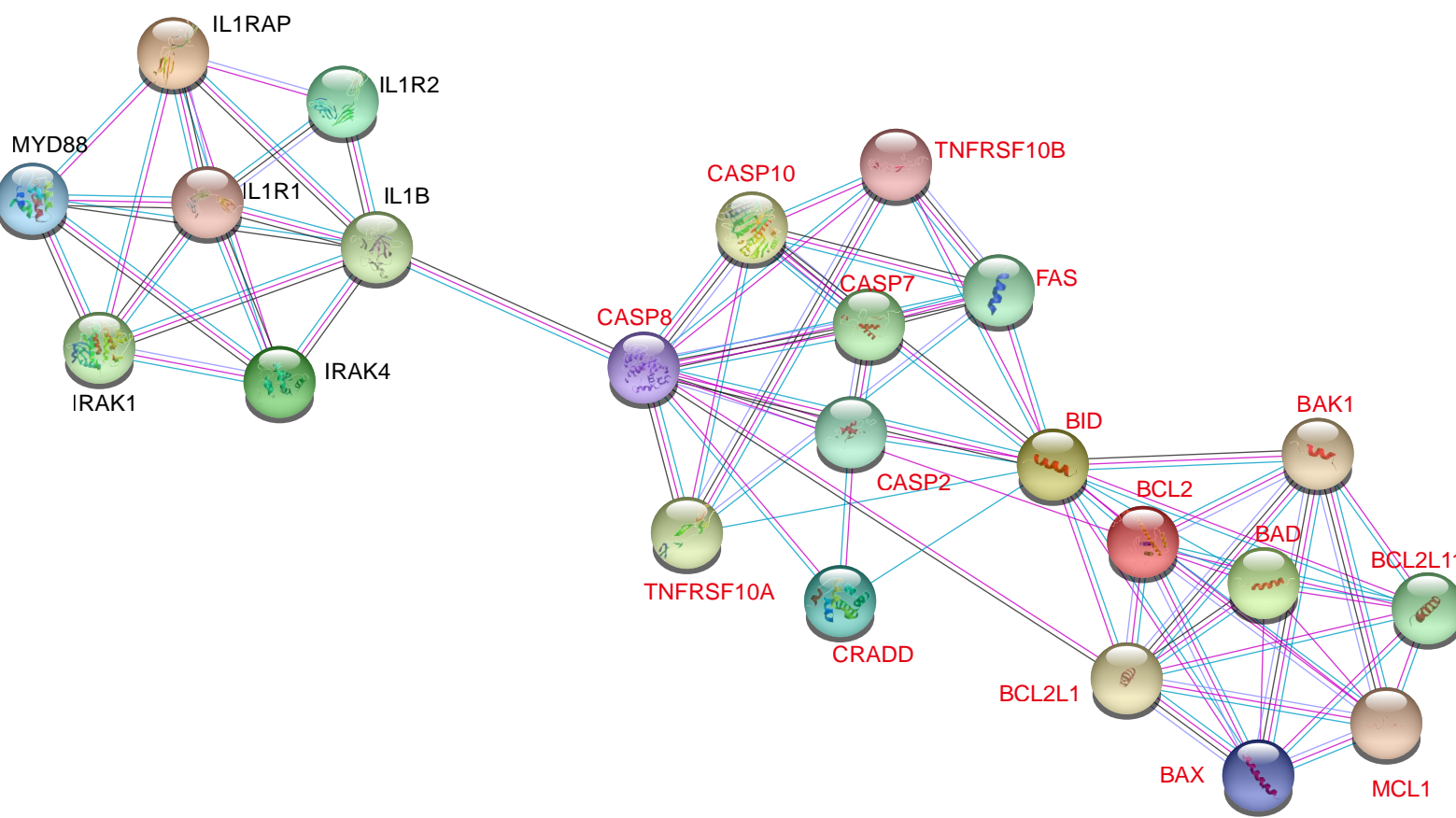

Supplement: Supplementary file 12 — Additional file 12. [file 12915_2022_1483_MOESM12_ESM.pdf]

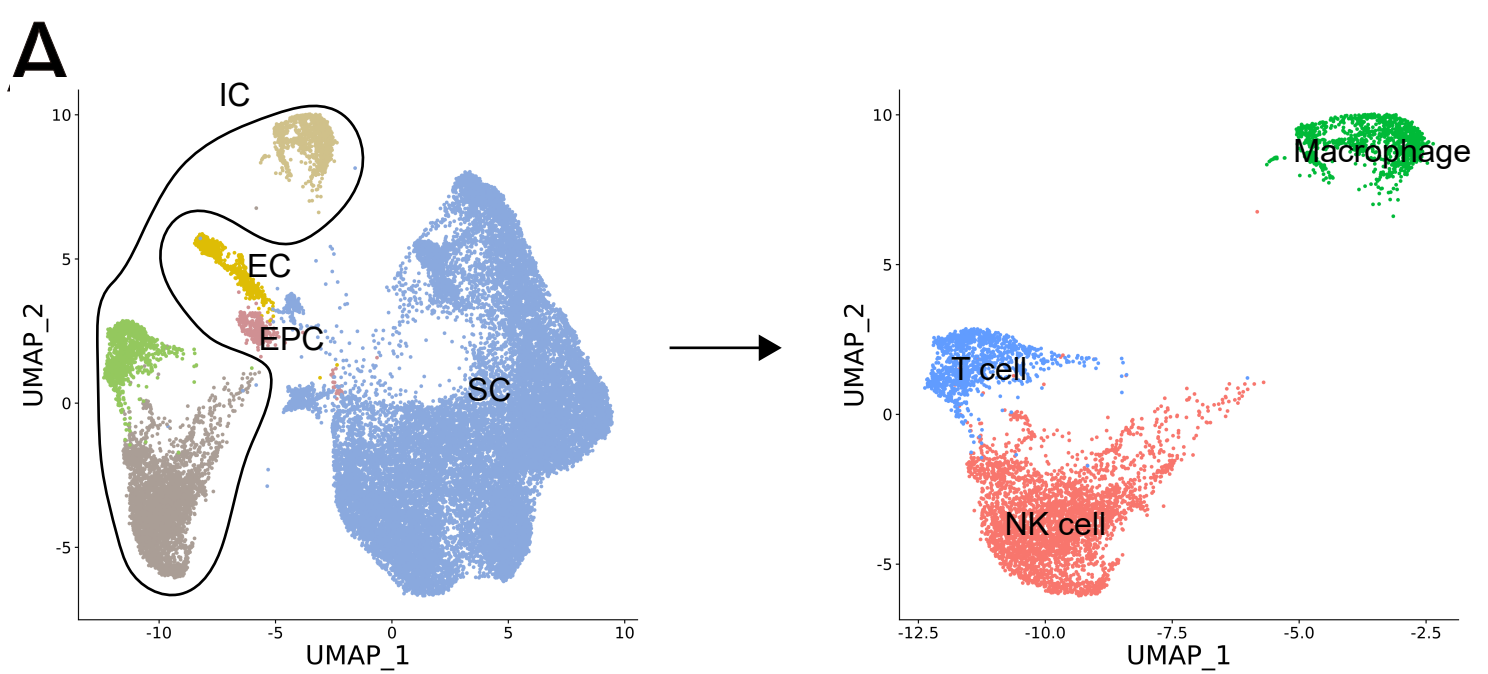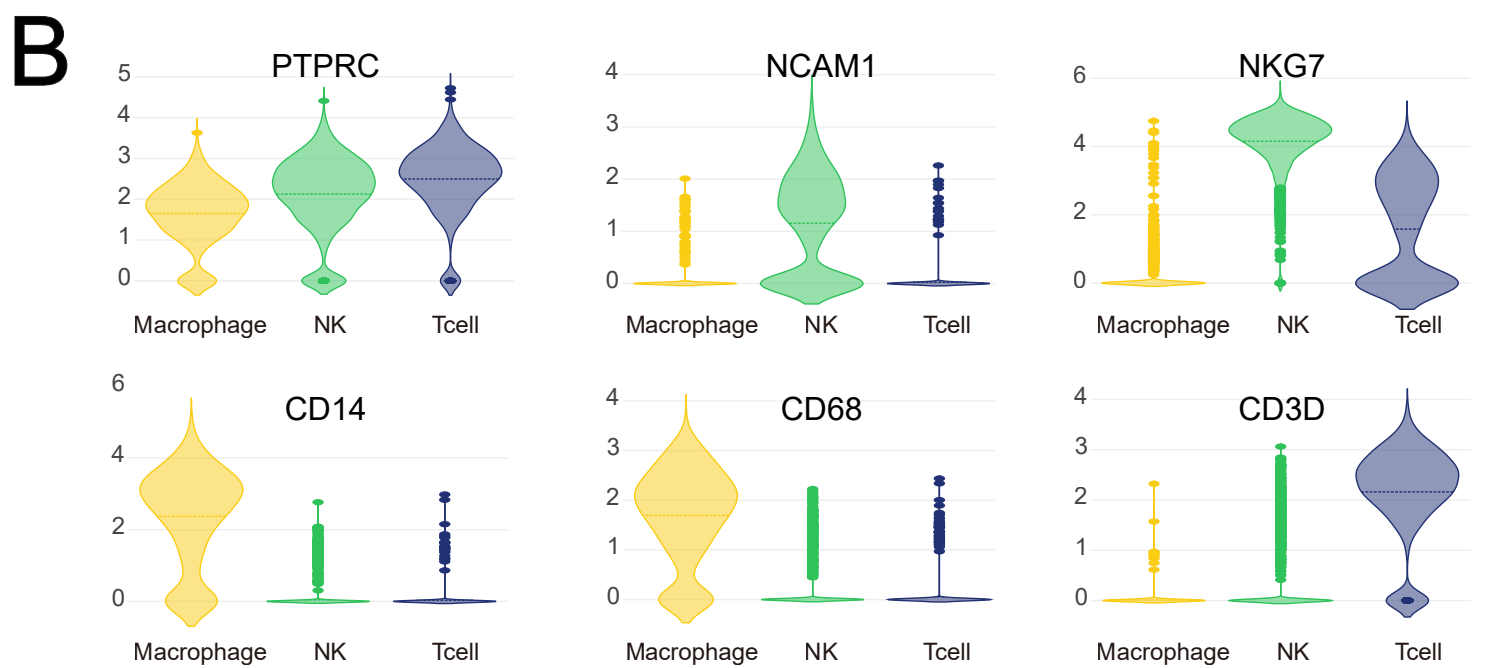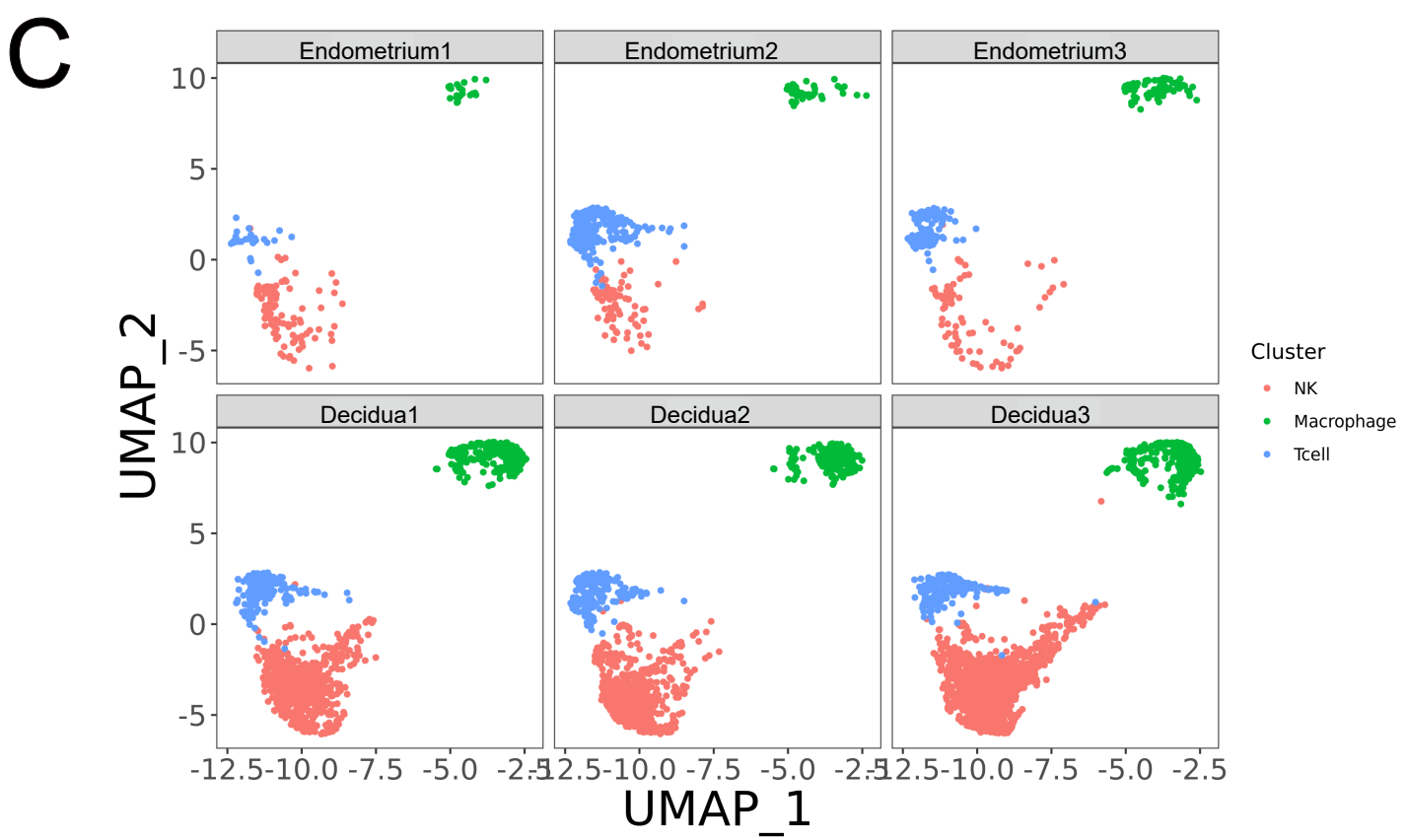

Supplement: Supplementary file 13 — Additional file 13. [file 12915_2022_1483_MOESM13_ESM.pdf]

Endometrium  
endometrial cell

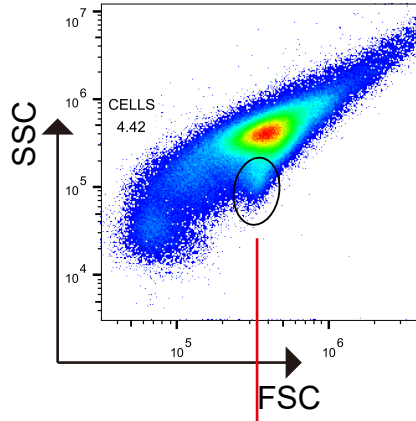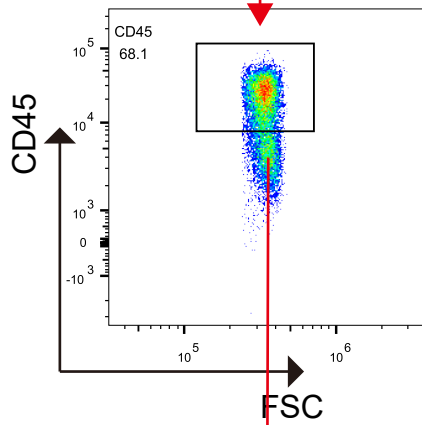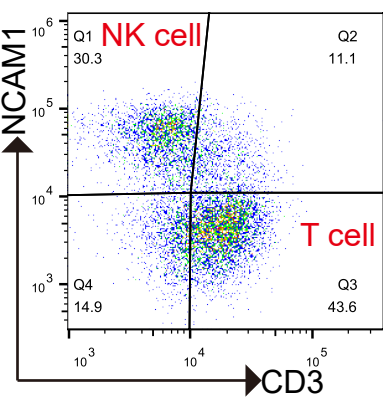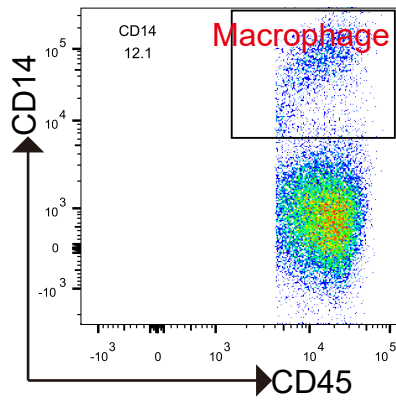

Decidua  
decidual immune cell

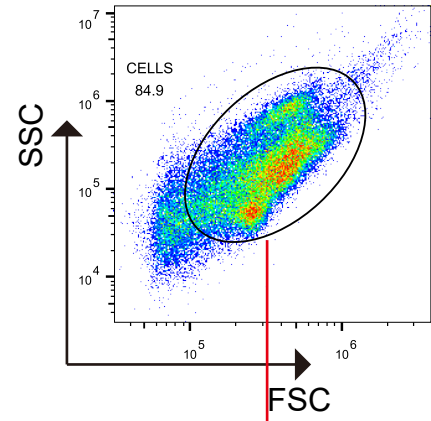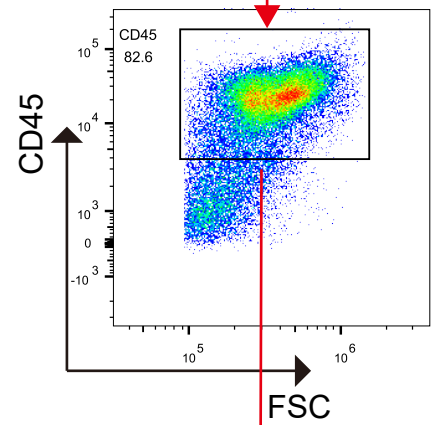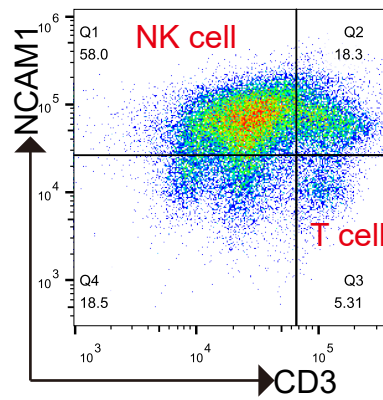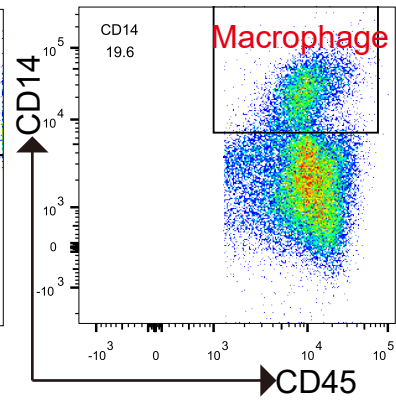

Supplement: Supplementary file 14 — Additional file 14. [file 12915_2022_1483_MOESM14_ESM.pdf]

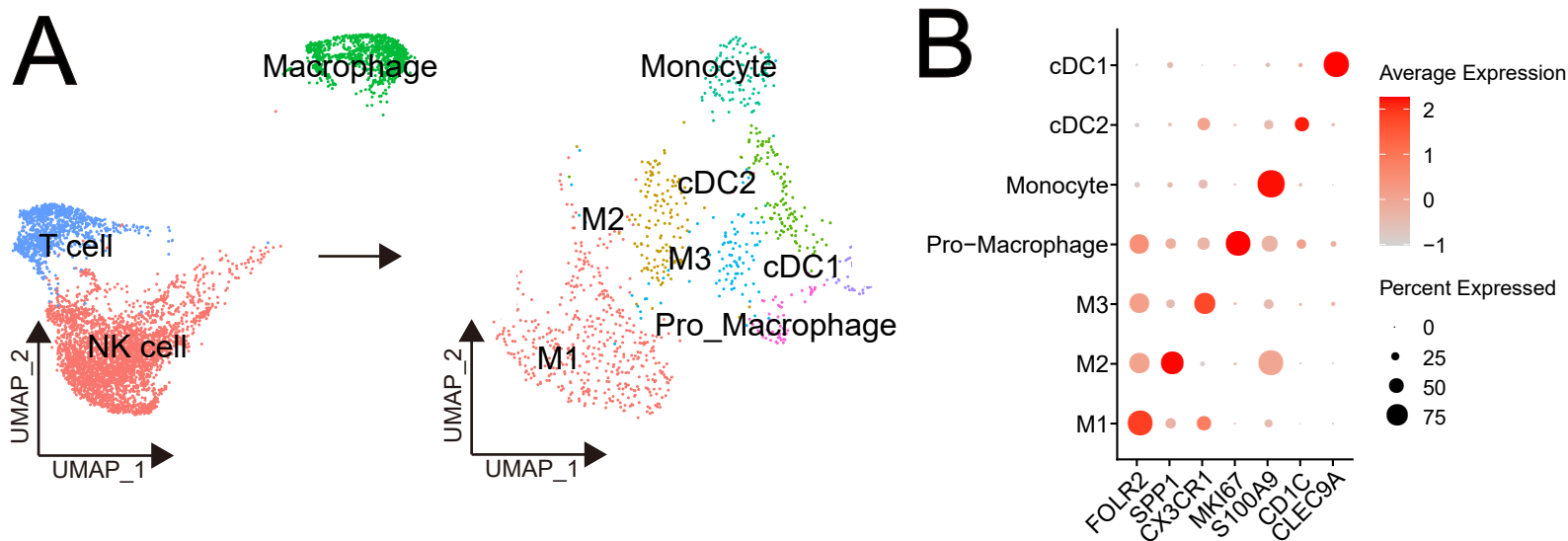

## C

### Endometrium

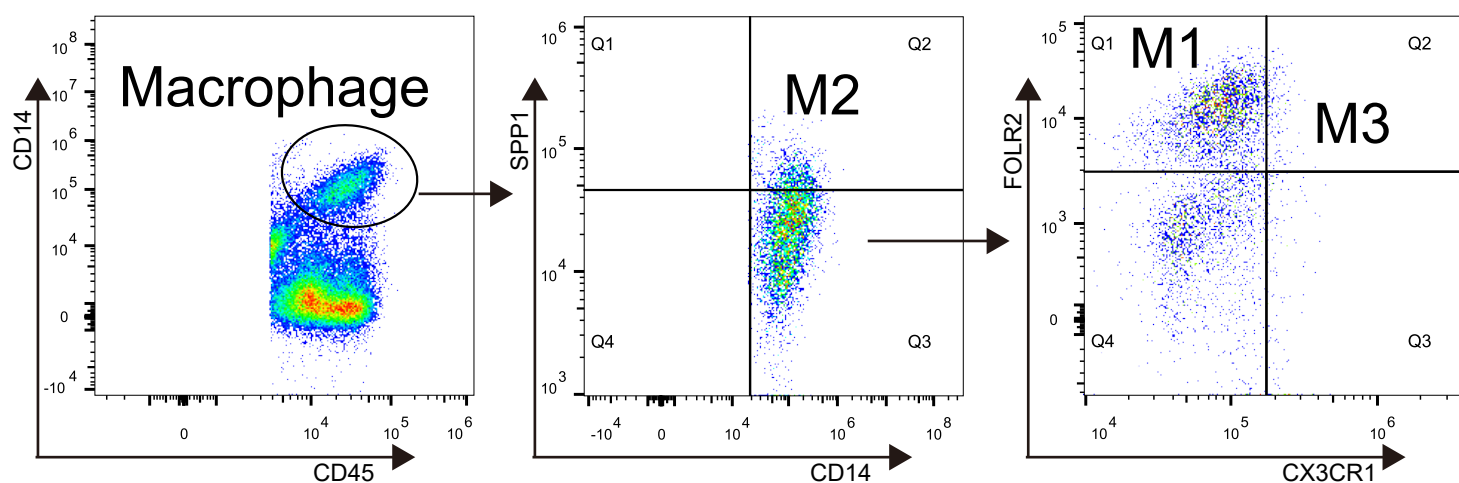

### Decidua

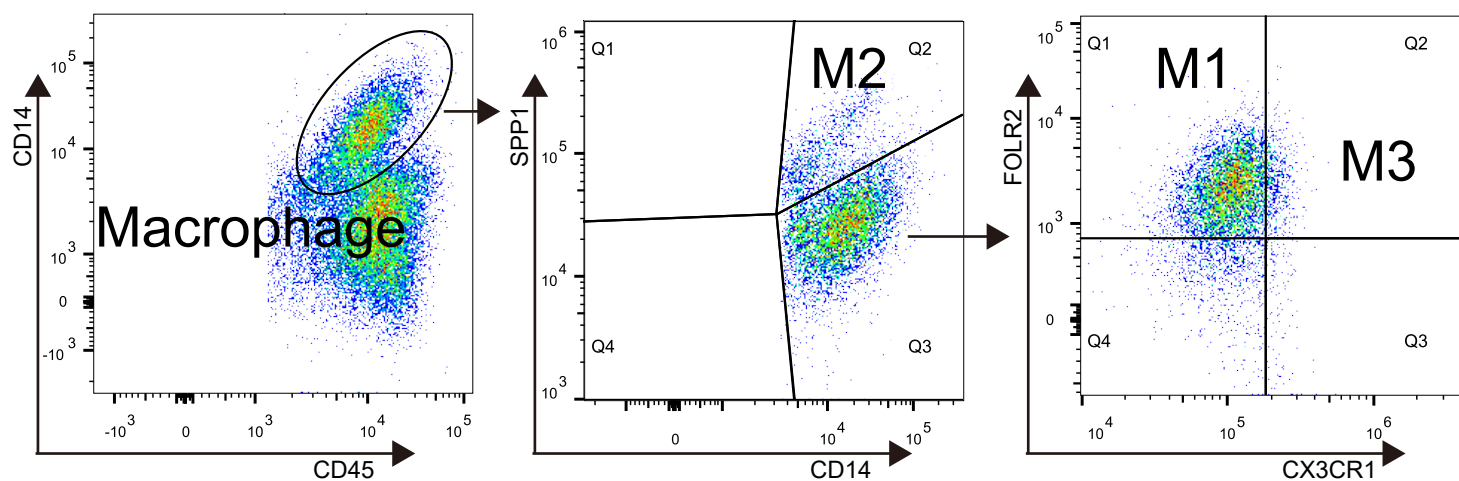

Supplement: Supplementary file 15 — Additional file 15. [file 12915_2022_1483_MOESM15_ESM.pdf]

A

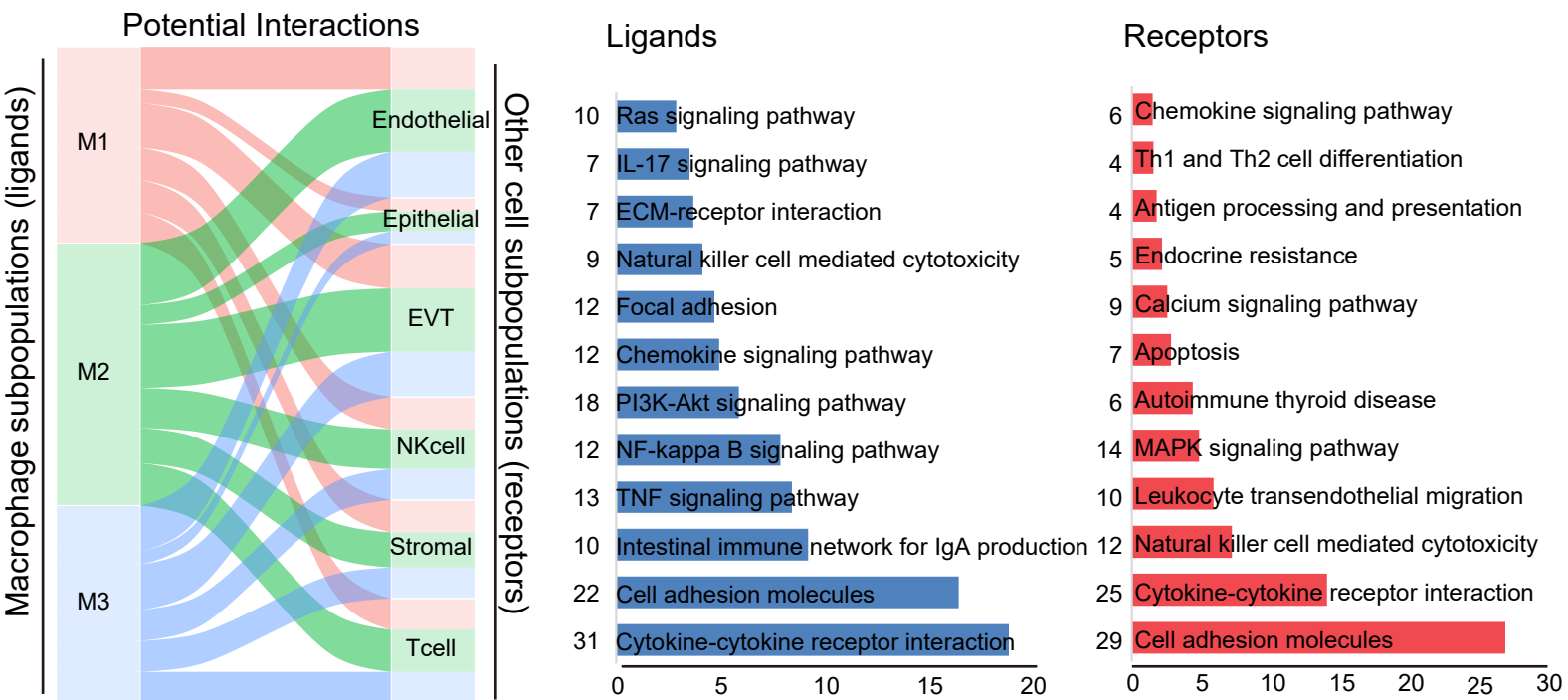

B

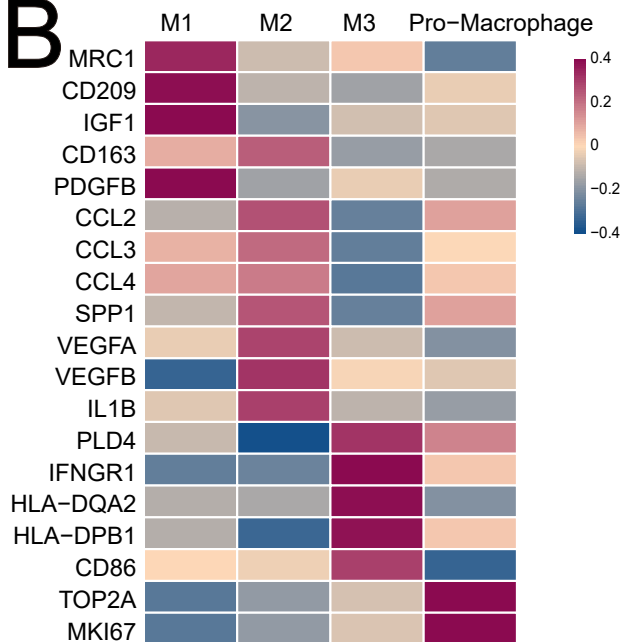

C

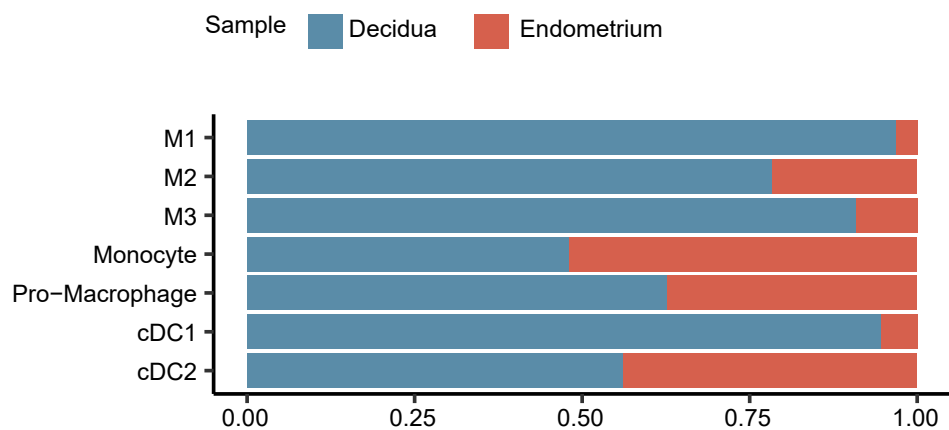

D

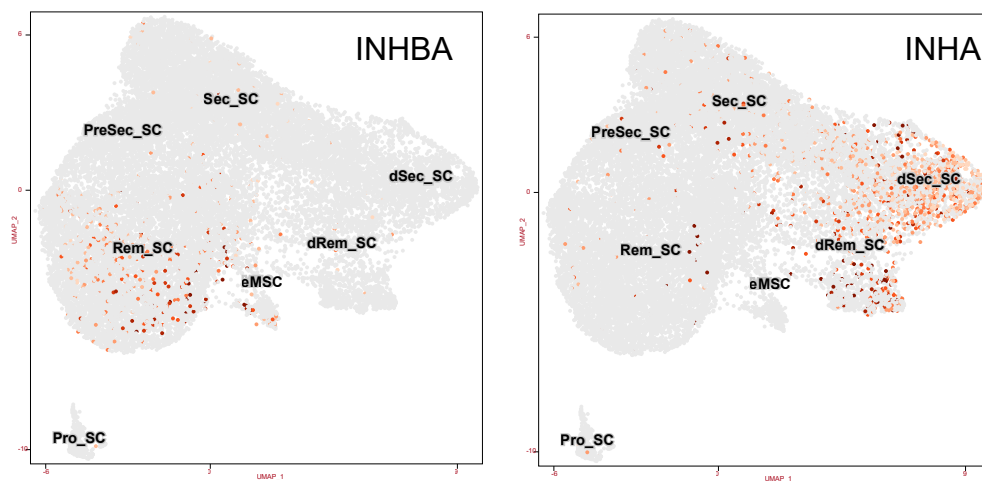

Supplement: Supplementary file 16 — Additional file 16. [file 12915_2022_1483_MOESM16_ESM.pdf]

# A Endometrium

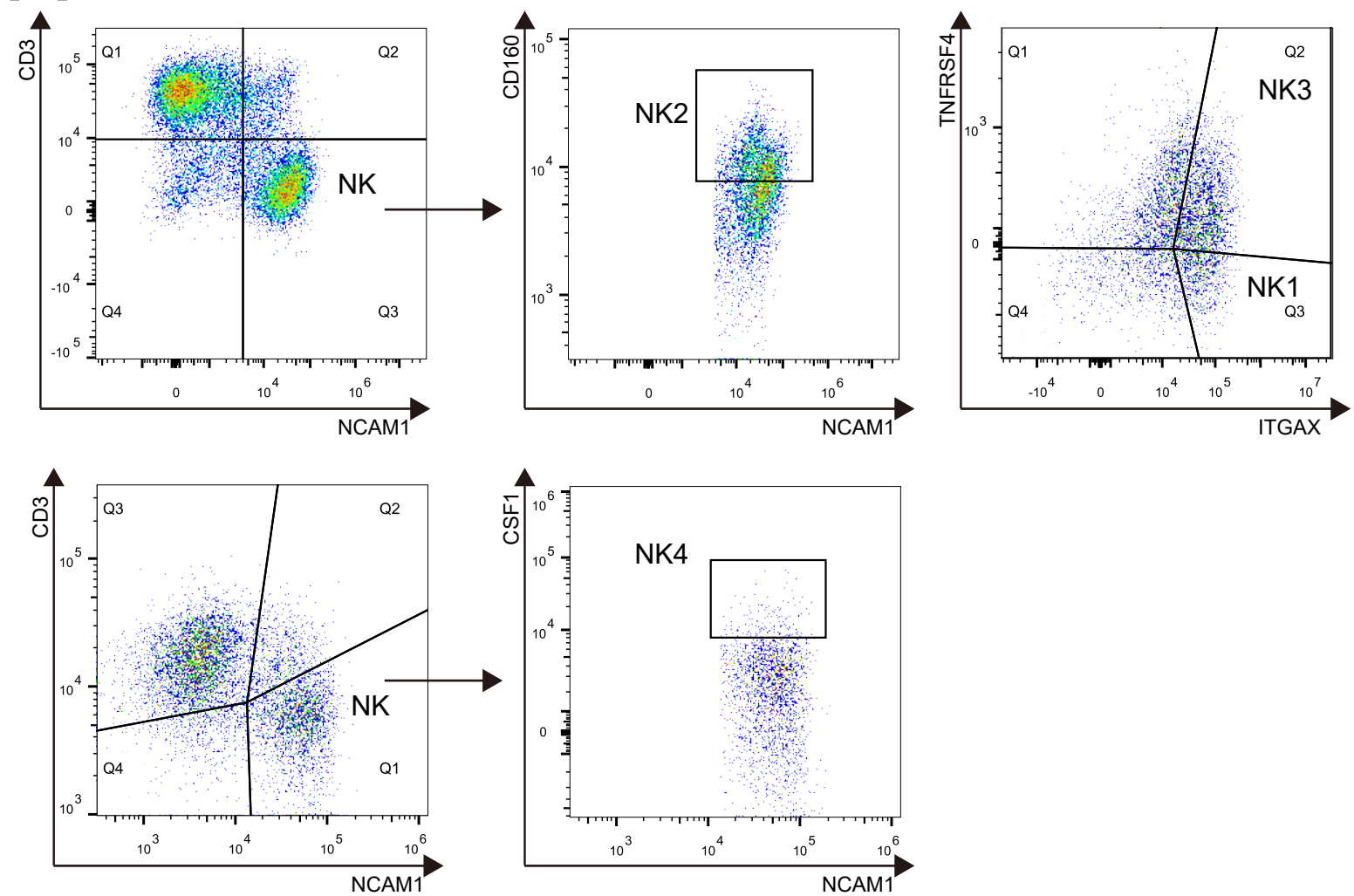

# B Decidua

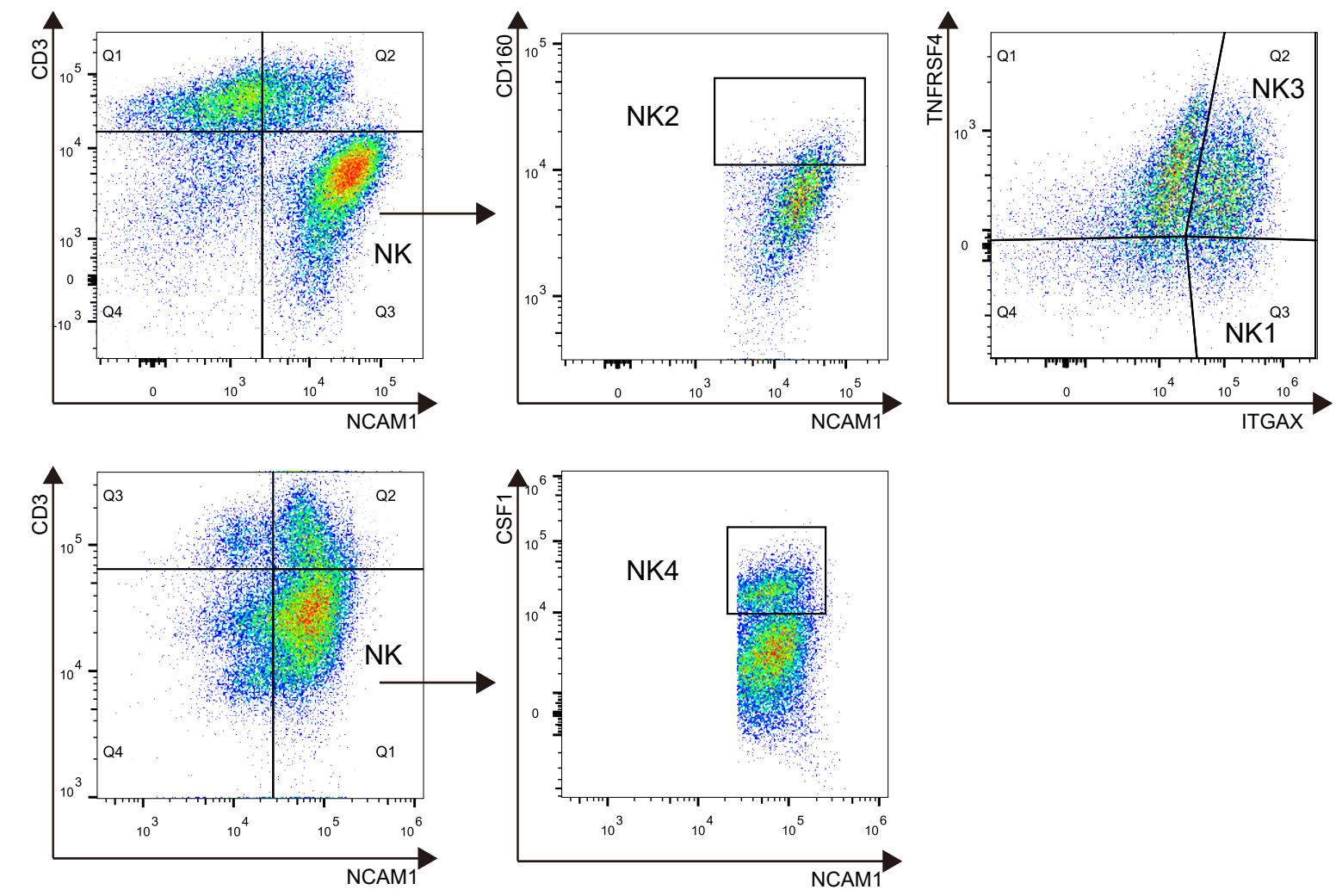

Supplement: Supplementary file 17 — Additional file 17. [file 12915_2022_1483_MOESM17_ESM.pdf]

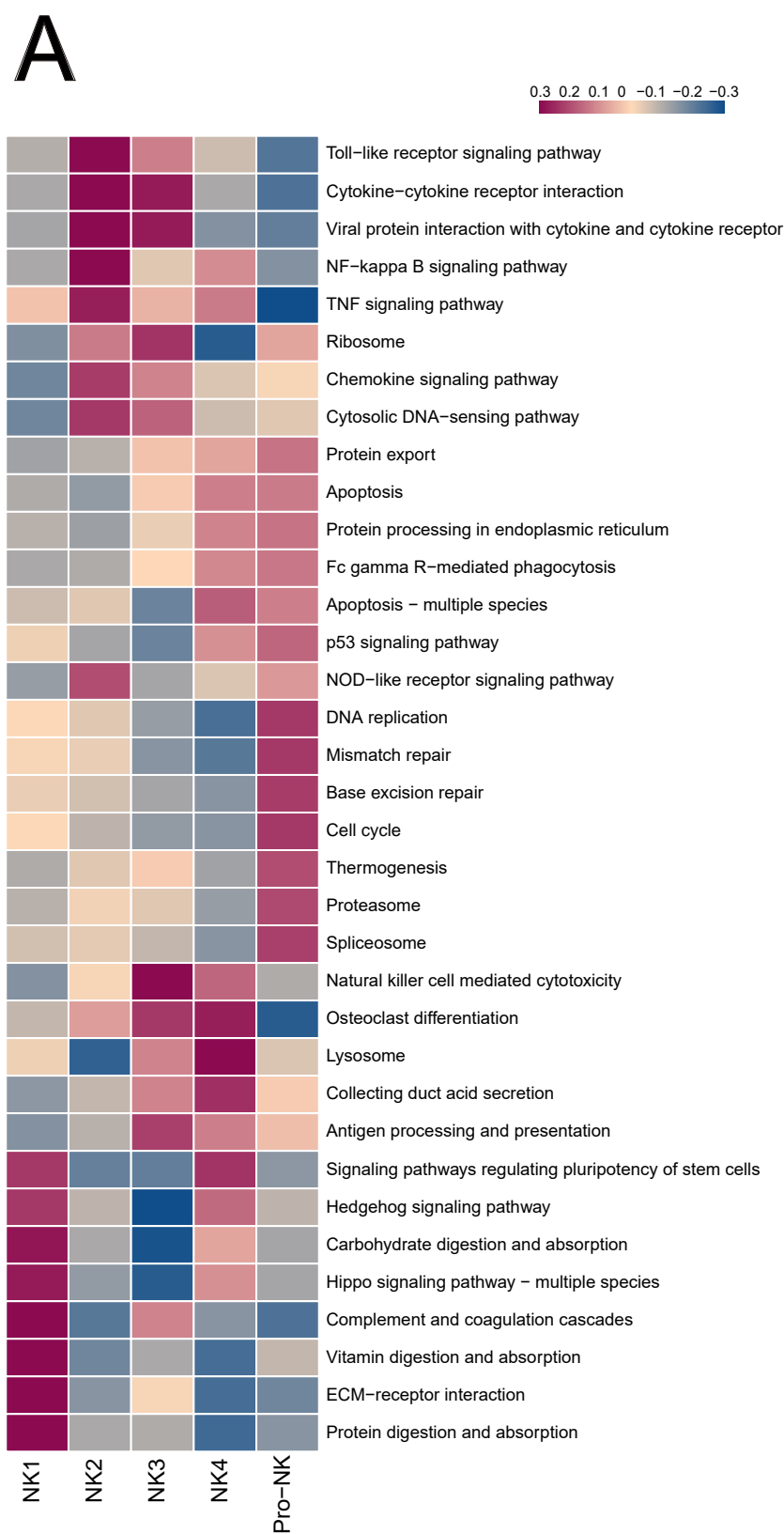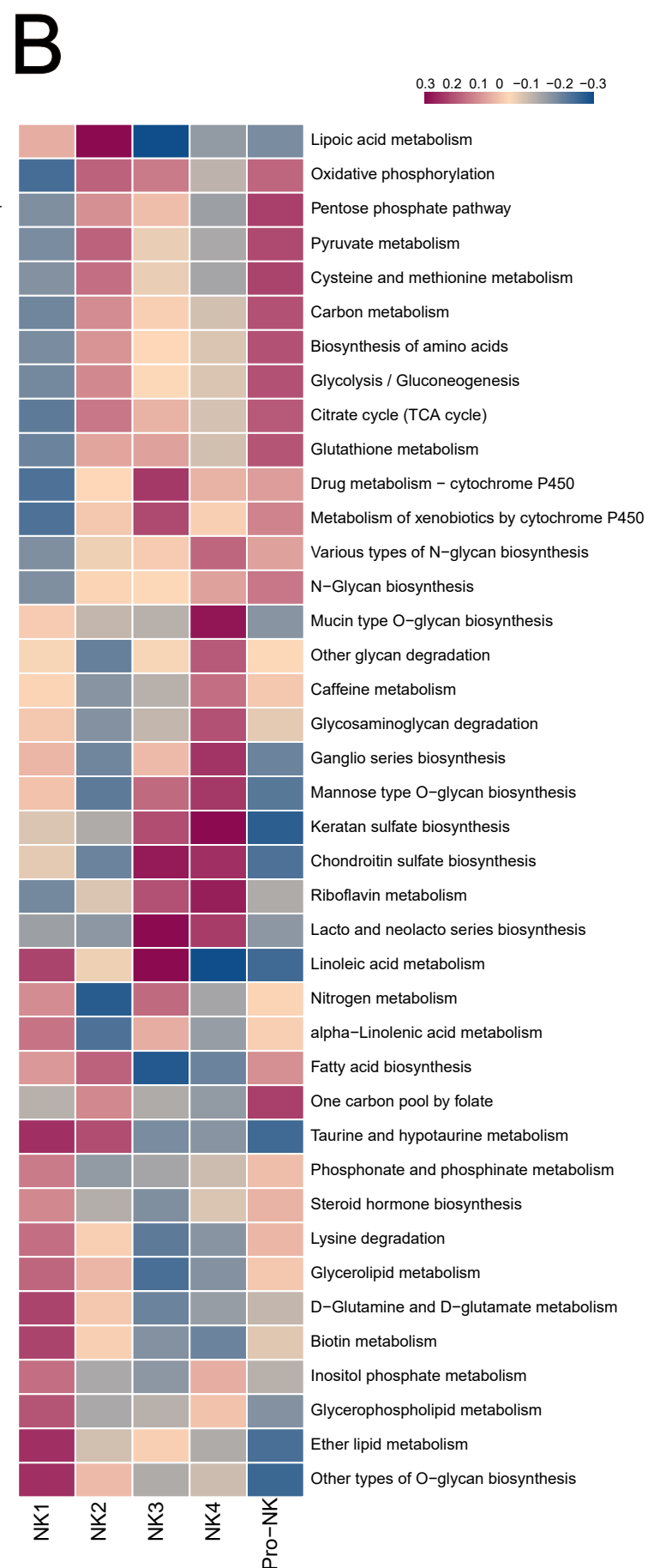

Supplement: Supplementary file 18 — Additional file 18. [file 12915_2022_1483_MOESM18_ESM.pdf]

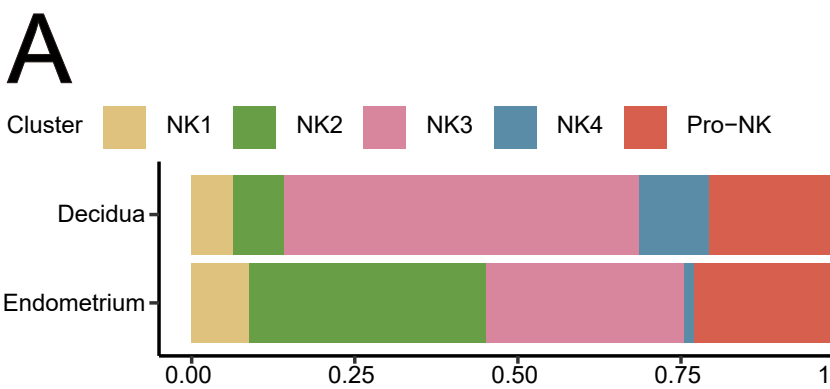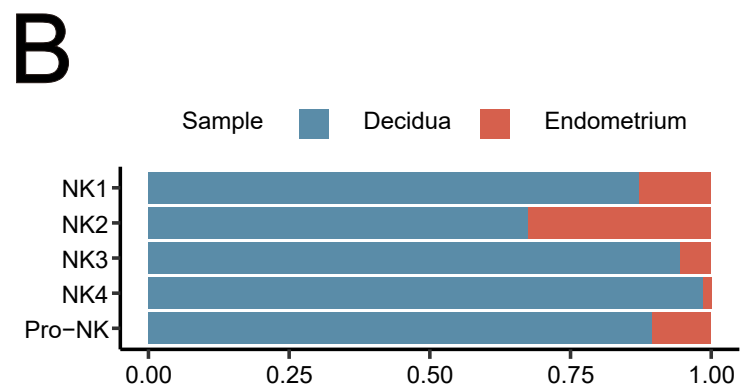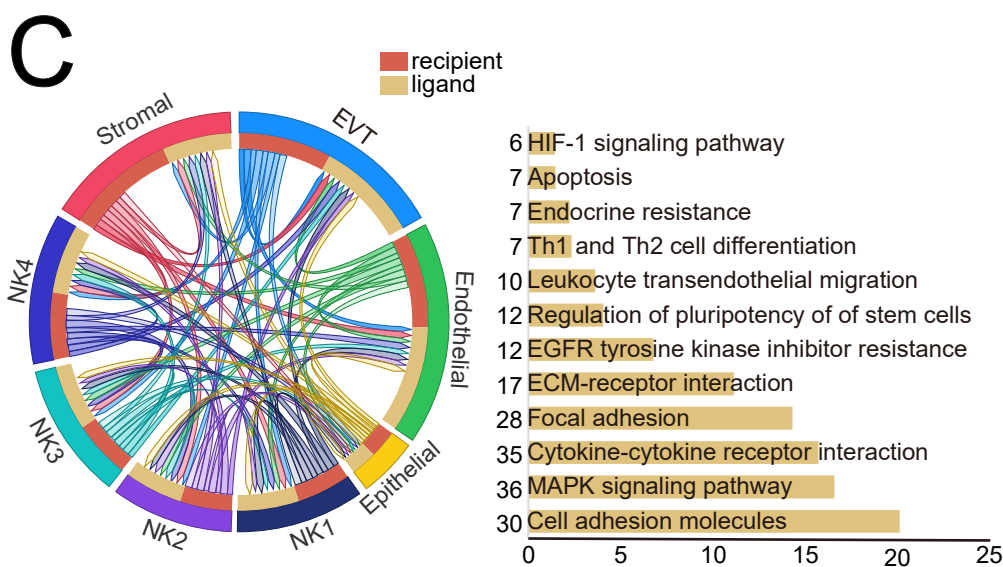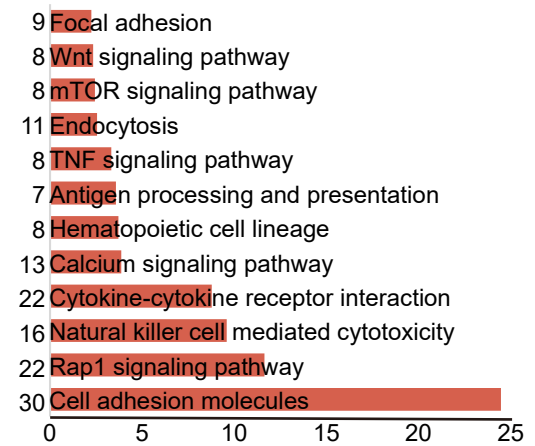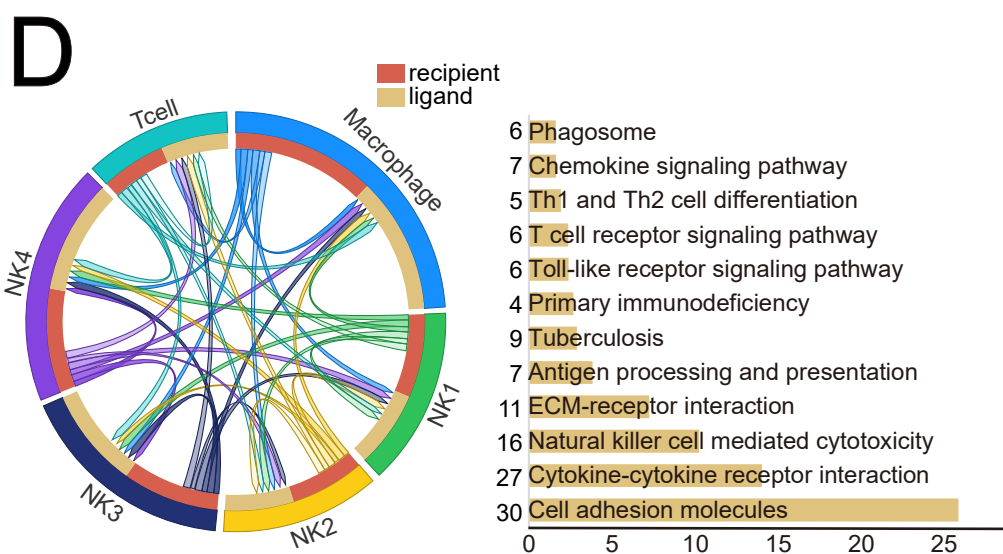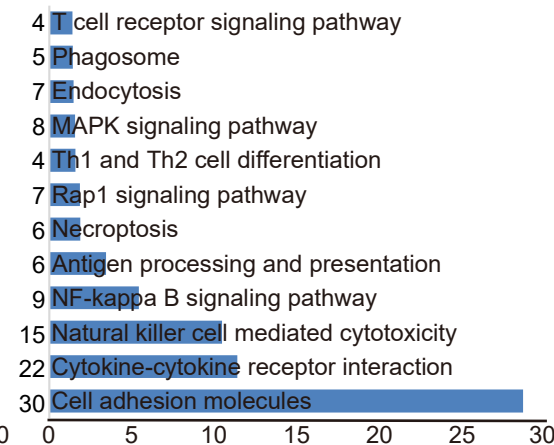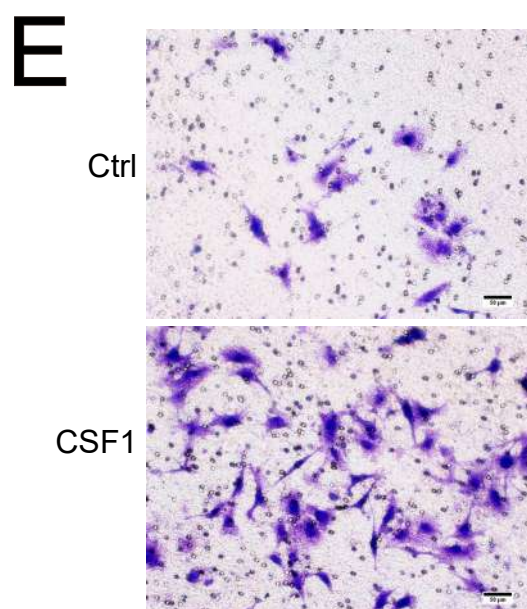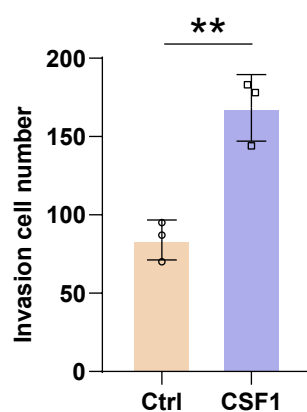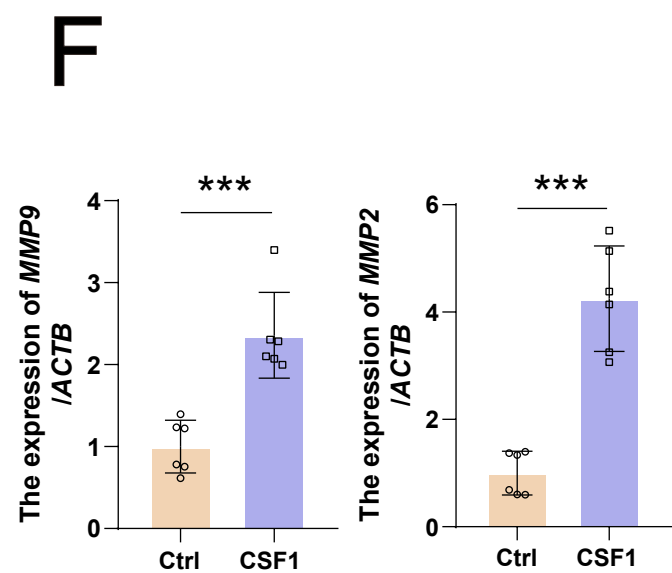

Supplement: Supplementary file 19 — Additional file 19. [file 12915_2022_1483_MOESM19_ESM.pdf]

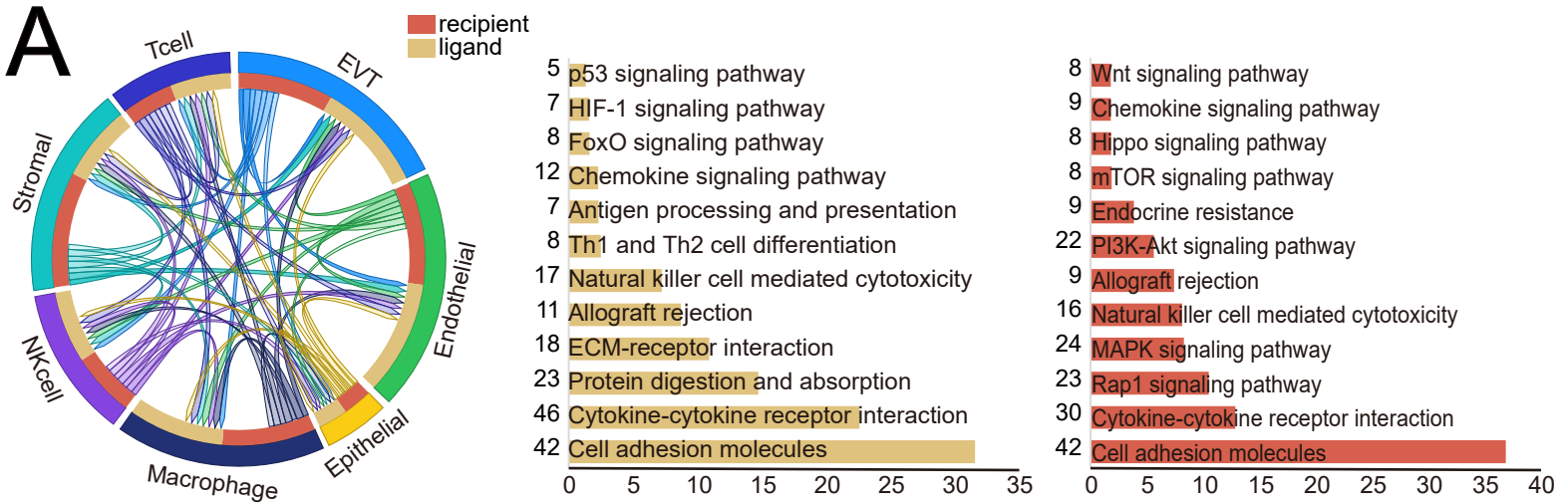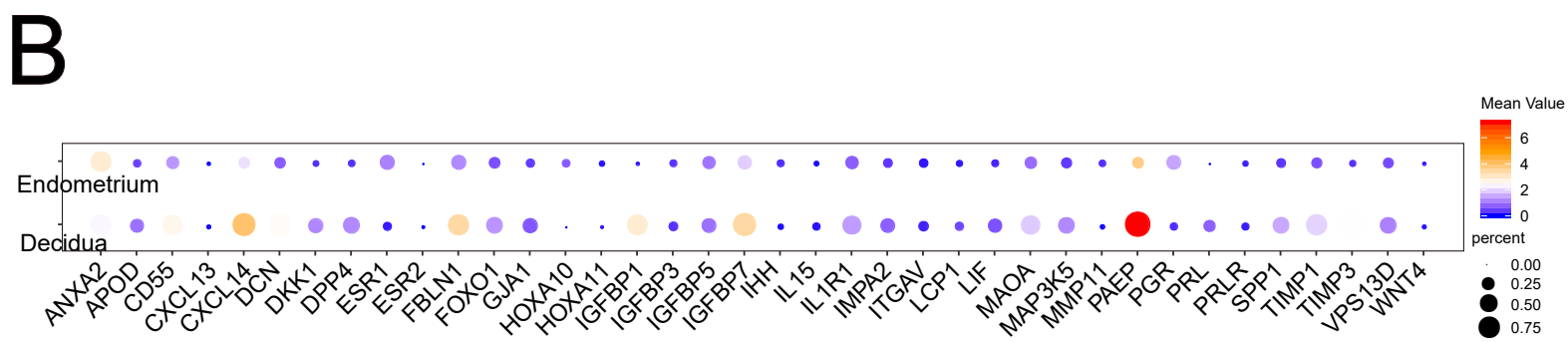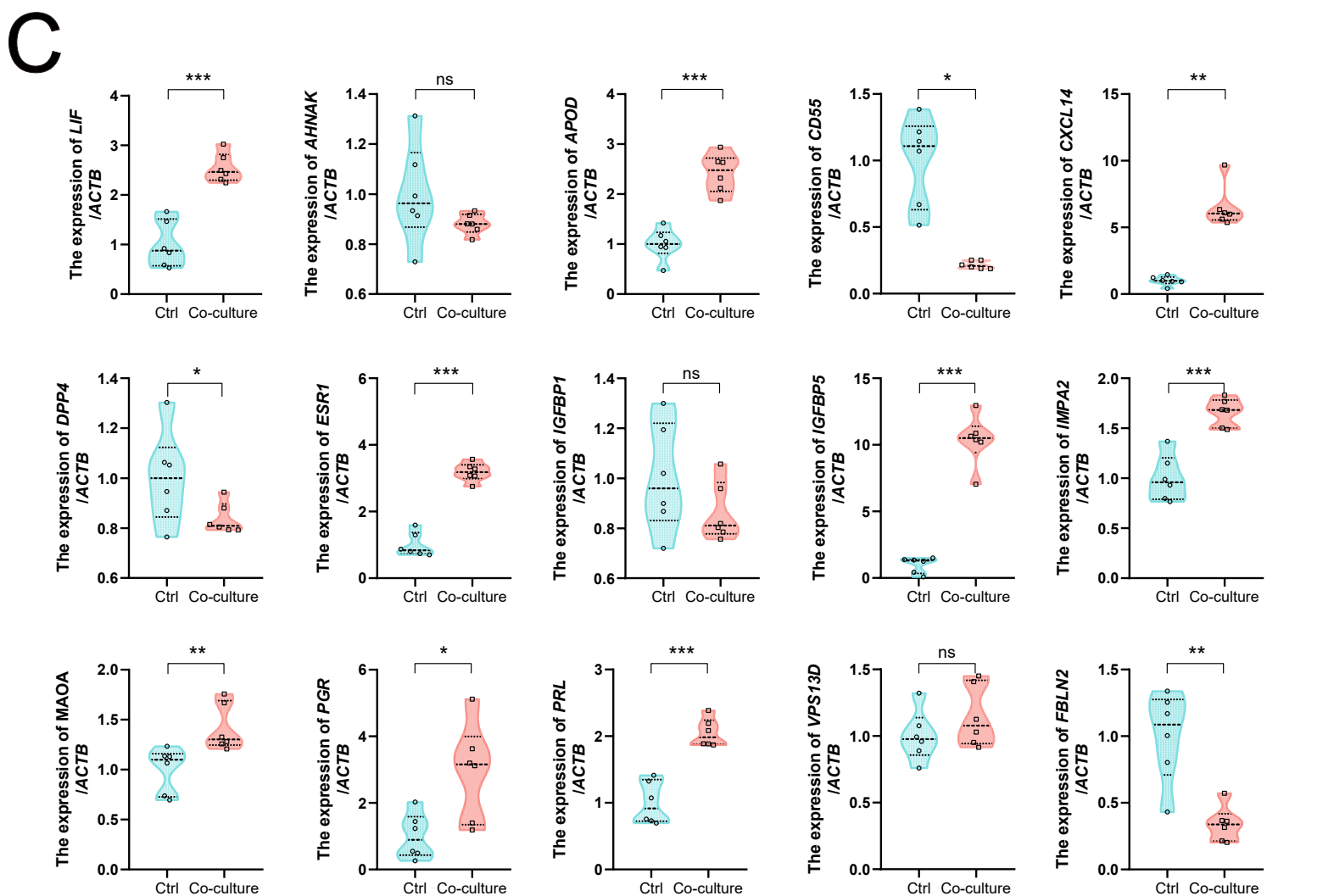

Supplement: Supplementary file 20 — Additional file 20. [file 12915_2022_1483_MOESM20_ESM.pdf]

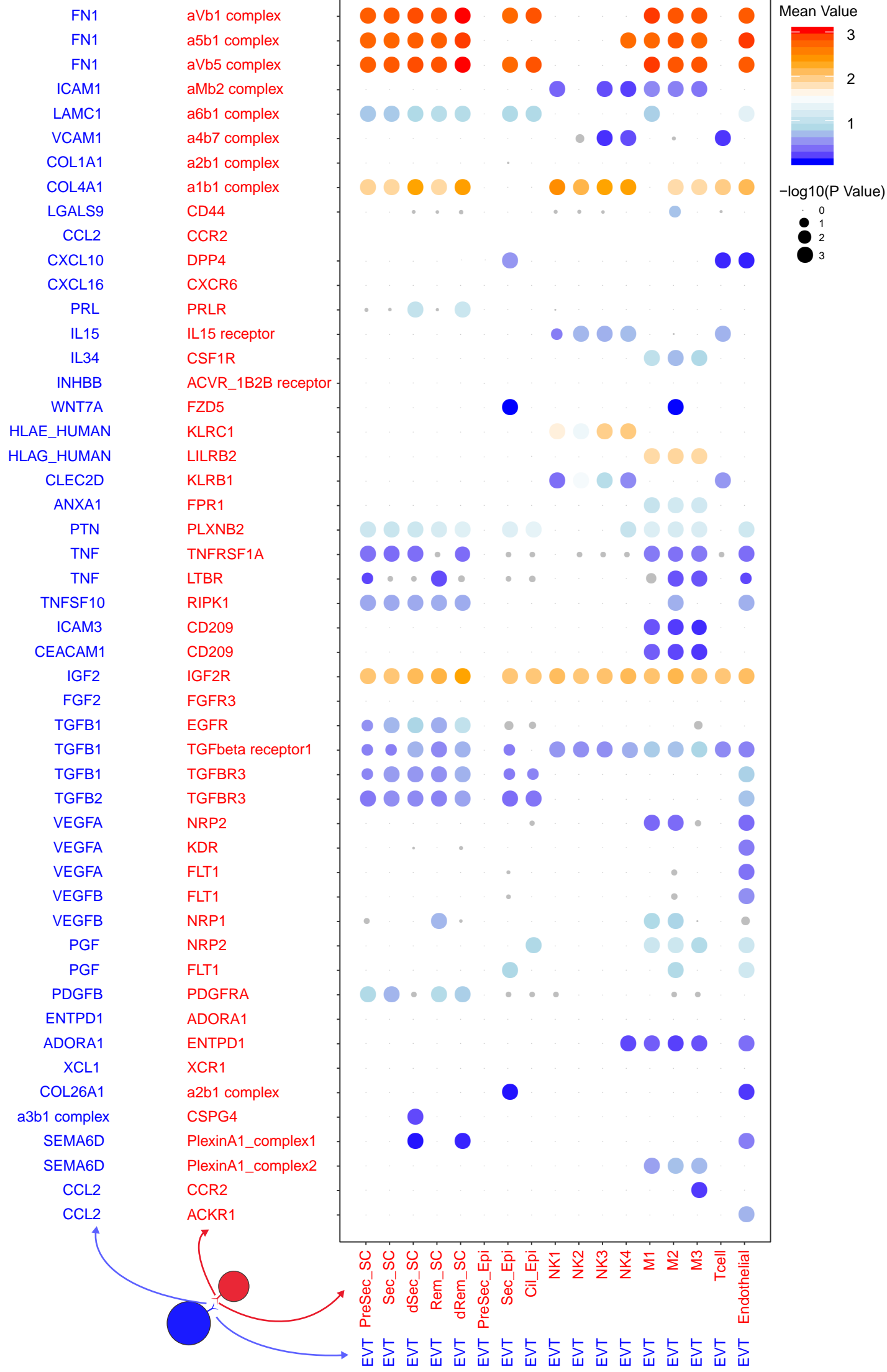

Supplement: Supplementary file 21 — Additional file 21. [file 12915_2022_1483_MOESM21_ESM.pdf]

**A****PRL**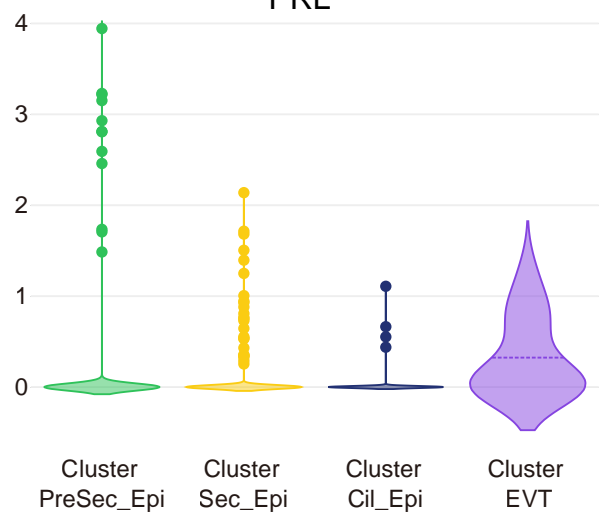**CSH1**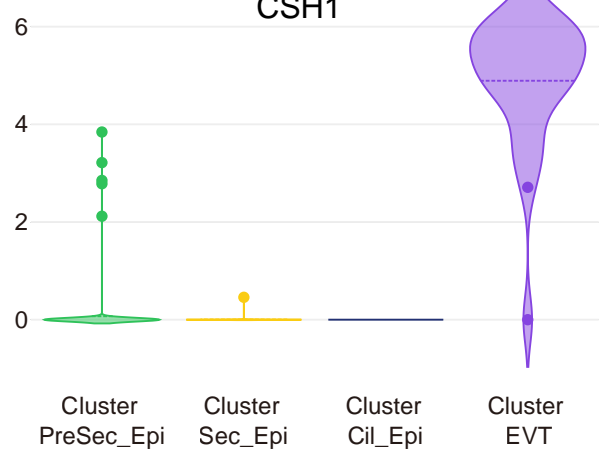**PAPPA**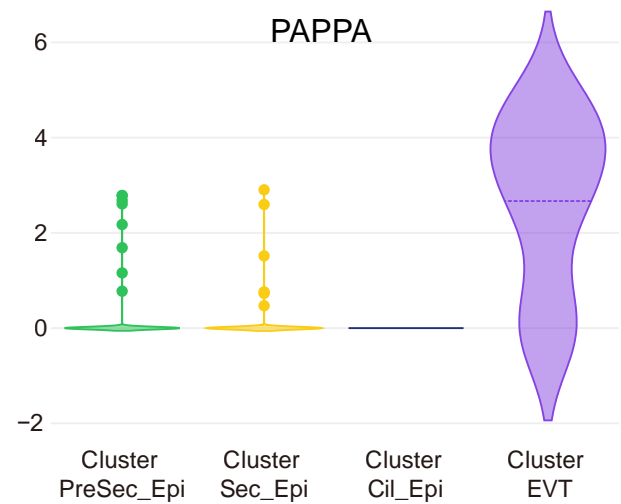**B**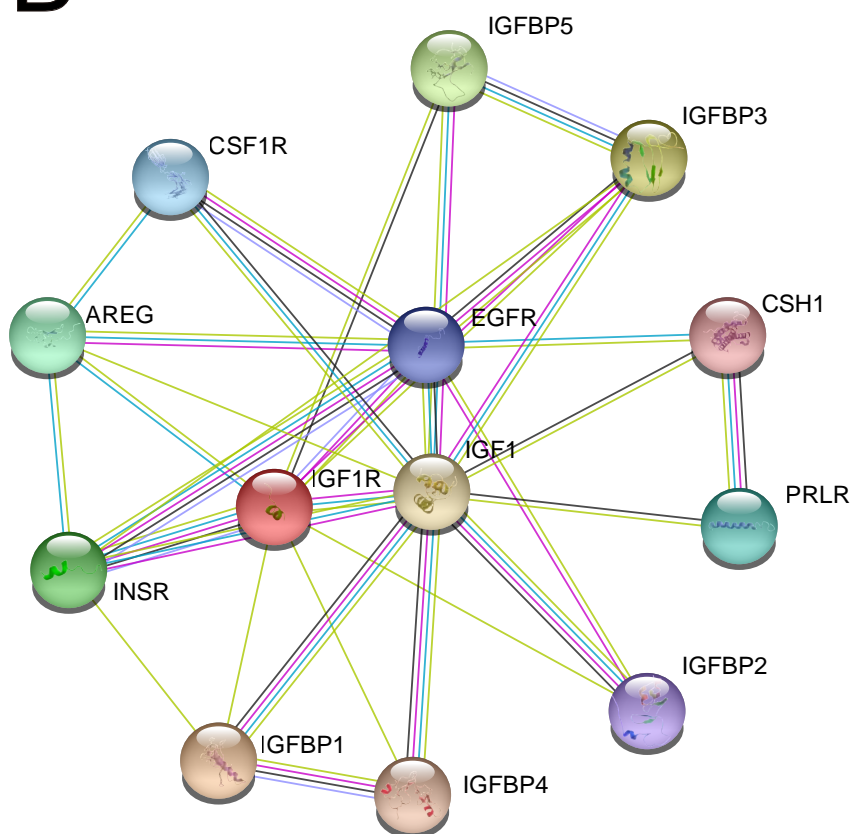

Supplement: Supplementary file 22 — Additional file 22. [file 12915_2022_1483_MOESM22_ESM.pdf]

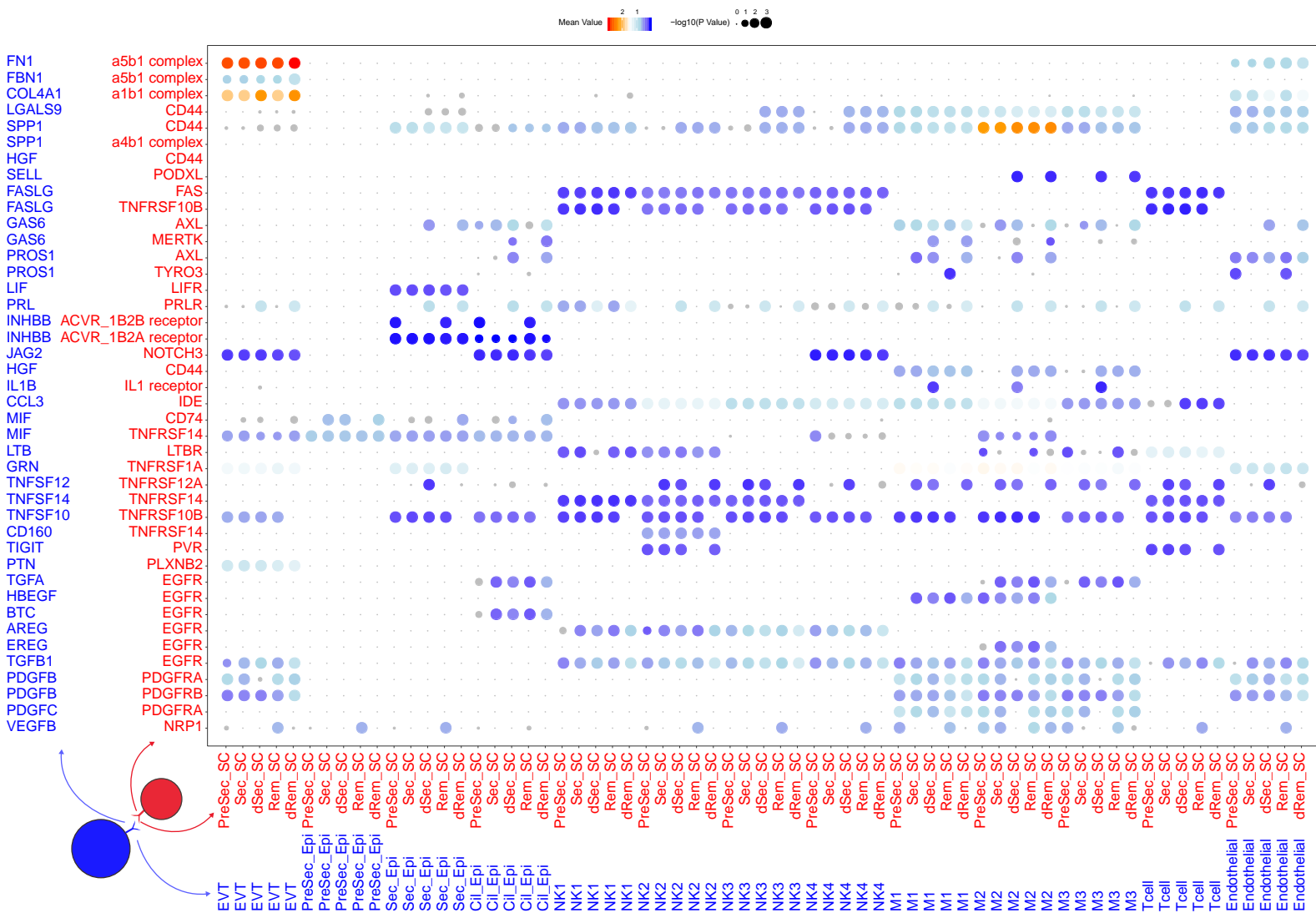

Supplement: Supplementary file 23 — Additional file 23. [file 12915_2022_1483_MOESM23_ESM.pdf]

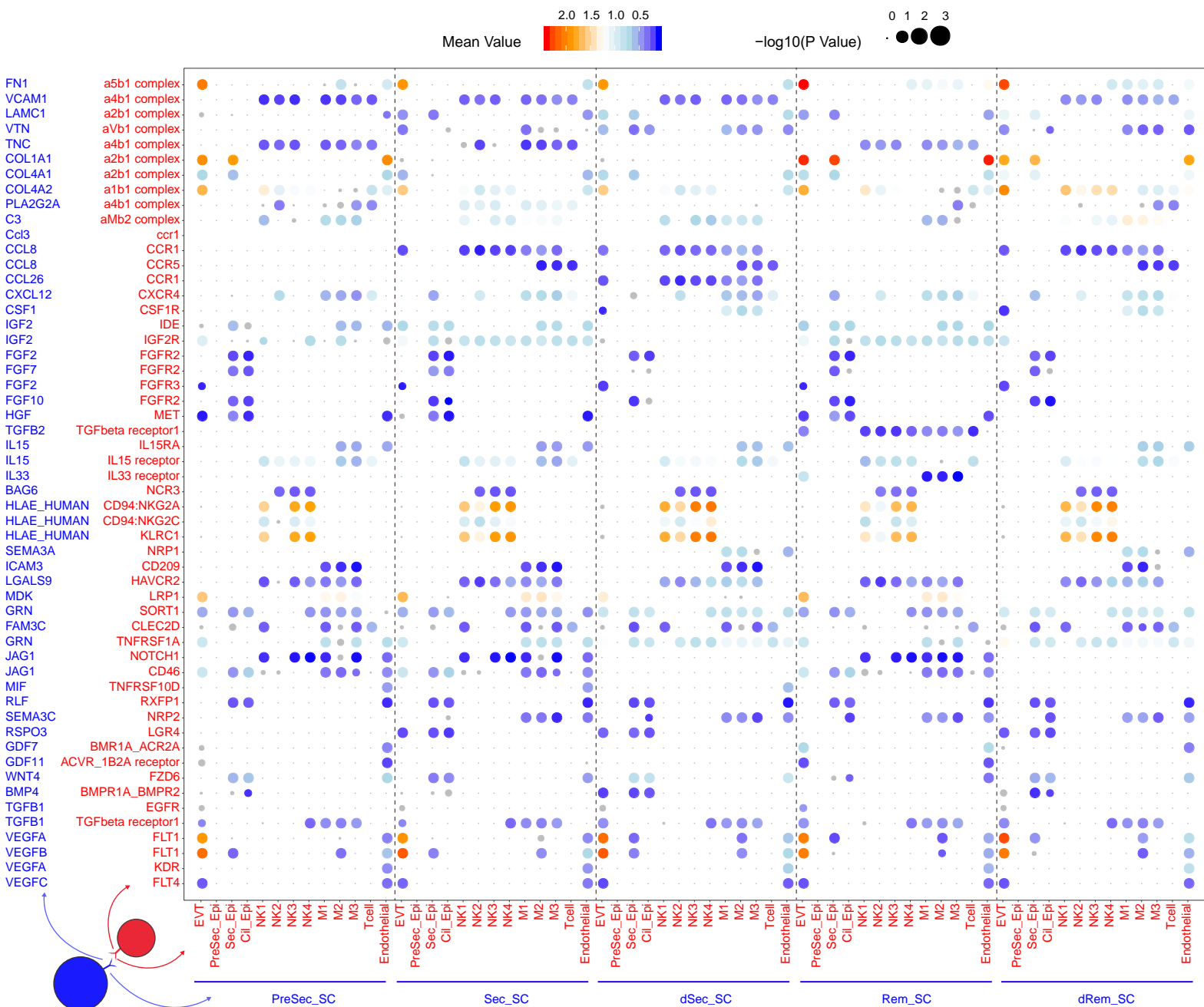

Supplement: Supplementary file 24 — Additional file 24. [file 12915_2022_1483_MOESM24_ESM.pdf]
