# Supplementary material for: An IGF1-expressing endometrial stromal cell population is associated with human decidualization
Source: BMC Biol. 2022 Dec 8;20:276. doi: 10.1186/s12915-022-01483-0 (PMC9733393; doi:10.1186/s12915-022-01483-0)
Supplement: Supplementary file 5 — Additional file 5. [file 12915_2022_1483_MOESM5_ESM.pdf]

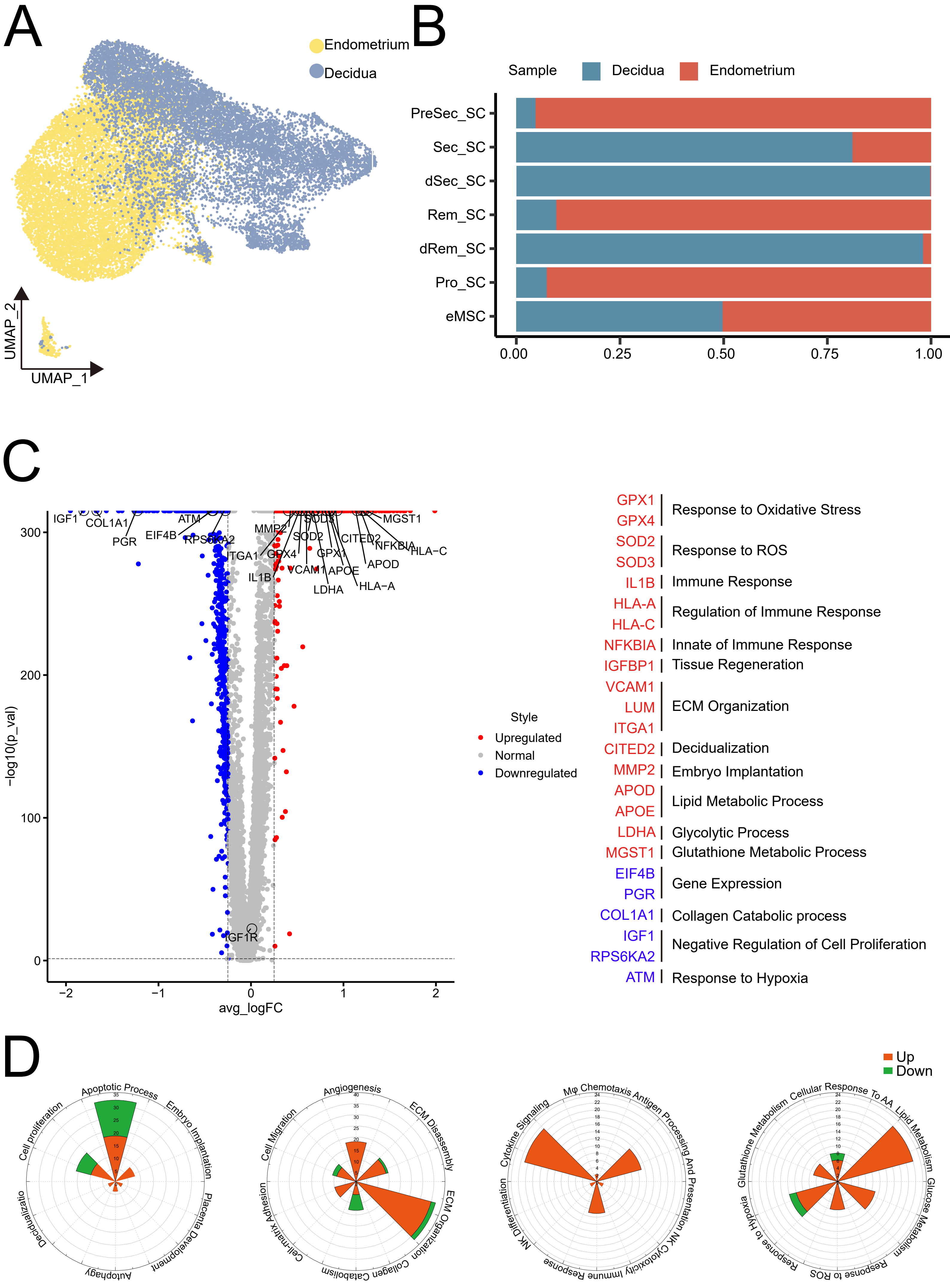
A
Endometrium
Decidua
UMAP_2
UMAP_1
B
Sample
Decidua
Endometrium
PreSec_SC
Sec_SC
dSec_SC
Rem_SC
dRem_SC
Pro_SC
eMSC
0.00
0.25
0.50
0.75
1.00
C
−log10(p_val)
avg_logFC
IGF1
COL1A1
PGR
EIF4B
ATM
RPS6KA2
ITGA1
IL1B
MMP2
GPX4
SOD2
SOD3
VCAM1
LDHA
GPX1
APOE
CITED2
APOD
NFKBIA
HLA−C
HLA−A
MGST1
IGF1R
Style
Upregulated
Normal
Downregulated
GPX1
GPX4
Response to Oxidative Stress
SOD2
SOD3
Response to ROS
IL1B
Immune Response
HLA-A
HLA-C
Regulation of Immune Response
NFKBIA
Innate of Immune Response
IGFBP1
Tissue Regeneration
VCAM1
LUM
ITGA1
ECM Organization
CITED2
Decidualization
MMP2
Embryo Implantation
APOD
APOE
Lipid Metabolic Process
LDHA
Glycolytic Process
MGST1
Glutathione Metabolic Process
EIF4B
PGR
Gene Expression
COL1A1
Collagen Catabolic process
IGF1
RPS6KA2
Negative Regulation of Cell Proliferation
ATM
Response to Hypoxia
D
Up
Down
Apoptotic Process
Embryo Implantation
Placenta Development
Autophagy
Decidualizatio
Cell proliferation
Angiogenesis
ECM Disassembly
ECM Organization
Collagen Catabolism
Cell-matrix Adhesion
Cell Migration
Mφ Chemotaxis
Antigen Processing And Presentation
NK Cytotoxicity
Immune Response
NK Differentiation
Cytokine Signaling
Cellular Response To AA
Lipid Metabolism
Glucose Metabolism
Response to ROS
Response to Hypoxia
Glutathione Metabolism
