# Supplementary material for: An IGF1-expressing endometrial stromal cell population is associated with human decidualization
Source: BMC Biol. 2022 Dec 8;20:276. doi: 10.1186/s12915-022-01483-0 (PMC9733393; doi:10.1186/s12915-022-01483-0)
Supplement: Supplementary file 25 — Additional file 25. [file 12915_2022_1483_MOESM25_ESM.docx]

**Table S1. Primer sequences of related genes.**

| ACTB | Forward GCCGACAGGATGCAGAAGGAGATCA  Reverse AAGCATTTGCGGTGGACGATGGA |
| --- | --- |
| IGF1 | Forward TGTCCTCCTCGCATCTCTTCTACC  Reverse CCTGTCTCCACACACGAACTGAAG |
| IGF1R | Forward TCGACATCCGCAACGACTATC  Reverse CCAGGGCGTAGTTGTAGAAGAG |
| PRL | Forward AAGCTGTAGAGATTGAGGAGCAAAC  Reverse TCAGGATGAACCTGGCTGACTA |
| PRLR | Forward GAGTCCAGCGACCTTCATTCAGATAC  Reverse CCTTCAAAGCCACTGCCCAGAC |
| IGFBP1 | Forward CGAAGGCTCTCCATGTCACCA  Reverse TGTCTCCTGTGCCTTGGCTAAAC |
| IGFBP4 | Forward GGTGACCACCCCAACAACAG  Reverse GAATTTTGGCGAAGTGCTTCTG |
| IGFBP5 | Forward ACCTGAGATGAGACAGGAGTC  Reverse GTAGAATCCTTTGCGGTCACAA |
| FBN1 | Forward TTTAGCGTCCTACACGAGCC  Reverse CCATCCAGGGCAACAGTAAGC |
| LAMC1 | Forward GGACTCCGCCCGAGGAATA  Reverse ACTTGAGACGCACATAGGTGA |
| LUM | Forward TAACTGCCCTGAAAGCTACCC  Reverse GGAGGCACCATTGGTACACTT |
| P4HA3 | Forward GCTGCGGGACCTGACTAGA  Reverse CAAGCAGAGGGTTAGCCACAG |
| P4HB | Forward GGCTATCCCACCATCAAGTTC  Reverse TCACGATGTCATCAGCCTCTC |
| COL1A1 | Forward GAGGGCCAAGACGAAGACATC  Reverse CAGATCACGTCATCGCACAAC |
| COL1A2 | Forward GTTGCTGCTTGCAGTAACCTT  Reverse AGGGCCAAGTCCAACTCCTT |
| COL4A1 | Forward GGGATGCTGTTGAAAGGTGAA  Reverse GGTGGTCCGGTAAATCCTGG |
| COL4A2 | Forward TGAACCTGGAGAGCCTGGATTGG  Reverse GTCCTCTGTTGCCTTGCTGTCC |
| COL4A3 | Forward AGCAAGGGTTGTGTCTGTAAAG  Reverse CAGAAAATCCTGGCAATCCACT |
| COL4A4 | Forward GTGCCGTTAAAGGTATTCAGGG  Reverse GTGGCTCTACCAACAGGGT |
| COL4A5 | Forward TTGGCTGGCAACTGTAGATGTGTC  Reverse CGTGTCCTCAAGTCTCCTGCTTTC |
| COL4A6 | Forward GGTTGTGGCTGCTCCTGGTTAC  Reverse TCGTCCTCTCGCTCCTTTCTCAG |
| GPX1 | Forward CAGTCGGTGTATGCCTTCTCG  Reverse GAGGGACGCCACATTCTCG |
| GPX4 | Forward GAGGCAAGACCGAAGTAAACTAC  Reverse CCGAACTGGTTACACGGGAA |
| MAOA | Forward GAATCAAGAGAAGGCGAGTATCG  Reverse GGCAGCAGATAGTCCTGAAATG |
| FOXO1 | Forward TCGTCATAATCTGTCCCTACACA  Reverse CGGCTTCGGCTCTTAGCAAA |
| CAV1 | Forward GCGACCCTAAACACCTCAAC  Reverse ATGCCGTCAAAACTGTGTGTC |
| VPS13D | Forward TACCGCCTCCGTAGTTACAAG  Reverse GTAAAGTGCAATCGACATCCCA |
| LCP1 | Forward GATCAGTGTCCGATGAGGAAATG  Reverse CCAGATCACCTGTAGCCATCA |
| CD55 | Forward AGGCCGTACAAGTTTTCCCG  Reverse CCTTCTCGCCAGGAATTTTCAC |
| CXCL14 | Forward CGCTACAGCGACGTGAAGAA  Reverse GTTCCAGGCGTTGTACCAC |
| ADCY1 | Forward AGGCACGACAATGTGAGCATC  Reverse AGGCACGACAATGTGAGCATC |
| ADCY3 | Forward AGAACCTCTACCAGACCTACTTC  Reverse CCACCACGTAGCAGTCAAAGA |
| IL1B | Forward ATGATGGCTTATTACAGTGGCAA  Reverse GTCGGAGATTCGTAGCTGGA |
| MMP2 | Forward GTGATCTTGACCAGAATACC  Reverse GCCAATGATCCTGTATGTG |
| MMP9 | Forward CAGTACCGAGAGAAAGCCTATT  Reverse CAGGATGTCATAGGTCACGTAG |
| LIF | Forward CCAACGTGACGGACTTCCC  Reverse TACACGACTATGCGGTACAGC |
| DPP4 | Forward AGTGGCACGGCAACACATT  Reverse AGAGCTTCTATCCCGATGACTT |
| AHNAK | Forward TACCCTTCCTAAGGCTGACATT  Reverse TTGGACCCTTGAGTTTTGCAT |
| ESR1 | Forward CCCACTCAACAGCGTGTCTC  Reverse CGTCGATTATCTGAATTTGGCCT |
| PGR | Forward ACCCGCCCTATCTCAACTACC  Reverse AGGACACCATAATGACAGCCT |
| APOD | Forward CTTGGGAAGTGCCCCAATCC  Reverse TGCCGATGGCATAAACCAGG |
| IMPA2 | Forward GGGCAGGACAGATCATCAGAA  Reverse GAAACCTCTCTCGCAACTCAG |
| FBLN2 | Forward CAATCATGTCATGCTCTCCTGCT  Reverse ATCTCTGCCTCTGAAACTCTCCG |
