# Supplementary material for: An IGF1-expressing endometrial stromal cell population is associated with human decidualization
Source: BMC Biol. 2022 Dec 8;20:276. doi: 10.1186/s12915-022-01483-0 (PMC9733393; doi:10.1186/s12915-022-01483-0)
Supplement: Supplementary file 26 — Additional file 26. [file 12915_2022_1483_MOESM26_ESM.docx]

**Table S2. Human antibodies for flow cytometry assays.**

| Allophycocyanin (APC)-Cy7 anti-human CD45 antibody (304014; Biolegend, CA, USA) |
| --- |
| FITC anti-human CD3 antibody (317306; Biolegend, CA, USA) |
| Brilliant Violet® 421 (BV421) anti-human CD56 antibody (562751; BD Pharmingen, USA) |
| BV421 anti-human CD14 antibody (565283; BD Pharmingen, USA) |
| APC anti-human AREG (17-5370-42; Invitrogen, USA) |
| Human CSF1 (M-CSF) PE-conjugated Antibody (IC2161P; R&D Systems, USA) |
| Human SPP1 (Osteopontin/OPN) PE-conjugated Antibody (IC14331P; R&D Systems, USA) |
| FITC anti-human CX3CR1 Antibody (341605; Biolegend, CA, USA) |
| PE/Cyanine7 anti-human CD160 Antibody (341212; Biolegend, CA, USA) |
| APC anti-human TNFRSF4 (CD134 (OX40)) Antibody (350008; Biolegend, CA, USA) |
| PE anti-human ITGAX (CD11c) Antibody (371504; Biolegend, CA, USA) |
